# Recovery of health-related quality of life after burn injuries: An individual participant data meta-analysis

**DOI:** 10.1371/journal.pone.0226653

**Published:** 2020-01-10

**Authors:** Inge Spronk, Nancy E. E. Van Loey, Charlie Sewalt, Daan Nieboer, Babette Renneberg, Asgjerd Litleré Moi, Caisa Oster, Lotti Orwelius, Margriet E. van Baar, Suzanne Polinder, the Quality of life study group

**Affiliations:** 1 Erasmus MC, University Medical Center Rotterdam, Department of Public Health, Rotterdam, the Netherlands; 2 Association of Dutch Burn Centres, Maasstad Hospital, Rotterdam, the Netherlands; 3 Amsterdam UMC, Vrije Universiteit Amsterdam, Department of Plastic, Reconstructive and Hand Surgery, Amsterdam Movement Sciences, Amsterdam, Netherlands; 4 Association of Dutch Burn Centres, Department Behavioural Research, Beverwijk, the Netherlands; 5 Utrecht University, Department Clinical Psychology, Utrecht, the Netherlands; 6 Freie Universität Berlin, Department of Clinical Psychology and Psychotherapy, Berlin, Germany; 7 Western Norway University of Applied Sciences, Department of Health and Caring Sciences, Faculty of Health and Social Sciences, Bergen, Norway; 8 National Burn Centre, Haukeland University Hospital, Department of Plastic, Hand and Reconstructive Surgery, Bergen, Norway; 9 Uppsala University, Department of Neuroscience, Psychiatry, Sweden; 10 Linköping University, Department of Anaesthesiology and Intensive Care, and Department of Clinical and Experimental Medicine, Linköping, Sweden; Monash University, AUSTRALIA

## Abstract

**Background:**

A prominent outcome measure within burn care is health related quality of life (HRQL). Until now, no model for long-term recovery of HRQL exists for adult burn patients which requires large samples with repeated measurements. Re-use and the combination of existing data is a way to achieve larger data samples that enable the estimation of long-term recovery models. The aim of this secondary data analysis was to assess the recovery of HRQL after a burn injury over time.

**Methods and findings:**

Data from ten European studies on generic HRQL assessed in adult burn patients (either with the EQ-5D or SF-36) from five different countries were merged into one dataset. SF-36 outcomes were transformed into EQ-5D outcomes. A 24-month recovery of HRQL (EQ-5D utility) was modeled using a linear mixed-effects model and adjusted for important patient and burn characteristics. Subgroups of patients with mild and intermediate burns (≤20% total body surface area (TBSA) burned) and with major burns (>20% TBSA burned) were compared. The combined database included 1687 patients with a mean age of 43 (SD 15) years and a median %TBSA burned of 9% (IQR 4–18). There was large improvement in HRQL up to six months after burns, and HRQL remained relatively stable afterwards (studied up to 24 months post burn). However, the estimated EQ-5D utility scores remained below the norm scores of the general population. In this large sample, females, patients with a long hospital stay and patients with major burns had a delayed and worse recovery. The proportion of patients that reported problems for the EQ-5D dimensions ranged from 100% (pain/discomfort at baseline in patients with major burns) to 10% (self-care ≥3 months after injury in patients with mild and intermediate burns). After 24 months, both subgroups of burn patients did not reach the level of the general population in the dimensions pain/discomfort and anxiety/depression, and patients with major burns in the dimension usual activities. A main limitation of the study includes that the variables in the model were limited to age, gender, %TBSA, LOS and time since burn as these were the only variables available in all datasets.

**Conclusions:**

The 24-month recovery model can be used in clinical practice to inform patients on expected HRQL outcomes and provide clinicians insights into the expected recovery of HRQL. In this way, a delayed recovery can be recognized in an early stage and timely interventions can be started in order to improve patient outcomes. However, external validation of the developed model is needed before implementation into clinical practice. Furthermore, our study showed the benefit of secondary data usage within the field of burns.

## Introduction

Improved survival of burn patients has led to an increased focus on factors that influence recovery and outcomes after burn injuries [1]. A prominent outcome measure within burn care is health related quality of life (HRQL) [2]. This patient-reported outcome measure reflects a patient’s physical, psychological, and social well-being [3]. Many burn patients experience functional limitations shortly after burns [2]. During rehabilitation, most limitations improve, however, some remain highly prevalent in a subset of patients in the long-term, like psychological functioning [2]. Moreover, some limitations, like participation restrictions due to mental well-being, seem to develop later [2, 4]. Early identification of patients at risk for chronic problems may assist burn clinicians to tailor care and to prevent long term problems.

The development of a recovery model for HRQL in burn patients may facilitate the early identification of persons with a delayed recovery and timely interventions can be started in order to improve patient outcomes. Moreover, a recovery model can be used to inform patients on expected outcomes. Such models have shown to improve evaluation of outcomes and can be used to evaluate quality of care [5]. Recovery models for HRQL have been developed for children and young adults with burns [6–8]. However, such a model does not exist for adult burn patients [2, 9]. Information on the expected recovery of HRQL over time in adult burn patients adjusted for factors that showed to influence HRQL outcomes after burn injuries, including age, gender, percentage total body surface area (%TBSA) burned and length of stay (LOS) can inform the burn clinicians [2, 10].

In recent years, the re-use (secondary use) of existing data has been promoted. Researchers are encouraged to create data-sharing plans and share their data on existing repositories so that other researchers are able to re-use the existing data [11, 12]. Secondary analysis of existing data is time-saving and cost-efficient; the data is already collected and checked by another researcher, and it is of low risk to participants as data are anonymously shared [13–15]. An additional advantage, which is especially valuable in burns, is the creation of a larger database when existing databases are combined. This increases power to perform (subgroup) analyses and might create the opportunity to study the long-term recovery of HRQL. Combining existing datasets might be a valuable option to study recovery of HRQL in burns.

The aim of this study was to model recovery of HRQL after burn injuries adjusted for age, gender, %TBSA and LOS. Data from ten European studies collected in five different countries on generic HRQL in burn patients were combined into one large database to increase statistical power that allows subgroup analyses.

## Methods

### Data collection

Data from ten European studies on HRQL, both trial and cohort studies, which investigated generic HRQL with either the EuroQol—5 Dimensions (EQ-5D) or Medical Outcome Study Short Form—36 items (SF-36) was collected (Table 1). Patients from the different datasets were included if they completed at least one HRQL survey. Data include HRQL outcomes, as well as demographic and injury-related data (assessment method, age, gender, %TBSA burned, length of stay (LOS), aetiology of burn, number of surgeries, assessment time points). A data agreement form in which the aim of the study was explained and which stated the preconditions on how data was shared (e.g. anonymously) and for which aim the data was used, was signed by all principal investigators. In all studies, patients with cognitive impairment and poor language proficiency were excluded. All studies were conducted in accordance with the Declaration of Helsinki and all participants provided written informed consent before inclusion in the different studies. The study of Oster et al. and of Orwelius et al. included some identical patients (n = 54). Because these studies included the same measurement points, the 54 patients were removed from the dataset of Orwelius et al. as this study had the shortest follow-up. All other studies are unique cohort studies.

**Table 1:** Characteristics of included patients from the different studies.

First author, year (reference)	Country	Inclusion criteria	Design	Study population	Etiology	%TBSA^1^ burned, mean (SD)	LOS^2^, mean (SD)	No of surgery, mean (SD)	HRQL instrument	Assessment time point(s)
Bloemen et al, 2012 [32]	The Netherlands	Surgery and TBSA full thickness burns <15%, study wound surface area min. 10 cm^2^ and max. 300 cm^2^ (October 2007 –February 2010)	Trial	n = 77, (M: 57.1%). Mean age: 47.4yr	Flame: 72.3% Scald: 15.4%	8.3% (7.7)	19.9 (15.2)	1.5 (0.9)	EQ-5D-3L	3, 12 months
Hop et al, 2013 [33]	The Netherlands	Outpatient or admitted to a burn centre within 5 days post burn, with burns of indeterminate depth and a ≤20% TBSA burned (August 2011 –July 2013)	Trial	n = 124, (M: 69.4%). Mean age: 42.3yr	Flame: 54.0% Scald: 24.2%	8.0% (11.9)	18.4 (24.8)	1.0 (1.5)	EQ-5D-3L	3, 12, 24 months
Moi et al, 2006 [31]	Norway	All patients hospitalized for burn injury (1995–2000)	Cohort	n = 90, (M: 83.3%). Mean age: 43.0yr	Flame: 57.8% Scald: 24.4%	17.7% (12.8)	22.7 (20.3)	1.7 (1.9)	SF-36	Measurement 1: 11–82 months Measurement 2^3^: 150–220 months [36]
Orwellius et al, 2013 [24]	Sweden	Burn patients with ≥10% TBSA burned or LOS of ≥7 days (March 2000 –December 2009)	Cohort	n = 118, (M: 77.1%). Mean age: 48.2yr	NA	23.3% (17.6)	29.8 (32.4)	NA	EQ-5D-3L	12 and 24 months
Oster et al, 2011 [4]	Sweden	Burn patients with ≥5% TBSA burned or LOS of >1 day (March 2000 –March 2007)	Cohort	n = 67, (M: 77.6%). Mean age: 42.6yr	Flame: 74.6% Scald: 10.4%	25.6% (20.2)	26.9 (33.5)	NA	EQ-5D-3L	Admission, 3, 6, 12, 24 months, 2–7 years (mean 4.6yr)
Renneberg et al, 2014 [25]	Germany	All patients hospitalized in the burn unit (June 2004 and November 2006)	Cohort	n = 292, (M: 72.3%). Mean age: 39.6yr	NA	15.0% (14.2)	28.1 (31.2)	2.6 (4.8)	SF-36	6, 12, 24, 36 months
Spronk et al, 2019 [10]	The Netherlands	Burn patients with LOS of ≥1 day or with surgery (2010–2013)	Cohort	n = 256, (M: 62.1%). Mean age: 47.7yr	Flame: 57.9% Scald: 18.5%	9.6% (16.9)	17.5 (22.0)	1.3 (1.9)	EQ-5D-5L	5–7 years (mean 5.5yr)
Van Loey et al, 2012 [9]	Belgium and The Netherlands	Burn patients with LOS of ≥72 hours (March 2003 and April 2005)	Cohort	n = 257, (M: 72.4%). Mean age: 38.9yr	Flame: 57.3% Scald: 24.9%	12.7% (11.5)	24.2 (23.0)	1.5 (2.2)	EQ-5D-3L	3 weeks, 3, 9, 18 months
Hoogewerf et al, 2014 [35]^4^	Belgium and The Netherlands	Burn patients with LOS of ≥72 hours (March 2006 –January 2009)	Cohort	n = 297, (M: 79.8%). Mean age: 40.8yr	Flame: 65.1% Scald: 22.3%	12.9% (12.1)	22.6 (20.9)	1.1 (1.7)	SF-36	3 and 18 months
Bosmans et al, 2015 [34]	Belgium and The Netherlands	Burn patients with TBSA≥1% burned or LOS≥48 hours (April 2010 –October 2012)	Cohort	n = 145, (M: 65.5%). Mean age: 40.6yr	Flame: 58.3% Scald: 30.9%	9.0% (8.0)	17.2 (13.2)	0.9 (1.5)	EQ-5D-3L	2 weeks, 3, 6, 12, 18 months

*Notes*.

^*1*^TBSA = total body area burned

^2^LOS = length of hospital stay

^3^Not included in the combined dataset as outcomes of ≤10 patients were available per time point (aggregated on a 12-month level). Results are published in Moi et al., 2016

^4^The reference includes a selection (patients with facial burns) of the total cohort.

### Health-related quality of life outcome measures

The EQ-5D includes five dimensions to measure HRQL: mobility, self-care, usual activities, pain/discomfort and anxiety/depression and a visual analogue scale (EQ-VAS) for general health [16].

Each dimension has three answer options: no problems, some problems, severe problems [17]. Based on the answers of the five dimensions and the value set of the United Kingdom population, a utility score was derived ranging from 0 (death) to 1 (full health) [18]. The EQ-5D utility score can also have negative values (lowest possible: -0.059) for health states worse than death. Two versions of the EQ-5D exist, the 3L (three answer options) and the 5L (five answer options) [16, 19]. The EQ-5D has good psychometric properties in burn populations [20].

In order to analyze all data together, SF-12 data was transformed to EQ-5D data by applying the algorithm of Gray et al. [21]. First, the 12 items composing the SF-12 were extracted from the SF-36 data [22]. Subsequently, these items were used to estimate the EQ-5D outcomes (both the EQ-5D utility scores as well as the five different dimensions) by applying the algorithm [21]. As the algorithm of Gray et al. does not include the EQ-VAS, this outcome was not studied. The EQ-5D-5L data was mapped into EQ-5D-3L data using the method of Van Hout et al. [23]. After transformation of SF-12 and EQ-5D-5L outcomes into EQ-5D-3L outcomes, the data were merged into one large combined HRQL dataset that was used for the analyses (S1 Appendix).

### Data-analyses

R software (version 3.5) was used for all data analysis. Descriptive statistics were used to analyze patient and clinical characteristics as well as EQ-5D scores. Mean and standard deviation (SD) were used for continuous variables and frequencies and percentages for categorical variables. We evaluated HRQL outcomes up to 84 months. After that period, outcomes of ≤10 patients were available per time point (aggregated on a 12-month level), a number that was deemed too small to provide reliable and representative outcomes. Missing values existed for the variables %TBSA (n = 22), LOS (n = 12), etiology of burn (n = 438) and number of surgeries (n = 184). Two studies did not provide information regarding number of surgeries [4, 24], and two did not provide information on etiology [24, 25]. Missing data for the variables etiology and number of surgeries was therefore not imputed. Only the sporadically missing values for %TBSA and LOS were imputed. Missing data was imputed in each study cohort separately and combined afterwards [26]. Missing outcome data was not imputed [27]. We used single imputation for our analyses. Imputations were performed using the aregImpute from the Hmisc package in R. The first set of imputations was used for the analyses.

First, we examined characteristics and outcomes of the different studies separately and compared changes in EQ-5D utility scores over time graphically and compared them with the UK norm scores [28]. Second, we examined characteristics of the combined dataset and the combined (average) EQ-5D utility scores and dimension scores across all data in the combined dataset. We did this separately for the group of patients with minor burns (≤20% TBSA burned) and the group of patients with major burns (>20% TBSA). This grouping was based on the criteria of the American Burn Association [29]. Scores were examined over time and compared with the UK norm sores graphically. Lastly, we modeled the EQ-5D utility scores over time using a linear mixed-effects model [30]. Because we included data from different studies, the variable ‘study’ was included as a random intercept, in order to adjust for potential residual confounding between studies. Also, ‘patient’ was added as a random intercept as most patients had more than one HRQL outcome (one outcome at each of the different measurement points). The model was adjusted for the characteristics available in all studies, being time since burn, age, gender, %TBSA burned and LOS. These variables were included as fixed effects. Furthermore, we included the interaction terms of each of these characteristics with time as a longitudinal model was created. A p-value of <0.05 was considered statistically significant.

## Results

HRQL data from the ten different studies originated from five countries, namely Belgium, Germany, Norway, Sweden and The Netherlands [4, 9, 10, 24, 25, 31–35]. Data was collected between 1995 and 2018. Six studies used the EQ-5D-3L, three the SF-36 and one the EQ-5D-5L to assess HRQL. Study characteristics are presented in Table 1.

### EQ-5D utility outcomes from the different studies

**Fig 1:**
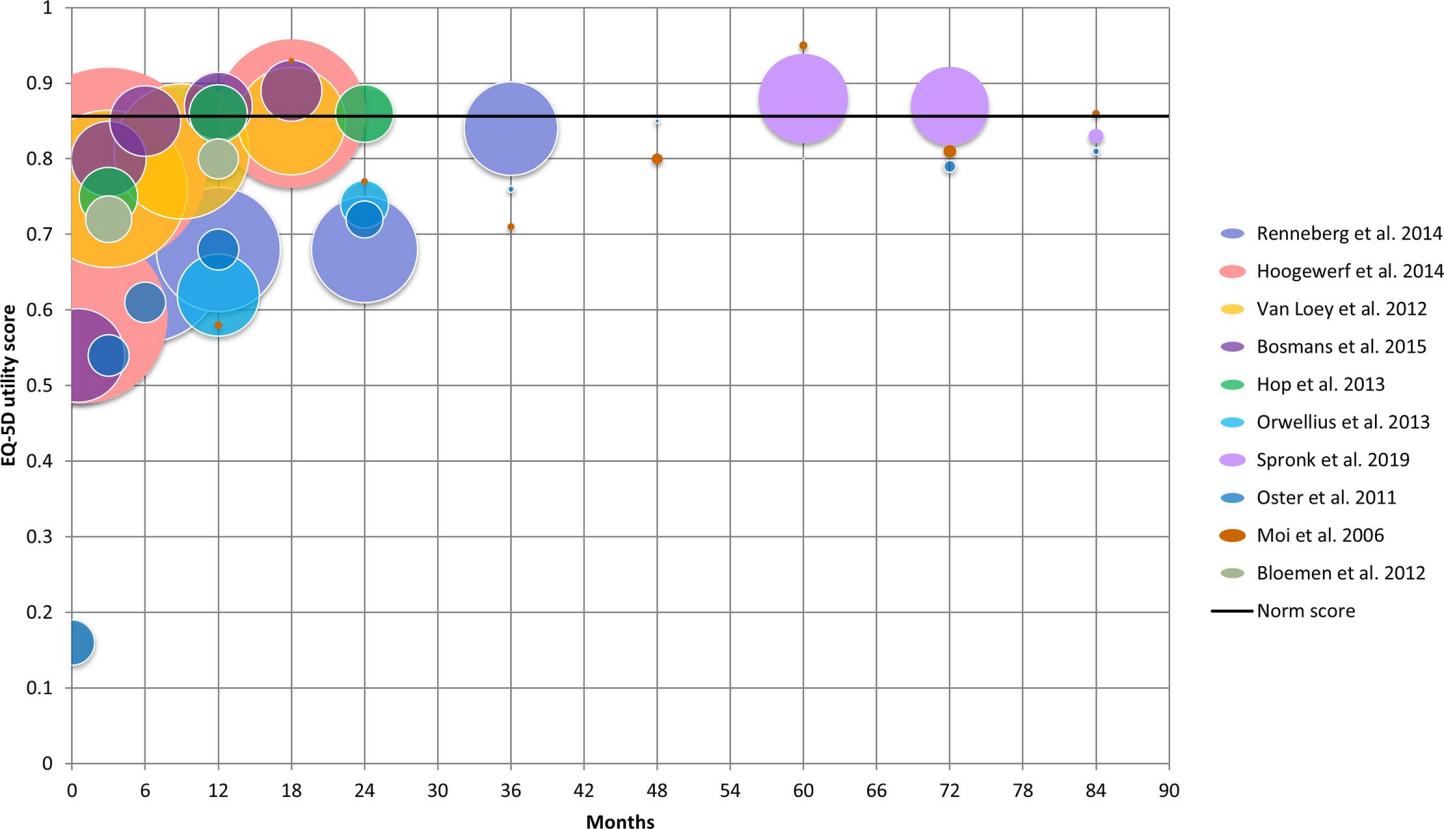
Raw outcomes of the different studies. The size of the bubble represents the number of outcomes within a study on a specific time point.

From most studies outcomes were available up to 24 months post burn. Four studies with a limited number of patients, assessed HRQL beyond this period (Fig 1). There was a wide variation in outcomes across the studies and time points. For example, at 3 months, EQ-5D utility scores ranged from 0.54 to 0.80, and at 12 months from 0.58 to 0.87. This can be partly explained by variation in study characteristics as shown in Table 1.

### Demographics of combined dataset

The combined HRQL dataset included assessments of 1687 patients (Table 2). These patients were predominantly male (72%) and the mean age was 43 years old (SD: 15, range: 18–90 years). The average length of stay (LOS) was 23 days (SD: 25, range: 0–246 days) and the median %TBSA burned was 9% (IQR: 4–18%, range: 0–90%). Most patients (n = 1,343; 80%) had mild/intermediate burns (≤20% TBSA burned) and 344 patients (20%) had major burns (>20% TBSA burned). Patients had on average 1.5 surgical procedures (SD: 2.7, range: 0–35). The majority of patients (67%) had at least one surgical procedure. The most common cause of burns was flames (61%). HRQL measurement time points ranged from admission to 220 months post burn. Most patient responses were available at 3 months (n = 815), 12 months (n = 601) and 18 months (n = 459) (S1 Appendix).

**Table 2:** Demographic characteristics of combined dataset.

Variable	Total sample	Patients with mild/intermediate burns (n = 1,343)	Patients with major burns (n = 344)
**Gender**			
Male, n(%)	1211 (71.8%)	947 (70.5%)	264 (67.7%)
**Age**			
Mean (SD)	42.5 (14.9)	42.8 (15.1)	41.3 (13.8)
Range	18–90 years	18–89 years	18–90 years
**%TBSA**			
Median (IQR)	9.0 (4.3–18.0)	7.0 (3.5–7.0)	31.0 (25.0–43.0)
Range	0–90%	0–20%	21–90%
**Length of hospital stay**			
Mean (SD)	23.1 (24.8)	16.7 (14.7)	48.3 (37.3)
Range	0–246 days	0–178 days	1–246 days
Nr of surgeries*			
Mean (SD)	1.5 (2.7)	1.0 (1.5)	4.1 (4.9)
Range	0–35 surgeries	0–18 surgeries	0–35 surgeries
Nr surgery, n(%)*			
0	501 (33.3%)	474 (38.2%)	27 (10.3%)
1	624 (41.6%)	559 (45.1%)	65 (24.7%)
>1	378 (25.1%)	207 (16.7%)	171 (65.0%)
Etiology (%)*			
Scald	281 (22.5%)	251 (24.4%)	30 (13.5%)
Contact	55 (4.4%)	52 (5.1%)	3 (1.4%)
Flame	760 (60.8%)	587 (57.2%)	173 (77.9%)
Chemical	59 (4.7%)	57 (4.2%)	2 (0.9%)
Electrical	56 (4.5%)	45 (4.4%)	11 (5.0%)
Other	38 (3.0%)	35 (2.6%)	3 (1.4%)

*Not all studies included information on number of surgery and/or etiology.

### Combined EQ-5D utility scores

**Fig 2:**
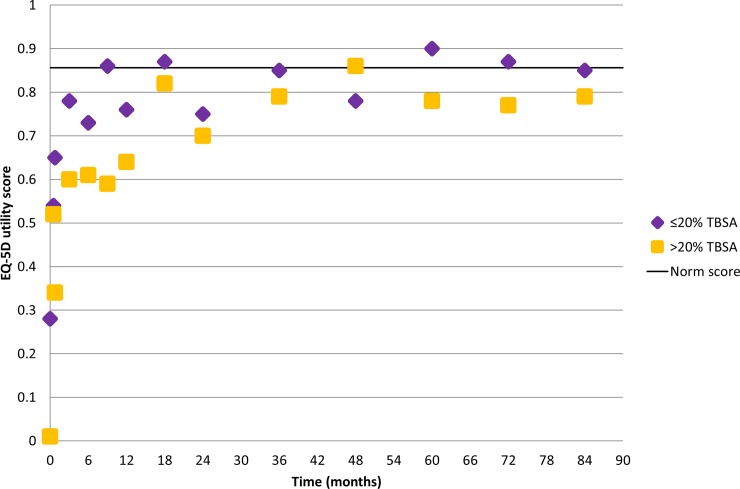
Combined EQ-5D utility scores for the subgroups of patients ≤20%TBSA burned and >20%TBSA burned compared with the norm score from the United Kingdom. The number of patients ranged between 13 and 680 for patients ≤20%TBSA burned; and between 10 and 148 for patients >20%TBSA burned.

Combined raw EQ-5D utility scores of the studies on the different time points are displayed in Fig 2, separately for the group of patients with mild and intermediate burns (≤20% TBSA burned) and the group of patients with major burns (>20% TBSA). These EQ-5D utility scores are not corrected for differences in patient characteristics across studies. The zigzag pattern in outcomes reflects the variation in measurement points across the studies (Fig 2). However, in general the outcomes show considerable variation, possibly because the studies used different time points (Table 1). However, a trend can be uncovered in all combined EQ-5D utility scores: for both patient groups the outcomes show large improvement up to six months after burns, a steady improvement up to 18 months and more or less a stable outcome thereafter. From that moment most combined outcomes remain stable with outcomes around the norm score for the group of patients with mild and intermediate burns and below the norm score for the group of patients with major burns. Also, the group of patients with major burns had a lower starting point.

### Combined EQ-5D dimension outcomes

**Fig 3:**
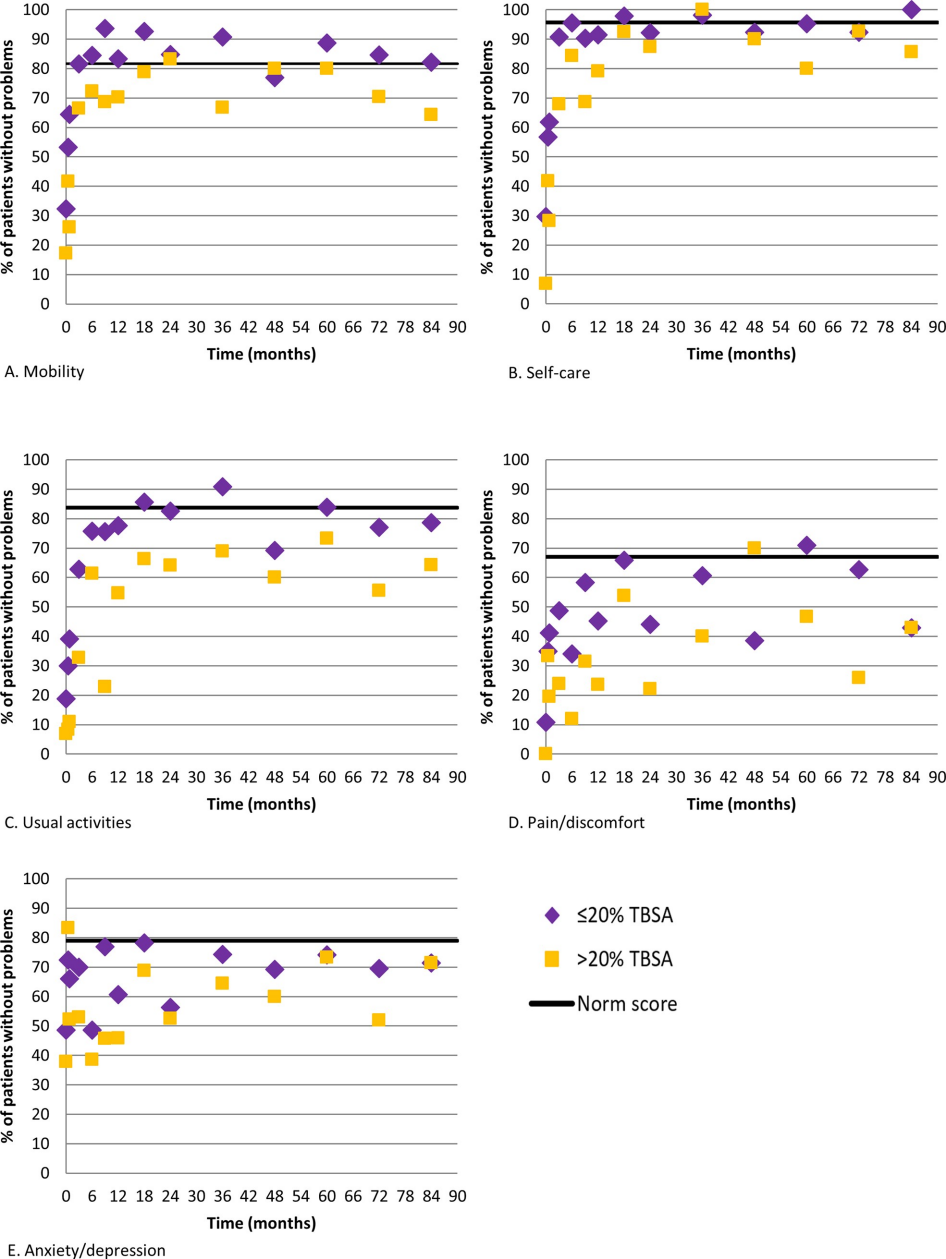
**A-E. Combined scores of percentages of patients without problems for the different dimensions of the EQ-5D for the subgroups of patients ≤20%TBSA burned and >20%TBSA burned, and norm scores from the United Kingdom.** The number of patients ranged between 13 and 680 for patients ≤20%TBSA burned; and between 10 and 148 for patients >20%TBSA burned.

The percentage of patients with problems was studied for each of the five EQ-5D dimensions and compared to the norm scores, separately for the group of patients with mild and intermediate burns and the group of patients with major burns (Fig 3A–3E). The most prevalent problem was pain/discomfort; almost all patients (89%) of patients with mild and intermediate burns and all patients with major burns experienced pain/discomfort at baseline (Fig 3D). Despite improvement over time, especially up to 12 months, problems with pain/discomfort remained far more prevalent in both patient groups compared to problems within the other dimensions, and also compared to the general population.

Problems with usual activities were also present in a large part of the patients; 81% of patients with mild and intermediate burns and 93% of patients with major burned had problems with usual activities at baseline (Fig 3C). Large improvement in usual activities was shown over time. In patients with mild and intermediate burns, with the proportion of patients having problems decreasing to about 30% at 6 months, and 15% at 18 months, after which the proportion of patients with problems stabilized. The problem level at 18 months was comparable to that of the general population. In the group of patients with major burns, also a large improvement was seen, with the proportion of patients having problems decreasing to about 40% at 6 months, however, thereafter the proportion of patients with problems stabilized. Problems with self-care were common at baseline in both patient groups (70% mild and intermediate burs; 93% major burns), however, major improvement was seen shortly after burns with about respectively 40% and 70% experiencing problems after one month and respective 10% and 20% after 6 months (Fig 3B). The percentage of patients with problems remained about these percentages during the studied course. Mobility improved rapidly from 70% (mild and intermediate burns) and 80% (major burns) at baseline down to about 15% and 30% six months later (Fig 3A). After three months, the problem level showed a tendency to stabilize in the group of patients with mild and intermediate burns, and improved towards 20% at 18 months in patients with major burns. This percentage stabilized after 18 months in this group. Compared to the norm scores, problems with mobility and self-care in the group of patients with mild and intermediate burns were more prevalent up to 3 months post burn, thereafter the proportion of patients experiencing problems was comparable to that in the general population. The group of patients with major burns reached this point about 18 months post-burn. Compared to the other dimensions, relatively few (about 30–40%) patients reported anxiety/depression problems in the first month after burns (Fig 3E). However, where the other dimensions showed large improvements, the percentage of patients with anxiety/depression problems remained more or less stable during the studied course, with a somewhat higher proportion of patient with major burns experiencing problems compared to patients with mild and intermediate burns. During the whole studied period problems with anxiety/depression remained more prevalent compared to the norm population in both patient groups.

### Estimated EQ-5D utility scores

**Fig 4:**
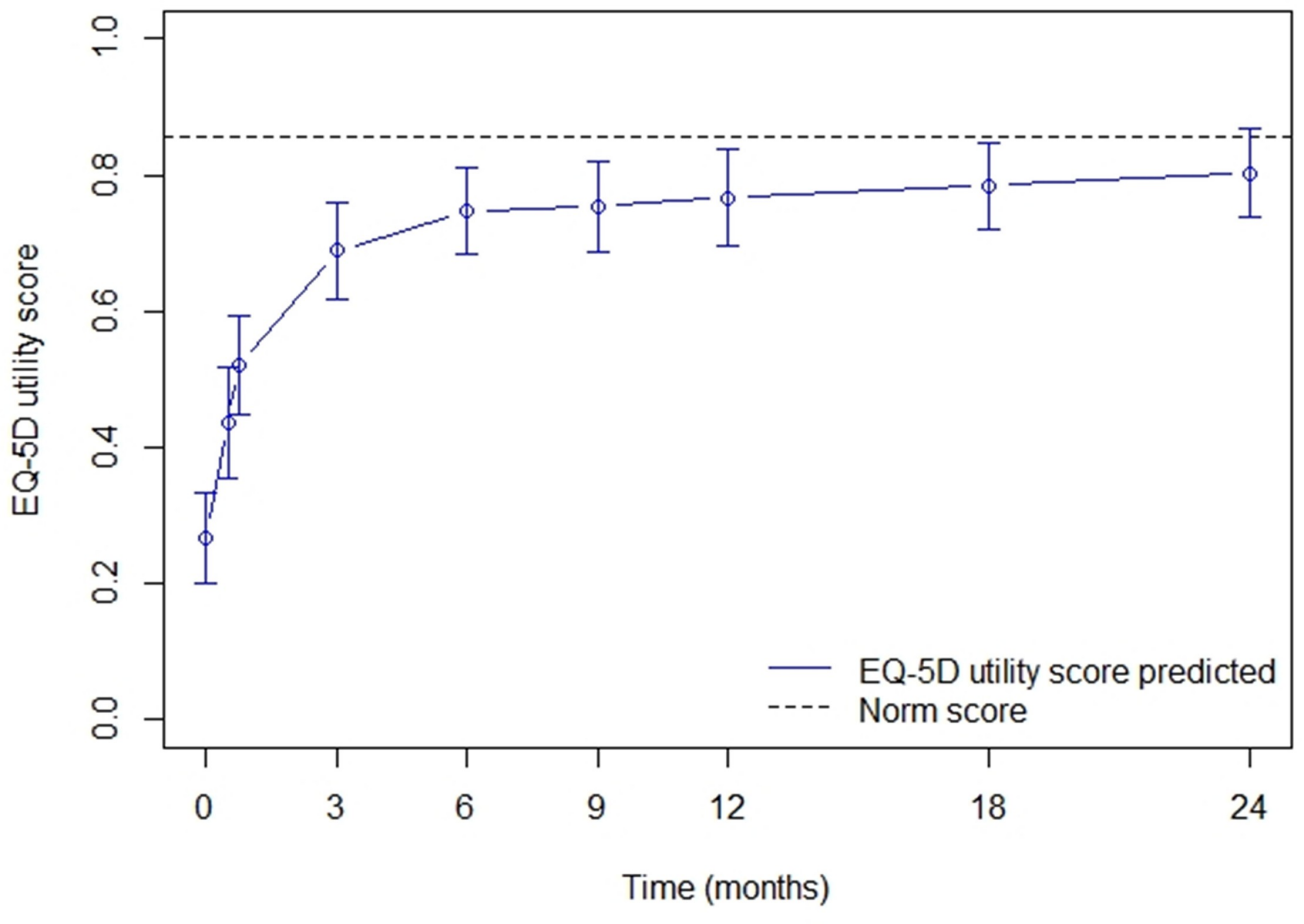
Estimated recovery of HRQL measured by EQ-5D utility scores over time.

EQ-5D utility recovery, adjusted for time since burn, age, gender, %TBSA burned and LOS, was estimated up to 24 months post-burn and displayed in Fig 4. The variables time since burn, age, gender, %TBSA burned and LOS, as well as the interaction between gender and time, LOS and time and %TBSA burned and time were significant factors associated with the EQ-5D utility score (S2 Appendix). A large improvement in HRQL was seen up to six months after burns, thereafter HRQL became more or less stable. The estimated EQ-5D utility scores on the different time points are presented in Table 3, ranging from 0.27 (SE 0.03) at baseline to 0.80 (SE 0.03) at 24 months. Up to 24 months post-burn, the estimated EQ-5D utility scores remained below the UK norm score of 0.856.

**Table 3:** Combined EQ-5D utility outcomes versus estimated EQ-5D utility outcomes.

Measurement point	No of studies contributing	No of patients	Combined EQ-5D utility scores^1^	Estimated EQ-5D utility scores (SE)^2^^,^^3^
Admission	1	66	0.16	0.27 (0.03)
2 weeks	1	132	0.54	0.44 (0.04)
3 weeks	1	241	0.59	0.52 (0.04)
3 months	6	815	0.75	0.69 (0.03)
6 months	3	379	0.70	0.75 (0.03)
9 months	1	191	0.81	0.75 (0.03)
12 months	7	597	0.73	0.77 (0.04)
18 months	4	458	0.86	0.78 (0.03)
24 months	5	365	0.74	0.80 (0.03)

^1^Combined EQ-5D utility scores: combined outcomes from the different studies together (without case-mix correction)

^2^Estimated EQ-5D utility scores: outcomes as estimated by the recovery model (with case-mix correction)

^3^SE = standard error

**Fig 5:**
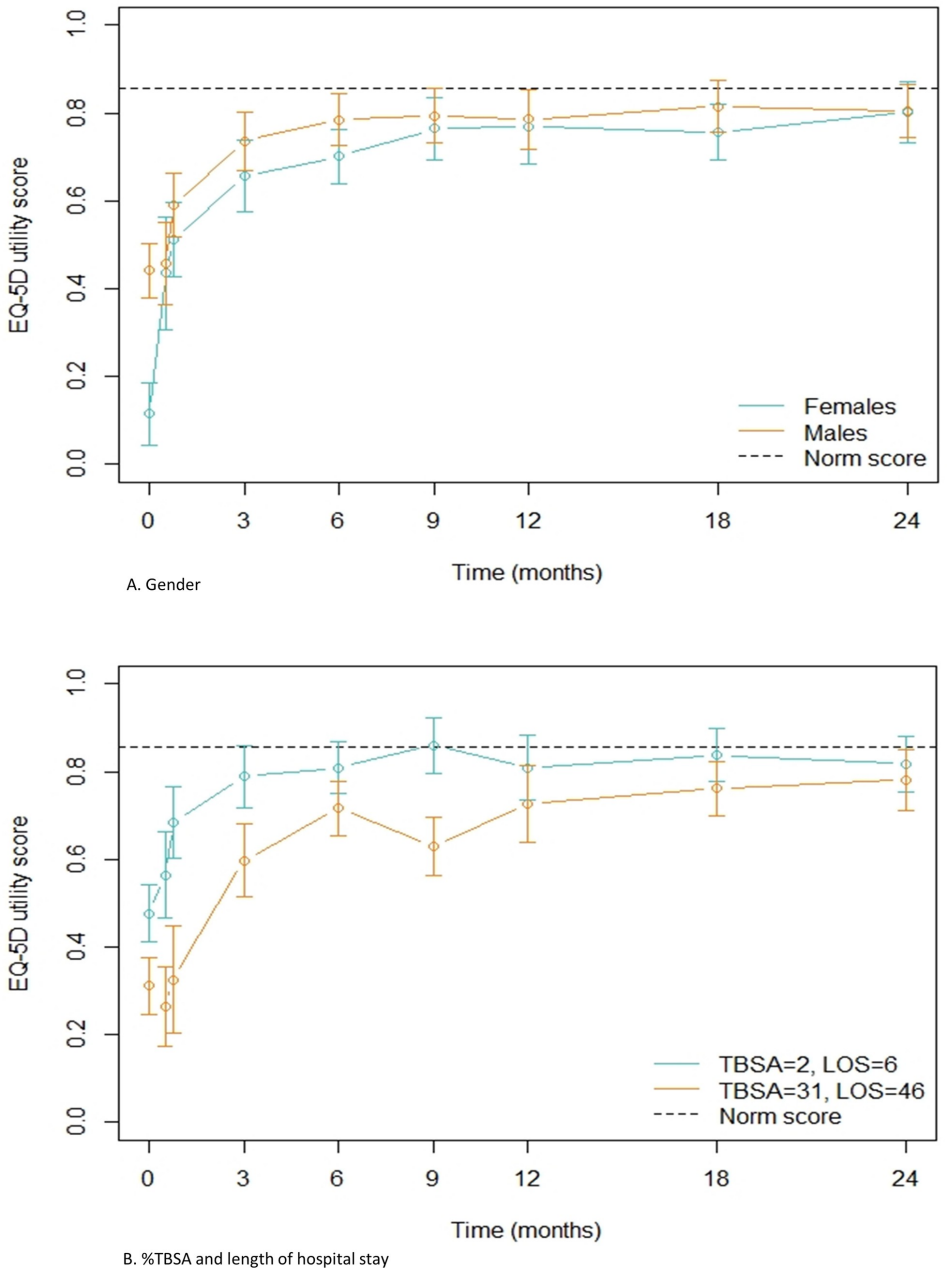
**Estimated recovery of HRQL measured by EQ-5D utility scores over time based on A) gender, B) %TBSA burned and length of hospital stay (LOS).** The figures show the estimated EQ-5D utility outcome for an average patient from our combined dataset, meaning that the median value of each variable was used: gender = male; age = 42.0 years; %TBSA burned = 9.0%; LOS = 17.0 days, with only the given variable changed for the specific Fig: for A) gender is male and female, for B) %TBSA = 2 and LOS = 6, and %TBSA = 31 LOS = 46.

The model showed that gender, LOS and %TBSA burned had an interaction with time. Females, patients with a long LOS and patients with a higher %TBSA burned have a worse and delayed recovery compared to males, patients with a short LOS and patients with a lower %TBSA burned (Fig 5A and 5B). Absolute differences between groups tended to become smaller as time progressed.

**Fig 6:**
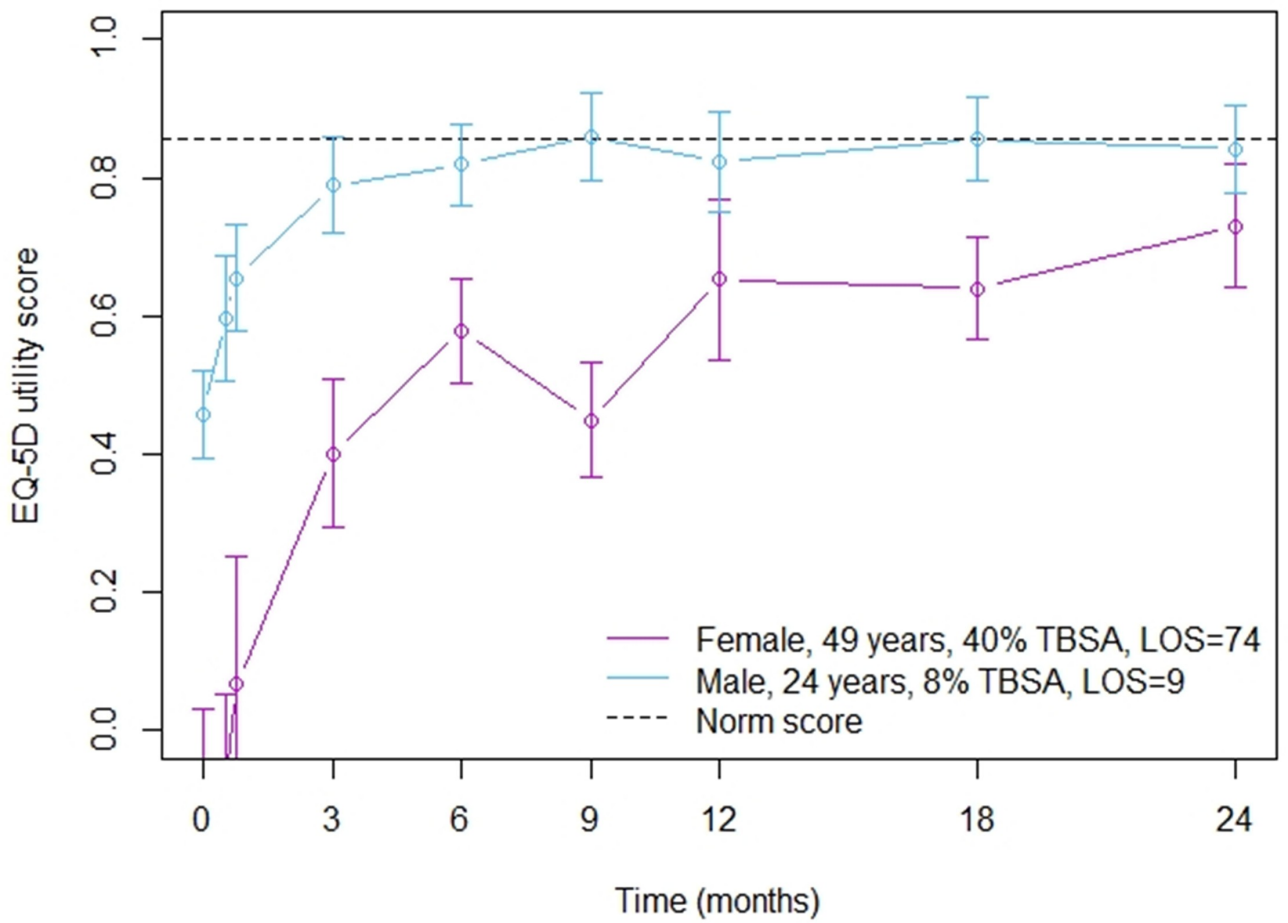
Example of estimated recovery of HRQL for two specific patients. This figure shows the estimated EQ-5D utility recovery for 1) a female, aged 49 years old, 40% TBSA and length of hospital stay (LOS) of 74 days, and 2) a male, aged 24 years old, 8% TBSA and LOS 9 days.

Fig 6 shows the practical application of the model. Two cases have been displayed; the estimated recovery of a 49-year old female with 40%TBSA and a LOS of 74 days, and the estimated recovery of a 24-year old male with 8%TBSa and a LOS of 9 days. The recovery can be estimated for each patient specifically by entering the different patient characteristics.

## Discussion

This study is to our knowledge the first that used secondary data to study the recovery of HRQL in burn patients. The 24-month recovery model shows the estimated course of improvement of HRQL over time in an average burn patient. The model particularly informs clinical practice regarding speed of recovery. One may expect the largest improvement within 6 months after burns, with slight further improvement until 18 months. Recovery for females, patients with a longer hospital stay and more severely burned patients was less favorable regarding speed and endpoint. Large improvements over time in the different dimensions of the EQ-5D were also seen, except for the dimensions anxiety/depression and pain/discomfort. Even up to 7 year post burn, these dimensions remained more impaired in burn patients compared to the general population, both in the group of patients with mild and intermediate burns as in the group of patients with major burns [37]. Also, problems with usual activities remained more prevalent in the group of patients with major burns, but not in the group of patients with mild and intermediate burns.

The 24-month recovery model can be used to inform patients on expected outcomes and to monitor HRQL outcomes in adults after burn injury [38]. In this way, it allows clinicians to identify deviation from the expected recovery of HRQL. Up to now, longer-term models only exist for young burned adults (19–30 years old) and burned children [6–8]. HRQL outcomes in children also strongly improve in the first six months post burn; however, in contrast to adults, outcomes in children did not stabilize over time due to the growth and continuing development of children. The burn specific HRQL model for young adults also showed major improvements in most domains in the six months following the injury, however for all except one domain (motor function), norm scores of the general population were not reached. This is comparable to our results; large improvement was seen in the six months following the injury; however, the norm score was not reached for an average patient at 24 months post injury.

The recovery of HRQL was found to be influenced by %TBSA burned; patients with a higher %TBSA burned had a delayed recovery and a lower HRQL as endpoint. The effect of %TBSA burned on HRQL recovery was also investigated by the studies in young adults and children [8, 39]. The results in children were similar to our results; the recovery of children with <20% TBSA burned was significantly better on all domains except for appearance compared to children with a TBSA burned of ≥20% [8]. However, the study in young adults showed inconsistent results; no clear differences were seen between the different groups based on %TBSA burned. This may be due to the relatively low number of patients studied.

Also, female gender and longer LOS were found to influence the recovery of HRQL in a less favorable way regarding speed and final score. There are some indications that outcomes differ between males and females, and between those with a short and long hospital stay. With respect to gender, a study by Wasiak et al. showed that female burn patients reported a poorer generic and burn-specific HRQL at 12 months after the injury [40] and a recent systematic review showed that the majority of studies found that female gender was a predictive factor of a diminished HRQL post-burn [41]. This is in line with other trauma populations and with general population outcomes [42–45]. Females in general seem to experience more problems, or are more willing to report health problems. And a changed appearance might have a greater impact on a females life [46]. Studies focusing on other outcomes in burns found that female burn patients had higher levels of fatigue, a worse opinion about their scars, more difficulties with social participation, and mortality rates are higher than in male patients [46–49]. With respect to longer LOS, many studies have shown that patients with a prolonged hospital stay have a diminished HRQL, both shortly and longer after burns [10, 41]. This is also in line with results from the general trauma population, where LOS has also been found to influence HRQL after injury [50, 51].

The several dimensions included in the EQ-5D showed a different recovery pattern. Up to 7 years post-burn, the proportion of burn patients experiencing problems with pain/discomfort and anxiety/depression remained higher than the proportion in the general population in both studied patient groups. Also, problems with usual activities remained more prevalent in the group of patients with major burns, but not in the group of patients with mild and intermediate burns. The problem of continued pain and discomfort is also described by other burn studies and is also present in other trauma populations [52–54]. Earlier studies also described the discrepancy between physical and psychological outcomes and their recovery over time. Psychological dimensions, like anxiety/depression, have a delayed and less progressive recovery than physical dimensions [8, 9, 24, 55]; indicating that a burn injury has the potency to elicit long-term psychological problems. These dimensions require special attention in the aftermath of burns in order to further improve HRQL after burns. Besides, patients with major burns might benefit from extra attention for usual activities.

This study also shows the benefits of the use of secondary data within the field of burns. By sharing data, we were able to develop a HRQL model up to 24 months after burns. The main advantage of this approach was that the large number of included patients increased statistical power which allows us to come to a reliable 24-month recovery model. However, combining different datasets and analyzing the combined dataset was also challenging. The different studies measured HRQL at different time points, had different study populations, measured HRQL in different ways and covered different timeframes. Improved burn care, burn treatment and scar therapy over time might have resulted in improved quality of care and possibly improved quality of life of burn survivors over the past decade. However, there is no evidence on this potential association. Transformation of HRQL data was needed in order to combine the different datasets, therefore we applied the algorithm of Gray et al. [21] to map the SF-12 to the EQ-5D-3L and the algorithm of van Hout et al. [23] to map the EQ-5D-5L to the 3L version. An earlier study showed that the quality of these estimated scores based on the algorithm of Gray et al. ranged from moderate to good for the different dimensions [56], though this algorithm is the only one estimating EQ-5D dimension scores from SF-12 scores. Except for the dimension pain/discomfort, all estimated dimension scores reported less problems compared to directly assessed EQ-5D outcomes [56]. In total, 33% of all of our outcomes were mapped by this algorithm. The mapping of the algorithm by van Hout et al. also resulted in slightly better outcomes (less problems). Only 7% of all outcomes were mapped by this algorithm, besides, these were all long-term (≥5 year) outcomes and thus not included in the recovery model. So, mapping might have resulted in slightly better outcomes than originally reported and thereby the 24-month recovery model might be slightly overestimating the recovery of HRQL. Complex statistical methods were needed that adjusted for the differences in case-mix, the different time points used in the different studies, and the different number of measurements for each patient. Due to the large differences in all these aspects, a model up to 24 months could be estimated as too few studies provided longer-term information. Another limitation is that the variables of the model were limited to age, gender, %TBSA, LOS and time since burn as these were the only variables available in all datasets. Another study showed, for example, that number of surgeries might also be an important factor in the recovery of HRQL [9]. Guidelines for systematic outcome registration might overcome some of the problems described as longitudinal outcomes are measured in the same way and on the same time points, and more patient and burn characteristics might be available. However, missing outcomes and drop-outs remain issues.

## Conclusion

The 24-month recovery model can be used in clinical practice to inform patients on expected HRQL outcomes and provide clinicians insights into the expected recovery of HRQL. In this way, a delayed recovery can be recognized in an early stage and timely interventions can be started in order to improve patient outcomes. However, external validation of the developed model is needed before implementation into clinical practice. Furthermore, our study showed the benefit of use of secondary data within the field of burns. Especially due to the small sample sizes in burns, secondary use of data should be considered when a research question is raised.

## Supporting information

S1 Appendix Dataset. (PDF)

S2 Appendix The 24-month recovery of health-related quality of life (EQ-5D utility) model. (PDF)
