# Supplementary material for: Recovery of health-related quality of life after burn injuries: An individual participant data meta-analysis
Source: PLoS One. 2020 Jan 10;15(1):e0226653. doi: 10.1371/journal.pone.0226653 (PMC6953837; doi:10.1371/journal.pone.0226653)
Supplement: S1 Appendix — (PDF) [file pone.0226653.s001.pdf]

Study	Patient	Timepoint	EQ1	EQ2	EQ3	EQ4	EQ5	EQvas	EQindex	EQ_SF	Age	Gender	TBSA	LOS
1	256	T84	1	1	1	2	2	60	0.81756	EQ	28	1	31.5	28
1	255	T84	1	1	1	2	1	70	0.88718	EQ	22	0	16	37
1	254	T84	2	1	1	2	1	70	0.85173	EQ	55	1	11	40
1	253	T84	1	1	1	1	1	95	1	EQ	40	1	16.1	31
1	252	T84	1	1	1	1	1	82	1	EQ	25	0	13	32
1	251	T84	1	1	1	2	2	70	0.81756	EQ	38	0	19	74
1	250	T84	1	1	1	1	1	97	1	EQ	33	1	30	20
1	249	T84	2	1	2	2	1	78	0.81257	EQ	52	0	45.5	61
1	248	T84	1	1	3	2	1	80	0.80042	EQ	65	0	7	48
1	246	T84	1	1	1	1	2	90	0.88345	EQ	65	0	8	33
1	245	T84	1	1	1	1	1	100	1	EQ	25	0	21	40
1	244	T84	1	1	1	1	2	79	0.88345	EQ	56	1	21	23
1	243	T84	2	1	1	2	1	80	0.85173	EQ	59	1	11	12
1	242	T84	1	1	2	2	1	85	0.84803	EQ	23	0	5.5	4
1	241	T84	3	1	2	2	1	86	0.79143	EQ	55	0	16.6	72
1	240	T84	1	1	1	2	1	99	0.88718	EQ	24	1	8	18
1	239	T84	3	2	3	3	4	69	0.32326	EQ	61	1	35	52
1	238	T84	2	1	1	1	2	70	0.848	EQ	47	0	13.5	25
1	237	T84	1	1	1	2	1	80	0.88718	EQ	39	0	19.5	27
1	236	T84	1	3	3	2	5	40	0.31925	EQ	22	1	28.5	70
1	235	T72	2	1	1	2	2	60	0.7821	EQ	39	1	37.5	45
1	234	T72	1	1	1	2	1	90	0.88718	EQ	27	1	40	42
1	233	T72	2	2	2	2	1	75	0.77446	EQ	58	1	34	33
1	232	T72	3	1	3	3	1	70	0.71776	EQ	40	1	67	89
1	231	T72	1	1	1	2	1	85	0.88718	EQ	45	1	42.3	86
1	230	T72	1	1	1	1	1	70	1	EQ	53	0	16	24
1	229	T72	1	1	3	2	1	80	0.80042	EQ	26	1	35	43
1	228	T72	1	1	1	1	1	100	1	EQ	37	1	5.5	16
1	227	T72	1	1	1	2	1	76	0.88718	EQ	41	0	8	14
1	226	T72	1	1	1	1	2	70	0.88345	EQ	33	0	26	32
1	225	T72	1	1	1	1	2	90	0.88345	EQ	52	0	9	16
1	224	T72	2	2	3	2	2	80	0.65724	EQ	30	1	10	78
1	223	T72	1	1	1	3	3	100	0.71659	EQ	31	0	7	20
1	222	T72	1	1	2	1	2	50	0.8443	EQ	43	0	6.5	33
1	221	T72	1	1	1	2	2	90	0.81756	EQ	43	1	34	54
1	220	T72	1	1	1	1	1	80	1	EQ	47	1	1	5
1	219	T72	1	1	1	1	1	90	1	EQ	54	1	4	11
1	218	T72	1	1	1	1	1	94	1	EQ	49	1	3.5	2
1	217	T72	2	1	3	1	2	65	0.76124	EQ	53	1	11.1	23
1	216	T72	1	1	2	1	1	90	0.91392	EQ	65	1	12	24
1	215	T72	1	1	1	1	1	100	1	EQ	16	1	30.5	42
1	214	T72	1	3	1	2	2	70	0.75702	EQ	59	0	15	50
1	213	T72	2	1	2	3	2	70	0.71688	EQ	67	0	14	28
1	212	T72	1	1	1	1	1	100	1	EQ	21	1	6.5	14
1	211	T72	1	1	1	1	1	95	1	EQ	46	1	4.6	2
1	210	T72	1	1	1	1	1	62	1	EQ	44	1	0.5	0
1	209	T72	4	3	3	1	2	78	0.57016	EQ	77	1	18	38
1	208	T72	4	3	4	3	4	50	0.08572	EQ	36	1	22.5	57
1	207	T72	2	1	2	2	4	50	0.45618	EQ	24	1	45	143
1	206	T72	1	1	1	1	1	85	1	EQ	55	1	0.5	0

1	205	T72	1	3	3	3	3	60	0.56929	EQ	74	0	16	21
1	204	T72	1	1	1	1	1	100	1	EQ	22	1	0.5	0
1	203	T72	1	1	1	1	1	75	1	EQ	28	0	8.6	8
1	202	T72	1	1	1	1	1	80	1	EQ	43	0	16.5	4
1	201	T72	1	1	1	1	1	80	1	EQ	71	0	1	8
1	200	T72	1	1	1	1	1	96	1	EQ	43	0	5.5	11
1	199	T72	1	1	1	1	1	95	1	EQ	15	0	16	10
1	198	T72	1	1	1	1	1	99	1	EQ	54	1	14.5	35
1	197	T72	5	2	2	4	1	70	0.31259	EQ	27	0	0.7	14
1	196	T72	1	1	1	2	1	99	0.88718	EQ	42	0	2	4
1	195	T72	1	1	1	1	1	100	1	EQ	72	1	0.1	0
1	194	T72	1	1	2	3	1	#N/B	0.82195	EQ	32	1	8.7	13
1	193	T72	1	1	1	1	1	100	1	EQ	35	0	6	11
1	192	T72	1	1	1	1	1	100	1	EQ	50	0	4.5	2
1	191	T72	1	1	1	1	1	95	1	EQ	61	1	2.5	3
1	190	T72	1	1	1	1	1	70	1	EQ	59	0	2.3	25
1	189	T72	1	1	1	2	2	80	0.81756	EQ	35	0	18	23
1	188	T72	1	1	2	2	2	70	0.7784	EQ	56	1	1	28
1	187	T72	1	1	1	1	2	88	0.88345	EQ	44	1	0.1	0
1	186	T72	1	1	1	1	1	90	1	EQ	22	1	0	1
1	185	T72	1	1	1	2	3	90	0.74266	EQ	35	1	2.5	15
1	184	T72	1	1	1	1	1	96	1	EQ	23	1	3.5	2
1	183	T72	1	1	1	1	1	80	1	EQ	21	1	1	4
1	182	T72	1	1	1	1	2	90	0.88345	EQ	17	0	4	1
1	181	T72	1	1	1	1	1	100	1	EQ	48	0	4.5	13
1	180	T72	1	1	2	2	2	65	0.7784	EQ	51	1	0.3	1
1	179	T72	1	1	1	1	4	70	0.59669	EQ	41	0	1	2
1	178	T72	3	2	3	1	2	45	0.70199	EQ	83	0	4	19
1	177	T72	1	1	1	1	1	70	1	EQ	52	1	6	10
1	176	T72	3	1	3	2	1	80	0.74383	EQ	23	1	13	15
1	175	T72	3	2	2	2	2	70	0.6837	EQ	53	1	0.5	0
1	174	T72	1	1	2	2	2	70	0.7784	EQ	76	0	4.9	37
1	173	T72	1	1	1	1	1	100	1	EQ	29	1	3.5	1
1	172	T72	1	1	1	1	1	80	1	EQ	70	0	25	24
1	171	T72	1	1	1	1	1	100	1	EQ	42	0	0.5	1
1	170	T72	1	1	1	1	1	100	1	EQ	31	1	6.5	7
1	169	T72	1	1	1	1	1	80	1	EQ	38	0	6	8
1	168	T72	1	1	1	2	1	85	0.88718	EQ	24	1	1	1
1	167	T72	1	1	1	1	1	100	1	EQ	68	1	5	1
1	166	T72	1	1	1	1	1	90	1	EQ	60	1	0.8	1
1	165	T72	1	1	1	1	1	95	1	EQ	35	1	0.2	0
1	164	T72	1	1	1	1	1	85	1	EQ	39	0	0.5	0
1	163	T72	1	1	1	1	1	60	1	EQ	72	0	3	3
1	162	T72	1	1	1	2	1	70	0.88718	EQ	68	0	12	17
1	161	T72	1	1	1	2	3	60	0.74266	EQ	25	0	15	21
1	160	T72	1	1	1	1	1	#N/B	1	EQ	19	1	1	1
1	159	T72	1	1	1	2	1	90	0.88718	EQ	32	1	5	2
1	158	T72	1	1	1	1	1	90	1	EQ	16	1	2	2
1	157	T72	1	1	1	2	1	97	0.88718	EQ	41	1	1.1	2
1	156	T72	2	1	1	2	2	78	0.7821	EQ	57	1	25	31
1	155	T72	1	1	3	3	2	85	0.70473	EQ	19	1	7.3	41

1	154	T72	1	1	1	1	1	98	1	EQ	57	0	13	35
1	153	T72	2	1	2	2	2	30	0.74295	EQ	30	1	6.5	2
1	152	T72	1	2	2	2	2	65	0.7403	EQ	53	1	1	1
1	151	T72	1	1	1	1	3	70	0.80855	EQ	25	1	0.4	2
1	150	T72	1	1	1	1	1	95	1	EQ	57	1	2	2
1	149	T72	1	1	1	2	3	70	0.74266	EQ	29	1	1	0
1	148	T72	1	1	1	1	2	75	0.88345	EQ	44	1	15	21
1	147	T72	1	1	3	2	3	55	0.6559	EQ	27	1	1.5	1
1	146	T72	1	1	1	1	1	100	1	EQ	57	1	1.5	14
1	145	T72	1	1	1	1	1	92	1	EQ	26	1	3	1
1	144	T72	1	1	1	1	1	70	1	EQ	34	1	3	8
1	143	T72	1	1	3	3	3	60	0.62983	EQ	48	0	61	69
1	142	T72	1	1	1	1	1	80	1	EQ	49	0	4.2	27
1	141	T72	1	1	1	1	2	80	0.88345	EQ	32	1	11	8
1	140	T72	1	1	3	2	1	80	0.80042	EQ	47	1	12	63
1	139	T72	1	1	1	1	1	90	1	EQ	53	1	1	3
1	138	T72	1	1	1	2	1	83	0.88718	EQ	29	0	1	0
1	137	T72	3	1	2	4	3	62	0.35287	EQ	18	1	6	6
1	136	T72	1	1	1	2	1	90	0.88718	EQ	65	0	3	27
1	135	T72	1	1	2	2	1	95	0.84803	EQ	51	1	75.5	69
1	134	T72	2	1	2	4	2	30	0.44892	EQ	55	0	0.5	1
1	133	T72	1	1	1	1	1	99	1	EQ	48	1	2	0
1	132	T72	1	1	1	1	1	90	1	EQ	28	1	8.2	10
1	131	T72	2	1	1	2	1	80	0.85173	EQ	64	1	1	9
1	130	T72	1	1	1	2	1	80	0.88718	EQ	49	1	1	1
1	129	T72	1	1	1	1	1	90	1	EQ	50	1	1	3
1	128	T72	1	1	1	1	1	90	1	EQ	16	0	2.5	2
1	127	T72	1	1	1	1	1	80	1	EQ	39	0	0.4	1
1	126	T60	1	1	1	1	1	100	1	EQ	48	0	1.4	14
1	125	T60	1	1	1	1	1	95	1	EQ	13	1	2	0
1	124	T60	1	1	1	1	1	85	1	EQ	25	1	1.5	1
1	123	T60	1	1	1	2	1	100	0.88718	EQ	19	1	3	15
1	122	T60	1	1	1	2	1	80	0.88718	EQ	39	1	14	13
1	121	T60	1	1	1	2	2	70	0.81756	EQ	72	1	2.6	16
1	120	T60	1	1	2	3	1	79	0.82195	EQ	32	0	5	20
1	119	T60	1	1	1	1	1	100	1	EQ	55	1	1	1
1	118	T60	1	1	1	1	1	95	1	EQ	32	1	6.6	25
1	117	T60	1	1	2	2	1	85	0.84803	EQ	23	0	9	6
1	116	T60	1	1	1	1	1	90	1	EQ	22	1	14	17
1	115	T60	2	1	2	3	3	64	0.64198	EQ	37	0	1	2
1	114	T60	1	1	1	1	1	70	1	EQ	55	1	9.5	11
1	113	T60	1	1	2	2	1	90	0.84803	EQ	22	1	1	8
1	112	T60	1	1	1	1	1	50	1	EQ	55	0	1	0
1	111	T60	1	1	1	1	3	100	0.80855	EQ	14	1	3	2
1	110	T60	1	1	1	1	1	100	1	EQ	63	0	6.5	26
1	109	T60	1	1	1	1	2	95	0.88345	EQ	41	0	9.5	25
1	108	T60	1	1	1	2	2	65	0.81756	EQ	66	0	2	0
1	107	T60	1	1	1	2	1	100	0.88718	EQ	36	1	6.6	16
1	105	T60	2	1	3	2	1	73	0.76497	EQ	24	1	7.7	26
1	104	T60	1	1	1	1	1	100	1	EQ	43	1	0.5	2
1	103	T60	1	1	1	1	1	90	1	EQ	59	1	4.5	8

1	102	T60	1	1	1	1	1	100	1	EQ	34	1	4	1
1	101	T60	3	1	1	4	2	29	0.46693	EQ	44	0	1	0
1	100	T60	1	1	1	1	1	90	1	EQ	53	1	1	0
1	99	T60	1	1	1	1	1	92	1	EQ	28	1	18	1
1	98	T60	1	1	1	1	1	100	1	EQ	21	1	0.5	1
1	97	T60	1	1	1	1	1	90	1	EQ	52	1	4	17
1	96	T60	1	1	1	2	2	85	0.81756	EQ	16	0	0.5	2
1	95	T60	1	1	1	1	1	80	1	EQ	42	1	3	1
1	94	T60	1	1	1	1	1	95	1	EQ	27	1	3.5	1
1	93	T60	1	1	1	1	2	80	0.88345	EQ	56	0	0.5	31
1	92	T60	1	1	1	2	3	80	0.74266	EQ	37	1	7	9
1	91	T60	1	1	1	1	1	88	1	EQ	59	1	0.7	5
1	90	T60	1	1	1	1	1	80	1	EQ	44	0	2	3
1	89	T60	3	1	3	1	3	68	0.6652	EQ	53	0	8	21
1	88	T60	1	2	3	3	3	76	0.59172	EQ	51	0	10.1	19
1	87	T60	1	1	1	1	1	90	1	EQ	37	1	9.2	23
1	86	T60	1	1	1	1	1	94	1	EQ	25	1	0.5	7
1	85	T60	1	1	1	1	2	100	0.88345	EQ	20	1	0.5	1
1	84	T60	1	1	1	1	1	100	1	EQ	30	0	3.5	5
1	83	T60	1	1	1	1	1	80	1	EQ	44	1	10.4	27
1	82	T60	1	1	1	1	1	90	1	EQ	24	1	4	7
1	81	T60	4	1	2	2	1	70	0.68202	EQ	74	1	14	28
1	80	T60	1	1	1	1	1	80	1	EQ	60	0	3	13
1	79	T60	1	1	1	1	2	85	0.88345	EQ	70	1	12	40
1	78	T60	1	1	1	1	1	100	1	EQ	43	1	4.5	5
1	77	T60	1	1	1	2	1	60	0.88718	EQ	31	1	2	21
1	76	T60	5	2	1	1	1	80	0.71167	EQ	15	0	1	0
1	75	T60	1	1	1	1	1	100	1	EQ	13	0	1.7	9
1	74	T60	1	1	1	2	1	90	0.88718	EQ	32	0	5.5	21
1	73	T60	1	1	1	1	1	86	1	EQ	46	1	34	7
1	72	T60	1	1	1	1	1	70	1	EQ	33	1	1.5	8
1	71	T60	1	1	1	3	1	100	0.86111	EQ	55	1	11	23
1	70	T60	1	1	3	3	1	80	0.77435	EQ	32	1	38	1
1	69	T60	1	1	1	1	1	60	1	EQ	43	0	3.2	13
1	68	T60	1	1	1	1	1	91	1	EQ	41	1	1	2
1	67	T60	3	1	1	3	4	60	0.44812	EQ	66	1	8	21
1	66	T60	1	1	1	1	1	90	1	EQ	25	0	11	14
1	65	T60	1	1	1	1	1	90	1	EQ	47	1	13	16
1	64	T60	1	1	1	1	1	100	1	EQ	35	0	1	5
1	63	T60	1	1	1	1	1	93	1	EQ	46	1	2.5	1
1	62	T60	1	1	1	2	1	70	0.88718	EQ	47	0	1	5
1	61	T60	1	1	1	1	1	100	1	EQ	60	1	1	13
1	60	T60	1	1	1	1	2	80	0.88345	EQ	24	0	13.5	23
1	59	T60	1	1	1	2	2	#N/B	0.81756	EQ	55	0	26	46
1	58	T60	1	1	1	1	4	30	0.59669	EQ	30	0	0.5	0
1	57	T60	1	1	1	2	1	80	0.88718	EQ	60	0	1	7
1	56	T60	1	1	1	1	1	100	1	EQ	19	1	6	6
1	55	T60	1	1	1	1	1	90	1	EQ	65	1	3.1	6
1	54	T60	1	1	1	1	1	100	1	EQ	36	1	3	6
1	53	T60	1	1	1	1	2	70	0.88345	EQ	67	0	7	20
1	52	T60	1	1	1	1	1	90	1	EQ	26	0	2	1

1	51	T60	1	1	1	1	1	90	1	EQ	16	1	8	7
1	50	T60	1	1	1	1	1	84	1	EQ	21	1	3	1
1	49	T60	1	1	1	1	1	90	1	EQ	28	1	36	10
1	48	T60	1	1	1	1	1	100	1	EQ	25	1	0	1
1	47	T60	1	1	1	1	1	75	1	EQ	70	1	20	29
1	46	T60	1	1	2	3	1	80	0.82195	EQ	53	1	16	16
1	45	T60	2	1	2	2	1	68	0.81257	EQ	59	0	2.5	20
1	44	T60	1	1	1	1	1	90	1	EQ	66	1	6.3	7
1	43	T60	1	1	1	2	1	100	0.88718	EQ	25	1	12	10
1	42	T60	1	1	1	1	1	100	1	EQ	71	1	3.5	10
1	41	T60	1	1	1	1	1	100	1	EQ	24	1	5	8
1	40	T60	1	1	1	1	1	90	1	EQ	69	1	1	1
1	39	T60	1	1	3	3	2	70	0.70473	EQ	42	1	6.1	27
1	38	T60	1	1	2	1	1	80	0.91392	EQ	50	1	7	12
1	37	T60	1	1	1	1	1	85	1	EQ	32	1	10	7
1	36	T60	2	2	2	2	2	90	0.70484	EQ	29	1	0.5	6
1	35	T60	1	1	1	1	1	100	1	EQ	30	1	6	1
1	34	T60	1	1	1	2	2	90	0.81756	EQ	46	0	6	8
1	33	T60	1	1	1	2	5	40	0.46654	EQ	33	0	6	0
1	32	T60	1	1	1	1	1	100	1	EQ	61	1	3	5
1	31	T60	1	1	1	1	2	80	0.88345	EQ	15	0	12	18
1	30	T60	1	1	2	3	1	77	0.82195	EQ	62	1	4.5	21
1	29	T60	1	1	1	1	2	94	0.88345	EQ	16	0	10	22
1	28	T60	2	1	2	3	1	82	0.7865	EQ	71	1	8	50
1	27	T60	1	2	3	3	4	50	0.37985	EQ	47	1	8.1	16
1	26	T60	1	1	1	1	1	90	1	EQ	48	1	4	4
1	25	T60	1	1	1	1	1	76	1	EQ	35	1	1.6	2
1	24	T60	1	1	1	1	2	80	0.88345	EQ	14	0	6	1
1	23	T60	1	1	1	2	1	100	0.88718	EQ	14	0	4	9
1	22	T60	1	1	1	2	2	100	0.81756	EQ	35	0	8	2
1	21	T60	1	1	1	1	4	72	0.59669	EQ	44	1	25.5	23
1	20	T60	1	1	2	2	1	90	0.84803	EQ	39	1	52	16
1	19	T60	1	1	1	1	2	79	0.88345	EQ	18	0	2	1
1	18	T60	1	1	1	1	1	50	1	EQ	62	1	0.5	2
1	17	T60	1	1	1	1	1	100	1	EQ	18	1	2.9	14
1	16	T60	1	1	2	2	2	60	0.7784	EQ	31	1	13.5	26
1	15	T60	1	1	1	1	2	76	0.88345	EQ	16	0	6.1	10
1	14	T60	1	1	1	1	1	85	1	EQ	20	0	6	3
1	13	T60	1	1	1	1	1	75	1	EQ	54	1	14	17
1	12	T60	1	1	1	1	1	83	1	EQ	69	1	2	15
1	11	T60	4	3	5	3	5	60	0.02147	EQ	77	0	8	24
1	10	T60	1	1	1	1	1	83	1	EQ	47	0	1	0
1	9	T60	3	2	3	1	1	45	0.77162	EQ	55	1	3	40
1	8	T60	1	1	1	1	1	90	1	EQ	22	0	18	47
1	7	T60	3	2	3	4	3	51	0.26716	EQ	54	1	53.5	89
1	6	T60	1	1	1	1	1	96	1	EQ	50	1	5	6
1	5	T60	1	1	1	1	1	100	1	EQ	36	0	2	1
1	4	T60	2	1	1	2	1	95	0.85173	EQ	56	0	26	70
1	3	T60	4	3	4	3	5	50	0.02147	EQ	62	1	48	161
1	2	T60	1	1	1	1	2	75	0.88345	EQ	55	1	6.5	9
1	1	T60	1	1	1	1	1	80	1	EQ	78	1	8	21

2	257	T12	1	1	1	1	1	70	1	EQ	51	1	25	18
2	257	T24	1	1	2	1	2	60	0.812	EQ	51	1	25	18
2	255	T3	1	1	2	1	2	75	0.812	EQ	46	1	9.5	15
2	252	T12	2	2	2	1	1	80	0.71	EQ	67	1	15	114
2	252	T24	2	2	1	1	1	100	0.746	EQ	67	1	15	114
2	248	T12	1	1	1	1	1	100	1	EQ	45	1	6	10
2	248	T24	1	1	1	1	1	93	1	EQ	45	1	6	10
2	248	T3	1	1	1	1	1	100	1	EQ	45	1	6	10
2	247	T12	1	1	1	2	2	90	0.725	EQ	24	0	8	54
2	247	T24	1	1	1	2	2	95	0.725	EQ	24	0	8	54
2	247	T3	1	2	3	2	2	50	0.258	EQ	24	0	8	54
2	245	T12	1	1	1	1	1	80	1	EQ	44	1	3	4
2	245	T24	1	1	1	1	1	80	1	EQ	44	1	3	4
2	245	T3	1	1	1	1	1	90	1	EQ	44	1	3	4
2	243	T12	1	1	1	1	1	100	1	EQ	45	0	6	8
2	243	T24	1	1	1	1	1	98	1	EQ	45	0	6	8
2	243	T3	1	1	1	1	2	90	0.848	EQ	45	0	6	8
2	241	T3	2	1	2	2	1	65	0.691	EQ	67	1	0.5	2
2	239	T12	1	1	1	1	1	80	1	EQ	44	0	1	0
2	239	T24	1	1	1	1	1	80	1	EQ	44	0	1	0
2	239	T3	2	1	2	3	1	40	0.159	EQ	44	0	1	0
2	236	T12	1	1	1	1	1	99	1	EQ	40	1	5	6
2	236	T24	1	1	1	1	2	95	0.848	EQ	40	1	5	6
2	235	T24	1	1	1	1	1	100	1	EQ	35	1	5	1
2	235	T3	1	1	1	1	1	95	1	EQ	35	1	5	1
2	234	T12	1	1	1	2	2	75	0.725	EQ	38	1	7	9
2	234	T24	1	1	1	1	1	85	1	EQ	38	1	7	9
2	231	T12	1	1	1	1	2	70	0.848	EQ	66	1	8	21
2	231	T24	2	1	1	2	2	60	0.656	EQ	66	1	8	21
2	231	T3	2	1	1	1	2	70	0.779	EQ	66	1	8	21
2	230	T12	1	1	1	1	1	80	1	EQ	53	0	5	50
2	230	T24	1	1	1	1	1	90	1	EQ	53	0	5	50
2	230	T3	2	1	2	1	1	75	0.814	EQ	53	0	5	50
2	229	T12	1	1	1	1	1	65	1	EQ	60	1	1	13
2	229	T24	1	1	1	1	1	#N/B	1	EQ	60	1	1	13
2	229	T3	1	1	1	1	1	80	1	EQ	60	1	1	13
2	228	T12	1	1	1	1	2	70	0.848	EQ	70	1	12	40
2	228	T24	1	1	1	1	2	75	0.848	EQ	70	1	12	40
2	228	T3	1	1	2	1	1	50	0.883	EQ	70	1	12	40
2	224	T12	1	1	1	1	1	90	1	EQ	41	1	1	2
2	224	T24	1	1	1	1	1	80	1	EQ	41	1	1	2
2	224	T3	1	1	1	1	1	90	1	EQ	41	1	1	2
2	222	T12	1	2	2	2	1	55	0.656	EQ	53	1	16	16
2	222	T24	1	1	2	2	1	80	0.76	EQ	53	1	16	16
2	222	T3	2	1	2	2	2	47	0.62	EQ	53	1	16	16
2	220	T12	1	1	1	2	1	85	0.796	EQ	55	1	11	23
2	220	T24	1	1	2	2	1	95	0.76	EQ	55	1	11	23
2	220	T3	1	1	1	1	1	95	1	EQ	55	1	11	23
2	219	T12	2	1	2	2	3	50	0.186	EQ	46	0	15	40
2	213	T12	2	1	1	1	1	80	0.85	EQ	88	1	8	26
2	213	T24	2	2	2	1	2	50	0.639	EQ	88	1	8	26

2	213	T3	2	1	1	1	1	90	0.85	EQ	88	1	8	26
2	209	T12	1	1	1	1	1	90	1	EQ	36	1	1.5	1
2	209	T3	1	1	1	2	1	60	0.796	EQ	36	1	1.5	1
2	207	T12	1	1	1	1	1	95	1	EQ	24	1	4	7
2	207	T3	1	1	1	2	1	90	0.796	EQ	24	1	4	7
2	205	T12	1	1	1	1	1	80	1	EQ	60	0	7	25
2	205	T24	1	1	1	1	1	75	1	EQ	60	0	7	25
2	205	T3	1	2	2	2	1	60	0.656	EQ	60	0	7	25
2	204	T3	1	1	2	2	2	60	0.689	EQ	27	0	8	23
2	200	T12	1	1	1	2	1	94	0.796	EQ	22	1	1	8
2	200	T24	1	1	1	2	1	94	0.796	EQ	22	1	1	8
2	196	T3	1	1	1	1	1	80	1	EQ	28	1	9	19
2	194	T12	1	1	1	1	1	90	1	EQ	25	1	2	7
2	194	T24	1	1	1	1	1	88	1	EQ	25	1	2	7
2	193	T3	2	1	2	2	2	60	0.62	EQ	48	0	1	5
2	192	T12	1	1	1	1	1	85	1	EQ	20	0	10	23
2	192	T24	1	1	1	1	1	100	1	EQ	20	0	10	23
2	192	T3	1	1	1	2	1	100	0.796	EQ	20	0	10	23
2	191	T12	1	1	1	1	1	85	1	EQ	54	0	18	13
2	191	T24	1	1	1	1	1	#N/B	1	EQ	54	0	18	13
2	191	T3	1	1	1	2	1	80	0.796	EQ	54	0	18	13
2	190	T12	1	1	1	1	1	100	1	EQ	22	1	10	4
2	190	T24	1	1	1	1	1	70	1	EQ	22	1	10	4
2	190	T3	1	1	1	1	1	100	1	EQ	22	1	10	4
2	186	T12	1	1	1	1	1	100	1	EQ	45	1	0.25	1
2	186	T24	1	1	1	1	1	100	1	EQ	45	1	0.25	1
2	184	T12	1	1	1	1	1	100	1	EQ	48	1	2	0
2	184	T24	1	1	1	1	1	90	1	EQ	48	1	2	0
2	184	T3	1	1	1	1	1	100	1	EQ	48	1	2	0
2	182	T12	1	1	1	1	1	90	1	EQ	24	1	2	0
2	182	T24	1	1	1	1	1	88	1	EQ	24	1	2	0
2	182	T3	1	1	1	1	1	95	1	EQ	24	1	2	0
2	181	T12	2	2	2	2	2	40	0.516	EQ	36	0	1	0
2	181	T24	2	1	2	2	2	30	0.62	EQ	36	0	1	0
2	181	T3	2	2	2	1	3	30	0.205	EQ	36	0	1	0
2	179	T12	1	2	2	1	2	60	0.708	EQ	55	1	12	88
2	179	T3	2	2	2	2	1	80	0.587	EQ	55	1	12	88
2	178	T12	1	1	2	2	2	65	0.689	EQ	21	1	26	59
2	178	T24	1	1	2	2	2	70	0.689	EQ	21	1	26	59
2	178	T3	1	2	3	2	2	60	0.258	EQ	21	1	26	59
2	177	T12	2	2	2	2	2	20	0.516	EQ	65	0	8	44
2	177	T24	2	2	2	2	1	75	0.587	EQ	65	0	8	44
2	177	T3	2	2	2	1	1	60	0.71	EQ	65	0	8	44
2	176	T12	1	1	1	1	1	90	1	EQ	24	1	6.5	34
2	176	T24	1	1	1	1	1	90	1	EQ	24	1	6.5	34
2	176	T3	2	1	2	1	1	78	0.814	EQ	24	1	6.5	34
2	174	T12	1	1	1	2	1	60	0.796	EQ	44	0	6.5	19
2	174	T24	1	1	2	2	1	75	0.76	EQ	44	0	6.5	19
2	174	T3	2	2	2	3	2	65	-0.016	EQ	44	0	6.5	19
2	173	T12	1	1	1	1	1	90	1	EQ	35	0	6	11
2	173	T24	1	1	1	1	1	85	1	EQ	35	0	6	11

2	173	T3	1	1	1	1	2	90	0.848	EQ	35	0	6	11
2	172	T12	1	1	1	1	1	77	1	EQ	55	1	9.5	11
2	172	T24	1	1	1	1	1	70	1	EQ	55	1	9.5	11
2	172	T3	1	1	1	1	2	72	0.848	EQ	55	1	9.5	11
2	169	T12	1	1	1	1	2	75	0.848	EQ	58	1	25	31
2	169	T24	1	1	1	1	2	90	0.848	EQ	58	1	25	31
2	169	T3	2	1	2	2	2	85	0.62	EQ	58	1	25	31
2	167	T12	1	1	1	1	1	95	1	EQ	23	1	14	17
2	167	T24	1	1	1	1	1	95	1	EQ	23	1	14	17
2	167	T3	1	1	2	1	1	95	0.883	EQ	23	1	14	17
2	166	T12	1	1	1	1	1	100	1	EQ	40	0	1	4
2	166	T24	1	1	1	1	1	100	1	EQ	40	0	1	4
2	161	T12	2	2	2	1	2	70	0.639	EQ	67	1	1	31
2	161	T24	2	3	2	2	2	50	0.137	EQ	67	1	1	31
2	161	T3	2	2	2	2	2	60	0.516	EQ	67	1	1	31
2	157	T12	1	1	1	1	1	100	1	EQ	19	1	1	1
2	156	T12	1	1	1	2	1	85	0.796	EQ	51	1	75.5	69
2	156	T24	1	1	1	2	1	90	0.796	EQ	51	1	75.5	69
2	156	T3	1	2	2	1	1	75	0.779	EQ	51	1	75.5	69
2	155	T12	1	1	1	2	1	65	0.796	EQ	44	0	36	40
2	155	T24	1	1	1	2	1	30	0.796	EQ	44	0	36	40
2	151	T12	2	1	1	2	2	50	0.656	EQ	63	1	10	40
2	151	T24	2	1	1	2	1	80	0.727	EQ	63	1	10	40
2	151	T3	1	1	1	2	2	40	0.725	EQ	63	1	10	40
2	149	T12	1	1	1	1	1	85	1	EQ	24	1	7	7
2	149	T24	1	1	1	1	1	90	1	EQ	24	1	7	7
2	149	T3	1	1	1	1	1	85	1	EQ	24	1	7	7
2	148	T12	1	1	1	2	1	100	0.796	EQ	22	1	3.5	2
2	148	T24	1	1	1	1	1	100	1	EQ	22	1	3.5	2
2	148	T3	1	1	2	1	1	90	0.883	EQ	22	1	3.5	2
2	146	T12	1	1	1	2	2	80	0.725	EQ	31	1	6.6	25
2	146	T24	1	1	1	1	1	92	1	EQ	31	1	6.6	25
2	146	T3	1	1	1	1	2	85	0.848	EQ	31	1	6.6	25
2	145	T12	1	2	2	2	1	50	0.656	EQ	38	0	1	2
2	145	T24	2	1	1	2	1	60	0.727	EQ	38	0	1	2
2	145	T3	1	1	1	2	1	60	0.796	EQ	38	0	1	2
2	141	T12	1	1	1	1	1	80	1	EQ	34	1	3	8
2	141	T24	1	1	1	1	1	#N/B	1	EQ	34	1	3	8
2	140	T12	1	1	1	1	1	85	1	EQ	59	1	4.5	8
2	140	T24	1	1	1	1	1	90	1	EQ	59	1	4.5	8
2	140	T3	1	1	1	1	1	90	1	EQ	59	1	4.5	8
2	139	T12	1	1	1	1	1	95	1	EQ	28	0	5	15
2	139	T24	1	1	1	1	1	95	1	EQ	28	0	5	15
2	139	T3	1	1	1	2	1	88	0.796	EQ	28	0	5	15
2	137	T12	1	1	3	3	3	10	-0.066	EQ	32	1	3	8
2	137	T24	1	1	3	3	3	25	-0.066	EQ	32	1	3	8
2	137	T3	1	1	2	3	3	40	-0.008	EQ	32	1	3	8
2	135	T12	1	1	1	1	1	90	1	EQ	60	0	3	13
2	135	T24	1	1	1	1	1	85	1	EQ	60	0	3	13
2	135	T3	1	1	1	1	1	90	1	EQ	60	0	3	13
2	132	T3	2	1	1	2	1	80	0.727	EQ	66	0	10	19

2	129	T12	1	1	2	2	2	#N/B	0.689	EQ	60	1	3	11
2	129	T3	1	2	2	2	2	70	0.585	EQ	60	1	3	11
2	125	T12	1	1	1	1	1	100	1	EQ	28	1	8.2	10
2	125	T24	1	1	1	1	1	100	1	EQ	28	1	8.2	10
2	125	T3	1	1	1	1	1	90	1	EQ	28	1	8.2	10
2	121	T12	1	1	1	1	1	#N/B	1	EQ	64	1	0.5	0
2	121	T24	1	1	1	1	1	90	1	EQ	64	1	0.5	0
2	121	T3	1	1	1	1	1	#N/B	1	EQ	64	1	0.5	0
2	119	T3	2	3	3	2	1	60	0.15	EQ	83	0	6	34
2	117	T12	2	1	1	2	2	75	0.656	EQ	32	0	5	20
2	117	T24	1	1	1	2	1	60	0.796	EQ	32	0	5	20
2	117	T3	1	1	1	2	2	80	0.725	EQ	32	0	5	20
2	115	T12	1	2	2	2	1	#N/B	0.656	EQ	74	0	16	21
2	115	T24	1	2	2	1	2	68	0.708	EQ	74	0	16	21
2	115	T3	2	2	2	2	2	50	0.516	EQ	74	0	16	21
2	113	T3	3	2	3	2	2	63	-0.056	EQ	32	1	90	57
2	112	T24	1	1	1	1	1	90	1	EQ	36	1	1	1
2	111	T12	1	1	1	2	1	80	0.796	EQ	33	1	18	21
2	111	T24	2	2	3	3	2	20	-0.074	EQ	33	1	18	21
2	111	T3	2	1	2	2	2	70	0.62	EQ	33	1	18	21
2	110	T12	1	1	1	2	1	70	0.796	EQ	68	1	8.5	27
2	107	T24	1	1	1	1	2	70	0.848	EQ	51	0	1.5	6
2	107	T3	1	1	1	1	2	85	0.848	EQ	51	0	1.5	6
2	105	T24	1	2	2	2	2	40	0.585	EQ	29	1	6.5	2
2	101	T24	1	1	1	2	2	40	0.725	EQ	54	1	1	1
2	97	T12	2	1	1	1	1	75	0.85	EQ	34	1	17	68
2	97	T3	2	1	2	1	1	70	0.814	EQ	34	1	17	68
2	93	T12	1	1	1	2	2	55	0.725	EQ	25	1	0.4	2
2	93	T24	1	1	1	1	1	90	1	EQ	25	1	0.4	2
2	93	T3	1	1	1	1	1	95	1	EQ	25	1	0.4	2
2	85	T24	1	1	1	1	1	70	1	EQ	40	1	1	1
2	85	T3	1	1	1	1	1	75	1	EQ	40	1	1	1
2	84	T12	1	1	1	2	1	80	0.796	EQ	65	0	3	27
2	84	T24	1	1	1	1	1	80	1	EQ	65	0	3	27
2	84	T3	1	1	1	2	1	80	0.796	EQ	65	0	3	27
2	81	T24	1	1	1	1	1	90	1	EQ	42	0	0.5	1
2	81	T3	1	1	1	1	1	100	1	EQ	42	0	0.5	1
2	73	T12	1	1	1	1	1	95	1	EQ	23	0	7	13
2	73	T24	1	1	1	2	1	100	0.796	EQ	23	0	7	13
2	73	T3	1	1	1	1	1	95	1	EQ	23	0	7	13
2	72	T12	2	1	1	2	1	80	0.727	EQ	63	1	1	9
2	72	T24	2	1	1	2	1	90	0.727	EQ	63	1	1	9
2	72	T3	1	1	1	1	1	90	1	EQ	63	1	1	9
2	66	T12	1	1	1	1	1	100	1	EQ	31	1	0.5	1
2	66	T3	1	1	1	1	1	100	1	EQ	31	1	0.5	1
2	63	T12	1	1	1	1	1	100	1	EQ	43	1	14	29
2	63	T24	1	1	1	1	1	100	1	EQ	43	1	14	29
2	63	T3	1	1	1	1	1	100	1	EQ	43	1	14	29
2	62	T24	1	1	1	1	2	70	0.848	EQ	32	1	11	8
2	60	T12	1	1	1	1	1	30	1	EQ	72	0	3	3
2	60	T24	2	1	2	2	1	80	0.691	EQ	72	0	3	3

2	60	T3	1	2	2	1	1	80	0.779	EQ	72	0	3	3
2	58	T24	1	1	1	1	1	80	1	EQ	54	1	3	1
2	56	T12	1	1	1	2	3	62	0.291	EQ	18	1	6	6
2	56	T24	1	1	2	1	2	40	0.812	EQ	18	1	6	6
2	56	T3	1	1	2	2	3	38	0.255	EQ	18	1	6	6
2	54	T12	1	1	1	1	1	100	1	EQ	20	1	3	2
2	52	T12	1	1	1	1	1	70	1	EQ	61	1	2.5	3
2	52	T24	1	1	1	1	1	80	1	EQ	61	1	2.5	3
2	52	T3	1	1	1	1	1	95	1	EQ	61	1	2.5	3
2	50	T3	3	3	2	2	2	#N/B	-0.108	EQ	41	1	20	53
2	45	T12	1	1	2	2	1	70	0.76	EQ	47	1	12	63
2	45	T24	1	1	2	2	1	70	0.76	EQ	47	1	12	63
2	45	T3	1	1	1	1	1	80	1	EQ	47	1	12	63
2	39	T12	1	1	1	1	1	95	1	EQ	50	1	1	3
2	39	T24	1	1	1	1	1	100	1	EQ	50	1	1	3
2	39	T3	1	1	1	1	1	100	1	EQ	50	1	1	3
2	38	T12	1	1	1	1	1	95	1	EQ	65	1	12	24
2	38	T24	1	1	1	1	1	97	1	EQ	65	1	12	24
2	38	T3	1	2	2	1	1	85	0.779	EQ	65	1	12	24
2	34	T12	2	1	2	2	1	70	0.691	EQ	83	0	4	19
2	34	T24	2	2	2	2	2	50	0.516	EQ	83	0	4	19
2	34	T3	2	2	2	2	1	65	0.587	EQ	83	0	4	19
2	33	T12	1	1	1	1	1	85	1	EQ	51	1	1	2
2	33	T24	1	1	1	1	1	98	1	EQ	51	1	1	2
2	30	T24	1	1	1	1	1	85	1	EQ	20	1	5	15
2	30	T3	1	1	1	1	1	90	1	EQ	20	1	5	15
2	28	T24	1	1	2	1	1	70	0.883	EQ	18	1	32	57
2	28	T3	1	2	1	2	1	80	0.692	EQ	18	1	32	57
2	27	T12	1	1	1	1	1	95	1	EQ	25	0	20	26
2	27	T24	1	1	1	1	1	95	1	EQ	25	0	20	26
2	25	T3	1	1	1	1	1	100	1	EQ	23	1	13	15
2	21	T3	1	1	1	2	2	80	0.725	EQ	23	0	6.5	8
2	18	T12	1	1	1	1	1	100	1	EQ	53	1	1	1
2	18	T24	1	1	1	1	1	100	1	EQ	53	1	1	1
2	18	T3	1	1	1	2	2	80	0.725	EQ	53	1	1	1
2	17	T3	2	1	3	3	2	50	0.03	EQ	28	0	12.5	178
2	9	T12	1	1	2	2	2	30	0.689	EQ	27	1	1.5	1
2	9	T24	1	1	2	2	2	60	0.689	EQ	27	1	1.5	1
2	9	T3	1	1	2	2	2	65	0.689	EQ	27	1	1.5	1
2	8	T12	1	1	1	2	1	100	0.796	EQ	37	1	5.5	16
2	8	T24	1	1	1	1	1	100	1	EQ	37	1	5.5	16
2	8	T3	1	1	1	1	1	100	1	EQ	37	1	5.5	16
2	5	T12	1	1	1	1	1	100	1	EQ	35	1	2.5	15
2	5	T24	1	1	1	1	2	70	0.848	EQ	35	1	2.5	15
2	5	T3	1	1	1	1	1	60	1	EQ	35	1	2.5	15
3	90	T84	1	1	1	1	1	#N/B	1	SF	26	0	4	3
3	89	T84	1	1	1	1	1	#N/B	1	SF	53	1	0	1
3	88	T84	1	1	2	2	2	#N/B	0.689	SF	46	0	4	20
3	87	T84	2	1	1	2	1	#N/B	0.727	SF	35	1	28.5	22
3	86	T84	1	1	1	1	2	#N/B	0.848	SF	48	1	8.5	14
3	85	T84	2	1	2	2	1	#N/B	0.691	SF	44	1	10	26

3	84	T84	2	1	2	2	1	#N/B	0.691	SF	55	0	45	61
3	83	T84	1	1	1	1	1	#N/B	1	SF	28	1	4	41
3	82	T84	1	1	1	1	1	#N/B	1	SF	34	1	30	52
3	81	T84	1	1	1	1	1	#N/B	1	SF	68	1	8	14
3	80	T72	1	1	1	1	1	#N/B	1	SF	62	1	12	18
3	79	T72	1	1	1	1	1	#N/B	1	SF	59	1	3	1
3	78	T72	1	1	2	2	3	#N/B	0.255	SF	41	1	10	16
3	77	T72	1	1	1	1	1	#N/B	1	SF	47	1	38	22
3	76	T72	1	1	1	1	1	#N/B	1	SF	25	0	25	26
3	75	T72	2	1	2	2	1	#N/B	0.691	SF	33	1	13.5	6
3	74	T72	1	1	1	1	1	#N/B	1	SF	31	1	15	15
3	73	T72	2	2	3	2	2	#N/B	0.189	SF	54	1	11	28
3	72	T72	2	1	2	1	2	#N/B	0.743	SF	30	1	35	20
3	71	T72	2	1	2	2	2	#N/B	0.62	SF	43	1	20	8
3	70	T72	1	1	1	1	1	#N/B	1	SF	51	1	17	19
3	69	T72	1	1	1	1	1	#N/B	1	SF	53	0	50	43
3	68	T72	1	1	1	2	1	#N/B	0.796	SF	52	1	6.5	2
3	67	T72	1	1	1	2	1	#N/B	0.796	SF	44	1	6.5	2
3	66	T72	1	1	1	1	1	#N/B	1	SF	49	1	2	6
3	65	T72	2	1	2	2	2	#N/B	0.62	SF	50	0	29.5	51
3	64	T72	1	1	1	1	1	#N/B	1	SF	46	1	12	11
3	63	T60	1	1	1	1	1	#N/B	1	SF	25	1	37	13
3	62	T60	1	1	1	1	1	#N/B	1	SF	46	1	15	11
3	61	T60	1	1	1	1	1	#N/B	1	SF	38	1	36	13
3	60	T60	1	1	1	1	1	#N/B	1	SF	51	1	11	3
3	59	T60	1	1	1	2	1	#N/B	0.796	SF	36	1	23	22
3	58	T60	1	1	1	1	1	#N/B	1	SF	22	1	37	20
3	57	T60	1	1	1	1	1	#N/B	1	SF	54	1	40	112
3	56	T60	1	1	1	1	1	#N/B	1	SF	25	1	12	14
3	55	T60	1	1	1	1	1	#N/B	1	SF	47	1	2.5	3
3	54	T60	1	1	1	1	1	#N/B	1	SF	34	1	4	2
3	53	T60	2	1	2	2	1	#N/B	0.691	SF	39	1	5	20
3	52	T48	1	1	1	1	1	#N/B	1	SF	45	1	33	31
3	51	T48	1	1	1	1	1	#N/B	1	SF	36	1	8	12
3	50	T48	1	1	1	1	1	#N/B	1	SF	41	1	36	56
3	49	T48	1	1	1	2	2	#N/B	0.725	SF	48	1	9	15
3	48	T48	1	1	2	2	2	#N/B	0.689	SF	31	1	22	42
3	47	T48	1	1	1	1	1	#N/B	1	SF	36	1	5	9
3	46	T48	1	1	1	1	1	#N/B	1	SF	35	1	19.5	12
3	45	T48	2	1	2	2	2	#N/B	0.62	SF	55	1	40	49
3	44	T48	1	1	1	1	1	#N/B	1	SF	31	1	11	14
3	43	T48	2	1	2	2	3	#N/B	0.186	SF	41	1	18.5	54
3	42	T48	1	1	1	2	1	#N/B	0.796	SF	39	0	10.5	48
3	41	T48	1	1	1	1	1	#N/B	1	SF	83	1	15	43
3	40	T48	2	2	2	2	2	#N/B	0.516	SF	80	0	5	30
3	39	T48	2	1	2	2	1	#N/B	0.691	SF	40	0	20	9
3	38	T48	2	1	2	1	2	#N/B	0.743	SF	37	1	50	55
3	37	T36	1	1	1	3	3	#N/B	0.028	SF	40	1	2	2
3	36	T36	1	1	1	1	1	#N/B	1	SF	54	1	9	1
3	35	T36	2	1	2	2	2	#N/B	0.62	SF	49	1	29	11
3	34	T36	1	1	1	1	1	#N/B	1	SF	31	1	46.5	45

3	33	T36	1	1	1	2	1	#N/B	0.796	SF	58	1	2	15
3	32	T36	1	1	1	1	1	#N/B	1	SF	39	1	5	7
3	31	T36	2	1	2	3	1	#N/B	0.159	SF	54	1	17.5	30
3	30	T36	1	1	2	1	2	#N/B	0.812	SF	59	1	24	26
3	29	T36	2	1	2	2	1	#N/B	0.691	SF	64	0	15	2
3	28	T36	1	1	1	1	1	#N/B	1	SF	29	1	28	16
3	27	T24	1	1	1	2	2	#N/B	0.725	SF	27	1	9	6
3	26	T24	1	1	1	1	1	#N/B	1	SF	34	1	15	15
3	25	T24	1	1	1	1	1	#N/B	1	SF	23	1	15	18
3	24	T24	2	1	2	2	1	#N/B	0.691	SF	48	0	25	41
3	23	T24	1	1	2	2	2	#N/B	0.689	SF	67	1	25	55
3	22	T24	1	1	1	2	2	#N/B	0.725	SF	36	1	5	15
3	21	T24	1	1	1	2	2	#N/B	0.725	SF	35	1	3	1
3	20	T24	2	1	2	2	1	#N/B	0.691	SF	77	1	5	18
3	19	T24	2	1	1	2	1	#N/B	0.727	SF	76	0	7	15
3	18	T18	1	1	1	1	1	#N/B	1	SF	35	1	4	14
3	17	T18	1	1	1	1	1	#N/B	1	SF	56	1	43	62
3	16	T18	1	1	1	1	1	#N/B	1	SF	32	1	25	13
3	15	T18	1	1	1	2	1	#N/B	0.796	SF	29	1	12	83
3	14	T18	1	1	1	1	1	#N/B	1	SF	20	1	7	1
3	13	T18	1	1	1	1	1	#N/B	1	SF	21	1	22	13
3	12	T18	1	1	1	1	2	#N/B	0.848	SF	25	1	22.5	17
3	11	T18	1	1	1	2	1	#N/B	0.796	SF	25	1	12	5
3	10	T12	2	1	2	2	1	#N/B	0.691	SF	43	1	15	27
3	9	T12	1	1	2	2	1	#N/B	0.76	SF	32	1	6	22
3	8	T12	2	1	2	2	1	#N/B	0.691	SF	50	1	26.5	43
3	7	T12	2	1	1	2	2	#N/B	0.656	SF	59	0	26	41
3	6	T12	1	1	1	2	2	#N/B	0.725	SF	42	1	18	8
3	5	T12	1	1	1	2	1	#N/B	0.796	SF	29	1	20	17
3	4	T12	1	3	1	2	1	#N/B	0.313	SF	43	1	7	2
3	3	T12	1	2	1	2	1	#N/B	0.692	SF	20	0	28	53
3	2	T12	2	2	3	3	3	#N/B	-0.239	SF	65	0	10.5	7
3	1	T12	1	1	1	2	2	#N/B	0.725	SF	41	1	30	16
4	173	T12	1	1	1	2	2	70	0.725	EQ	47	0	19	13
4	172	T12	1	1	1	2	2	50	0.725	EQ	39	1	22	4
4	172	T24	1	1	1	2	2	70	0.725	EQ	39	1	22	4
4	171	T12	1	1	1	2	2	90	0.725	EQ	24	1	39	29
4	171	T24	1	1	1	2	2	80	0.725	EQ	24	1	39	29
4	170	T24	2	2	3	3	3	40	-0.239	EQ	48	1	17	22
4	169	T12	2	2	2	2	2	52	0.516	EQ	24	1	38	82
4	168	T12	1	1	1	1	1	90	1	EQ	43	1	12	4
4	167	T12	1	1	2	2	1	70	0.76	EQ	55	0	18.3	10
4	167	T24	1	1	1	2	1	85	0.796	EQ	55	0	18.3	10
4	166	T12	1	1	1	2	1	70	0.796	EQ	56	1	7	14
4	165	T12	1	1	2	2	2	70	0.689	EQ	53	0	28	18
4	165	T24	1	1	1	1	1	90	1	EQ	53	0	28	18
4	164	T12	1	1	1	1	1	90	1	EQ	74	1	9	17
4	164	T24	1	1	1	2	1	70	0.796	EQ	74	1	9	17
4	163	T12	1	1	1	1	1	90	1	EQ	80	1	16	24
4	163	T24	1	1	1	1	1	96	1	EQ	80	1	16	24
4	162	T12	1	1	2	1	2	70	0.812	EQ	47	1	8	7

4	162	T24	1	1	1	1	2	80	0.848	EQ	47	1	8	7
4	152	T12	1	1	1	1	3	90	0.414	EQ	40	1	10	7
4	152	T24	1	1	1	1	2	85	0.848	EQ	40	1	10	7
4	151	T12	1	1	2	2	2	70	0.689	EQ	39	0	49	126
4	151	T24	1	1	2	2	1	85	0.76	EQ	39	0	49	126
4	143	T12	2	1	1	2	1	70	0.727	EQ	21	1	55.8	65
4	143	T24	1	1	3	2	1	#N/B	0.433	EQ	21	1	55.8	65
4	140	T12	1	1	1	1	1	100	1	EQ	38	0	29	12
4	134	T12	2	2	3	2	3	#N/B	0.024	EQ	44	0	52.5	121
4	130	T12	2	2	2	2	2	40	0.516	EQ	83	1	30	56
4	123	T12	1	1	3	2	2	80	0.362	EQ	45	1	14	19
4	123	T24	1	1	1	1	2	#N/B	0.848	EQ	45	1	14	19
4	122	T12	1	1	1	2	2	70	0.725	EQ	48	1	48	50
4	122	T24	1	1	1	1	1	#N/B	1	EQ	48	1	48	50
4	120	T12	1	1	1	2	1	86	0.796	EQ	56	1	10.5	1
4	109	T12	1	2	1	2	1	50	0.692	EQ	64	1	3.8	12
4	109	T24	1	1	1	2	2	#N/B	0.725	EQ	64	1	3.8	12
4	108	T12	2	1	1	2	2	69	0.656	EQ	57	1	20.8	23
4	108	T24	1	1	1	2	2	#N/B	0.725	EQ	57	1	20.8	23
4	105	T12	1	1	1	1	2	90	0.848	EQ	36	1	10	2
4	95	T12	2	2	2	2	2	60	0.516	EQ	84	1	27.5	64
4	95	T24	2	2	2	2	2	#N/B	0.516	EQ	84	1	27.5	64
4	94	T12	1	1	3	1	3	30	0.32	EQ	42	1	19	22
4	94	T24	1	1	1	1	3	20	0.414	EQ	42	1	19	22
4	93	T12	2	2	2	2	1	80	0.587	EQ	29	1	52.8	234
4	93	T24	2	1	2	2	1	90	0.691	EQ	29	1	52.8	234
4	92	T12	2	1	2	2	3	30	0.186	EQ	54	1	47.85	42
4	91	T12	1	1	1	1	1	75	1	EQ	30	1	18.5	22
4	91	T24	1	1	1	2	2	80	0.725	EQ	30	1	18.5	22
4	90	T12	1	1	2	2	2	70	0.689	EQ	43	1	41.5	76
4	90	T24	1	1	1	2	2	70	0.725	EQ	43	1	41.5	76
4	89	T12	1	1	1	1	1	100	1	EQ	63	0	11.25	12
4	89	T24	1	1	1	1	1	100	1	EQ	63	0	11.25	12
4	88	T12	1	1	1	1	1	95	1	EQ	57	1	17.5	12
4	88	T24	1	1	1	1	1	95	1	EQ	57	1	17.5	12
4	87	T12	1	2	2	2	1	80	0.656	EQ	48	1	26	31
4	87	T24	1	1	1	2	1	90	0.796	EQ	48	1	26	31
4	86	T12	2	1	1	2	1	90	0.727	EQ	20	1	25.8	13
4	86	T24	2	1	1	2	1	90	0.727	EQ	20	1	25.8	13
4	85	T12	1	2	3	3	3	14	-0.17	EQ	40	0	13.5	64
4	84	T12	2	1	1	2	2	50	0.656	EQ	70	1	23.5	26
4	84	T24	2	1	2	2	2	49	0.62	EQ	70	1	23.5	26
4	83	T12	1	1	3	2	1	58	0.433	EQ	42	1	40	90
4	83	T24	1	1	1	2	1	82	0.796	EQ	42	1	40	90
4	82	T12	1	1	1	2	1	80	0.796	EQ	50	1	12	23
4	82	T24	1	1	1	2	2	62	0.725	EQ	50	1	12	23
4	81	T12	1	1	1	1	2	48	0.848	EQ	35	1	24.5	9
4	81	T24	1	1	1	1	2	30	0.848	EQ	35	1	24.5	9
4	80	T12	2	3	1	1	2	40	0.296	EQ	37	1	85.5	116
4	80	T24	2	2	2	2	1	50	0.587	EQ	37	1	85.5	116
4	79	T12	2	2	2	3	1	58	0.055	EQ	58	1	70	98

4	78	T12	1	1	1	1	1	100	1	EQ	32	1	13.25	2
4	77	T12	1	1	1	2	1	50	0.796	EQ	47	1	9	9
4	76	T12	1	1	1	1	1	90	1	EQ	39	1	13.5	1
4	75	T12	1	1	1	1	1	90	1	EQ	28	1	15	10
4	74	T12	1	1	2	2	1	90	0.76	EQ	67	1	20.5	17
4	73	T12	1	1	1	1	1	100	1	EQ	22	1	11	12
4	72	T12	1	1	1	1	1	100	1	EQ	22	1	24	13
4	71	T12	1	1	2	1	2	#N/B	0.812	EQ	47	1	14.5	26
4	70	T12	2	2	2	3	2	25	-0.016	EQ	55	1	12	27
4	69	T12	2	2	3	2	2	40	0.189	EQ	63	1	35	29
4	68	T12	2	2	3	3	3	20	-0.239	EQ	37	1	21.5	23
4	67	T12	2	2	3	2	3	#N/B	0.024	EQ	85	1	1.5	15
4	66	T12	1	1	1	2	2	95	0.725	EQ	54	1	7.5	22
4	65	T12	2	2	3	2	2	50	0.189	EQ	70	0	0	17
4	64	T12	1	2	2	2	1	77	0.656	EQ	23	1	25	40
4	63	T12	1	1	1	2	2	50	0.725	EQ	61	0	15	21
4	62	T12	1	1	1	2	2	96	0.725	EQ	65	1	17	29
4	61	T12	2	2	2	3	2	20	-0.016	EQ	45	1	50.5	24
4	60	T12	1	1	2	2	2	85	0.689	EQ	18	1	13.5	24
4	59	T12	1	1	1	2	2	95	0.725	EQ	38	0	66	9
4	58	T12	2	2	2	2	1	51	0.587	EQ	68	1	28	24
4	57	T12	1	1	2	2	2	65	0.689	EQ	33	1	27.5	33
4	56	T12	1	1	1	2	2	96	0.725	EQ	22	1	27.5	20
4	55	T12	1	1	1	2	2	80	0.725	EQ	35	1	24.25	11
4	54	T12	1	1	1	1	1	100	1	EQ	42	1	18.6	13
4	54	T24	1	1	1	1	1	100	1	EQ	42	1	18.6	13
4	53	T12	1	1	1	1	1	90	1	EQ	44	0	14	11
4	53	T24	1	1	1	1	2	70	0.848	EQ	44	0	14	11
4	52	T12	1	1	3	2	3	29	0.197	EQ	38	1	42	71
4	52	T24	1	1	2	2	2	60	0.689	EQ	38	1	42	71
4	51	T12	1	1	2	2	2	68	0.689	EQ	19	1	8.5	15
4	51	T24	1	1	2	2	2	60	0.689	EQ	19	1	8.5	15
4	50	T12	1	1	1	1	2	85	0.848	EQ	18	1	14	11
4	50	T24	1	1	1	1	2	65	0.848	EQ	18	1	14	11
4	49	T12	1	1	1	2	2	61	0.725	EQ	61	1	30.5	11
4	49	T24	1	1	1	2	2	80	0.725	EQ	61	1	30.5	11
4	48	T12	1	1	2	2	1	97	0.76	EQ	55	1	10	13
4	48	T24	1	1	1	1	1	96	1	EQ	55	1	10	13
4	47	T12	1	1	1	2	1	92	0.796	EQ	43	1	39	32
4	47	T24	1	1	1	1	1	90	1	EQ	43	1	39	32
4	45	T12	2	2	2	2	2	10	0.516	EQ	62	0	6	19
4	44	T12	2	3	3	2	1	50	0.15	EQ	66	1	2.25	12
4	44	T24	1	3	3	2	1	40	0.219	EQ	66	1	2.25	12
4	43	T12	2	2	3	3	3	5	-0.239	EQ	54	1	62.5	51
4	43	T24	2	1	1	2	2	50	0.656	EQ	54	1	62.5	51
4	42	T12	1	2	2	2	2	70	0.585	EQ	22	1	2.5	27
4	41	T12	1	1	1	2	1	90	0.796	EQ	19	1	5.25	8
4	40	T12	1	1	3	2	2	60	0.362	EQ	46	1	13	24
4	40	T24	1	2	1	2	2	85	0.621	EQ	46	1	13	24
4	39	T12	2	2	1	3	3	50	-0.145	EQ	65	1	9.4	58
4	39	T24	2	2	3	2	2	40	0.189	EQ	65	1	9.4	58

4	38	T12	1	1	1	2	1	78	0.796	EQ	47	1	47.75	18
4	38	T24	1	1	1	2	1	80	0.796	EQ	47	1	47.75	18
4	37	T12	1	1	1	1	1	90	1	EQ	47	1	14	5
4	37	T24	1	1	1	1	1	90	1	EQ	47	1	14	5
4	36	T12	1	1	1	2	2	95	0.725	EQ	56	0	1.25	8
4	36	T24	1	1	1	1	1	85	1	EQ	56	0	1.25	8
4	35	T12	2	1	1	3	2	20	0.124	EQ	55	0	9.5	16
4	35	T24	2	1	1	2	1	60	0.727	EQ	55	0	9.5	16
4	34	T12	1	1	1	2	1	85	0.796	EQ	68	0	9	17
4	34	T24	1	1	1	1	1	#N/B	1	EQ	68	0	9	17
4	33	T12	1	1	1	2	1	95	0.796	EQ	78	1	9	11
4	32	T12	1	2	2	2	1	70	0.656	EQ	52	1	8	21
4	31	T12	1	1	1	2	2	60	0.725	EQ	57	0	23.5	24
4	31	T24	1	1	2	2	2	70	0.689	EQ	57	0	23.5	24
4	30	T12	1	1	1	2	1	61	0.796	EQ	32	1	54	47
4	30	T24	1	1	1	1	1	92	1	EQ	32	1	54	47
4	29	T12	1	1	1	2	2	70	0.725	EQ	44	0	45	72
4	29	T24	1	1	2	2	2	60	0.689	EQ	44	0	45	72
4	28	T12	2	1	1	2	2	70	0.656	EQ	52	0	12	5
4	28	T24	1	1	1	2	2	75	0.725	EQ	52	0	12	5
4	27	T12	1	1	1	1	1	85	1	EQ	67	1	2	7
4	27	T24	1	1	1	1	1	90	1	EQ	67	1	2	7
4	26	T12	1	1	1	2	1	80	0.796	EQ	23	1	56	62
4	25	T24	3	2	1	2	2	75	0.038	EQ	60	0	7	16
4	24	T12	3	3	3	2	2	16	-0.166	EQ	76	1	4	11
4	23	T12	2	2	2	2	1	70	0.587	EQ	49	1	33	10
4	23	T24	1	1	1	1	1	96	1	EQ	49	1	33	10
4	22	T12	2	3	3	3	3	#N/B	-0.349	EQ	68	0	26	28
4	21	T12	1	1	1	2	2	80	0.725	EQ	66	1	9.5	9
4	20	T12	2	1	1	2	1	80	0.727	EQ	88	1	8	12
4	19	T12	1	1	1	1	2	75	0.848	EQ	39	1	39.25	10
4	19	T24	1	1	1	2	2	65	0.725	EQ	39	1	39.25	10
4	18	T12	1	1	2	2	1	90	0.76	EQ	65	1	6.5	7
4	18	T24	2	1	1	1	1	75	0.85	EQ	65	1	6.5	7
4	17	T12	2	1	2	2	2	75	0.62	EQ	56	1	20	32
4	17	T24	2	1	2	3	2	50	0.088	EQ	56	1	20	32
4	16	T12	1	1	1	2	1	90	0.796	EQ	45	1	1.25	14
4	16	T24	2	1	1	2	1	85	0.727	EQ	45	1	1.25	14
4	15	T12	1	1	1	2	2	40	0.725	EQ	47	0	15	33
4	15	T24	1	1	1	1	1	98	1	EQ	47	0	15	33
4	14	T12	1	1	2	2	1	70	0.76	EQ	53	1	20	26
4	14	T24	1	1	1	1	1	75	1	EQ	53	1	20	26
4	13	T12	2	2	2	2	2	51	0.516	EQ	54	1	65	79
4	13	T24	1	2	1	1	2	35	0.744	EQ	54	1	65	79
4	12	T12	1	1	1	2	1	74	0.796	EQ	19	1	49	32
4	10	T12	2	1	2	2	1	40	0.691	EQ	66	0	13	1
4	10	T24	1	2	2	1	1	55	0.779	EQ	66	0	13	1
4	9	T12	1	1	1	1	3	100	0.414	EQ	18	0	13	43
4	8	T12	1	1	1	1	1	72	1	EQ	40	1	18.5	14
4	8	T24	1	1	1	2	1	70	0.796	EQ	40	1	18.5	14
4	7	T12	1	1	2	2	2	85	0.689	EQ	41	0	41	23

4	7	T24	1	1	1	2	2	60	0.725	EQ	41	0	41	23
4	6	T12	1	1	1	2	1	90	0.796	EQ	59	1	15.5	16
4	6	T24	1	1	1	2	1	85	0.796	EQ	59	1	15.5	16
4	5	T12	2	2	2	3	2	50	-0.016	EQ	69	1	27.35	42
4	4	T12	1	1	1	1	2	85	0.848	EQ	35	1	5	26
4	4	T24	1	1	1	1	2	80	0.848	EQ	35	1	5	26
4	3	T12	3	2	3	2	1	10	0.015	EQ	59	1	46	72
4	3	T24	3	2	3	2	1	#N/B	0.015	EQ	59	1	46	72
4	2	T24	1	2	2	2	2	90	0.585	EQ	36	0	51	94
4	1	T12	1	1	1	1	1	#N/B	1	EQ	50	0	11	22
4	1	T24	1	1	1	1	1	90	1	EQ	50	0	11	22
5	67	T0	2	2	3	2	1	40	0.26	EQ	35	1	26	45
5	67	T12	1	1	2	2	2	76	0.689	EQ	35	1	26	45
5	67	T24	1	1	1	2	1	80	0.796	EQ	35	1	26	45
5	67	T3	2	2	2	2	2	52	0.516	EQ	35	1	26	45
5	67	T6	1	1	1	2	2	44	0.725	EQ	35	1	26	45
5	66	T0	1	1	1	1	1	75	1	EQ	41.9	1	5.5	4
5	66	T12	1	1	1	1	1	92	1	EQ	41.9	1	5.5	4
5	66	T24	1	1	1	1	1	88	1	EQ	41.9	1	5.5	4
5	66	T3	1	1	1	1	1	86	1	EQ	41.9	1	5.5	4
5	66	T6	1	1	1	1	1	90	1	EQ	41.9	1	5.5	4
5	65	T0	2	1	3	3	2	45	0.03	EQ	54.7	1	3	8
5	65	T12	1	1	1	2	1	60	0.796	EQ	54.7	1	3	8
5	65	T24	2	1	1	2	1	60	0.727	EQ	54.7	1	3	8
5	65	T3	1	1	1	2	1	70	0.796	EQ	54.7	1	3	8
5	65	T6	1	1	1	2	1	65	0.796	EQ	54.7	1	3	8
5	64	T0	2	3	3	3	2	40	-0.184	EQ	44.5	1	68.5	144
5	64	T12	2	3	3	3	2	35	-0.184	EQ	44.5	1	68.5	144
5	64	T24	2	3	3	3	2	50	-0.184	EQ	44.5	1	68.5	144
5	63	T0	2	2	2	2	2	60	0.516	EQ	86	0	7	12
5	63	T18	2	1	1	2	1	60	0.727	EQ	86	0	7	12
5	63	T3	2	2	2	3	2	#N/B	-0.016	EQ	86	0	7	12
5	62	T0	3	3	3	2	1	25	-0.095	EQ	24	1	11	10
5	62	T24	1	1	1	1	1	65	1	EQ	24	1	11	10
5	62	T3	2	1	3	1	1	50	0.487	EQ	24	1	11	10
5	61	T0	2	1	1	2	2	66	0.656	EQ	19	1	38	20
5	61	T12	1	1	1	2	1	80	0.796	EQ	19	1	38	20
5	61	T18	1	1	1	1	1	89	1	EQ	19	1	38	20
5	61	T3	1	1	1	2	1	69	0.796	EQ	19	1	38	20
5	61	T6	1	1	1	2	1	79	0.796	EQ	19	1	38	20
5	60	T0	2	2	3	3	2	30	-0.074	EQ	42.5	0	12.5	23
5	60	T12	1	1	1	2	2	90	0.725	EQ	42.5	0	12.5	23
5	60	T24	1	1	1	1	1	95	1	EQ	42.5	0	12.5	23
5	60	T3	1	1	2	1	2	80	0.812	EQ	42.5	0	12.5	23
5	60	T6	1	1	1	1	1	90	1	EQ	42.5	0	12.5	23
5	59	T0	2	3	3	3	2	10	-0.184	EQ	30.4	1	30.5	13
5	59	T12	1	1	1	2	1	55	0.796	EQ	30.4	1	30.5	13
5	59	T24	1	1	1	2	1	70	0.796	EQ	30.4	1	30.5	13
5	59	T3	1	1	1	2	1	50	0.796	EQ	30.4	1	30.5	13
5	58	T0	3	2	3	3	2	40	-0.319	EQ	37.9	1	15.8	23
5	58	T12	2	1	2	2	1	75	0.691	EQ	37.9	1	15.8	23

5	58	T24	1	1	2	2	1	94	0.76	EQ	37.9	1	15.8	23
5	58	T3	2	1	2	2	1	80	0.691	EQ	37.9	1	15.8	23
5	58	T6	2	1	2	2	1	40	0.691	EQ	37.9	1	15.8	23
5	57	T0	2	3	3	2	3	20	-0.086	EQ	40	1	24	15
5	57	T12	1	1	2	2	2	#N/B	0.689	EQ	40	1	24	15
5	57	T24	1	1	1	2	1	91	0.796	EQ	40	1	24	15
5	57	T3	1	1	3	2	2	60	0.362	EQ	40	1	24	15
5	57	T6	1	1	2	2	2	#N/B	0.689	EQ	40	1	24	15
5	56	T24	1	1	1	2	2	95	0.725	EQ	29.4	1	46.5	25
5	55	T0	3	2	3	2	2	60	-0.056	EQ	39.1	1	12.8	18
5	55	T12	1	1	1	1	3	90	0.414	EQ	39.1	1	12.8	18
5	55	T24	1	1	1	1	2	85	0.848	EQ	39.1	1	12.8	18
5	55	T3	2	1	2	2	1	85	0.691	EQ	39.1	1	12.8	18
5	55	T36	1	1	1	1	1	90	1	EQ	39.1	1	12.8	18
5	55	T6	1	1	2	1	2	90	0.812	EQ	39.1	1	12.8	18
5	54	T0	1	1	3	2	2	70	0.362	EQ	47.8	1	7	16
5	54	T12	1	1	1	1	2	80	0.848	EQ	47.8	1	7	16
5	54	T24	1	1	1	1	1	90	1	EQ	47.8	1	7	16
5	54	T3	1	1	2	2	1	80	0.76	EQ	47.8	1	7	16
5	54	T6	1	1	1	1	1	90	1	EQ	47.8	1	7	16
5	53	T0	2	3	3	3	3	40	-0.349	EQ	46.1	1	47.8	17
5	53	T12	1	1	1	2	1	80	0.796	EQ	46.1	1	47.8	17
5	53	T24	1	1	1	2	1	80	0.796	EQ	46.1	1	47.8	17
5	53	T3	1	1	2	2	1	70	0.76	EQ	46.1	1	47.8	17
5	53	T36	1	1	1	2	1	80	0.796	EQ	46.1	1	47.8	17
5	53	T6	1	2	2	2	2	70	0.585	EQ	46.1	1	47.8	17
5	52	T0	1	2	1	2	1	65	0.692	EQ	19.6	1	20.5	18
5	52	T12	1	1	2	2	2	60	0.689	EQ	19.6	1	20.5	18
5	52	T36	1	1	2	2	1	85	0.76	EQ	19.6	1	20.5	18
5	51	T0	1	2	2	2	1	78	0.656	EQ	30.3	1	15	18
5	51	T36	1	1	1	2	1	90	0.796	EQ	30.3	1	15	18
5	50	T0	2	2	3	2	2	65	0.189	EQ	31.1	1	68.5	30
5	50	T12	2	2	2	2	1	80	0.587	EQ	31.1	1	68.5	30
5	50	T3	2	2	3	2	2	80	0.189	EQ	31.1	1	68.5	30
5	50	T36	2	1	1	2	1	90	0.727	EQ	31.1	1	68.5	30
5	50	T6	2	2	2	2	1	78	0.587	EQ	31.1	1	68.5	30
5	49	T0	2	2	3	2	1	50	0.26	EQ	31	1	33	18
5	49	T12	1	1	2	2	2	39	0.689	EQ	31	1	33	18
5	49	T24	2	1	2	2	1	90	0.691	EQ	31	1	33	18
5	49	T3	1	1	2	2	2	65	0.689	EQ	31	1	33	18
5	49	T36	2	1	3	3	3	36	-0.135	EQ	31	1	33	18
5	49	T6	1	1	2	2	2	60	0.689	EQ	31	1	33	18
5	48	T0	3	3	3	3	3	50	-0.594	EQ	76.4	0	18	34
5	48	T12	1	1	1	2	2	53	0.725	EQ	76.4	0	18	34
5	48	T24	1	1	1	2	1	70	0.796	EQ	76.4	0	18	34
5	48	T3	1	2	2	2	2	50	0.585	EQ	76.4	0	18	34
5	48	T36	1	1	1	1	1	85	1	EQ	76.4	0	18	34
5	48	T6	1	2	1	2	2	60	0.621	EQ	76.4	0	18	34
5	47	T0	2	2	3	2	3	30	0.024	EQ	30.9	1	34	34
5	47	T12	1	1	2	2	2	70	0.689	EQ	30.9	1	34	34
5	47	T24	1	1	2	2	2	70	0.689	EQ	30.9	1	34	34

5	47	T6	1	1	1	2	2	75	0.725	EQ	30.9	1	34	34
5	46	T0	1	2	3	3	2	30	-0.005	EQ	31.8	1	56.5	48
5	46	T12	1	1	1	2	1	65	0.796	EQ	31.8	1	56.5	48
5	46	T24	1	1	1	1	1	90	1	EQ	31.8	1	56.5	48
5	46	T3	1	2	3	3	2	30	-0.005	EQ	31.8	1	56.5	48
5	46	T48	1	1	1	1	1	82	1	EQ	31.8	1	56.5	48
5	46	T6	1	1	2	2	2	60	0.689	EQ	31.8	1	56.5	48
5	45	T0	3	3	3	2	1	50	-0.095	EQ	21.3	0	31.5	4
5	45	T12	1	1	1	1	1	95	1	EQ	21.3	0	31.5	4
5	45	T24	1	1	1	1	1	85	1	EQ	21.3	0	31.5	4
5	45	T3	1	1	1	1	1	85	1	EQ	21.3	0	31.5	4
5	45	T48	1	1	1	1	1	100	1	EQ	21.3	0	31.5	4
5	45	T6	1	1	1	2	1	80	0.796	EQ	21.3	0	31.5	4
5	44	T0	1	2	3	3	1	30	0.066	EQ	20	1	51.5	61
5	44	T12	1	1	1	1	3	72	0.414	EQ	20	1	51.5	61
5	44	T24	1	1	3	2	3	23	0.197	EQ	20	1	51.5	61
5	44	T3	1	2	3	3	3	30	-0.17	EQ	20	1	51.5	61
5	44	T48	1	1	1	2	2	77	0.725	EQ	20	1	51.5	61
5	44	T6	1	1	2	2	2	40	0.689	EQ	20	1	51.5	61
5	43	T0	2	2	3	2	2	50	0.189	EQ	51	0	31	20
5	43	T12	1	1	1	1	1	90	1	EQ	51	0	31	20
5	43	T24	1	1	1	2	2	90	0.725	EQ	51	0	31	20
5	43	T3	1	2	3	2	2	70	0.258	EQ	51	0	31	20
5	43	T36	1	1	1	2	1	90	0.796	EQ	51	0	31	20
5	43	T6	1	1	2	2	2	85	0.689	EQ	51	0	31	20
5	42	T0	1	2	3	2	1	42	0.329	EQ	55.1	1	61	37
5	42	T12	1	1	2	1	1	48	0.883	EQ	55.1	1	61	37
5	42	T24	1	1	2	2	1	80	0.76	EQ	55.1	1	61	37
5	42	T3	1	2	3	2	1	50	0.329	EQ	55.1	1	61	37
5	42	T36	1	1	1	2	1	90	0.796	EQ	55.1	1	61	37
5	42	T6	1	1	3	2	1	54	0.433	EQ	55.1	1	61	37
5	41	T0	2	3	2	2	2	35	0.137	EQ	75.8	1	7	11
5	41	T12	1	1	1	2	1	82	0.796	EQ	75.8	1	7	11
5	41	T24	1	1	1	1	1	80	1	EQ	75.8	1	7	11
5	41	T3	1	3	1	2	1	70	0.313	EQ	75.8	1	7	11
5	41	T36	1	1	1	1	1	80	1	EQ	75.8	1	7	11
5	41	T6	1	1	1	2	1	80	0.796	EQ	75.8	1	7	11
5	40	T0	2	3	3	2	1	40	0.15	EQ	24.4	1	15	11
5	40	T12	1	1	1	2	1	98	0.796	EQ	24.4	1	15	11
5	40	T24	1	1	2	2	1	80	0.76	EQ	24.4	1	15	11
5	40	T3	1	1	2	2	1	95	0.76	EQ	24.4	1	15	11
5	40	T36	1	1	1	2	1	95	0.796	EQ	24.4	1	15	11
5	40	T6	1	1	2	2	1	90	0.76	EQ	24.4	1	15	11
5	39	T0	2	2	3	2	3	60	0.024	EQ	60.5	0	17.5	25
5	39	T12	1	1	1	2	2	80	0.725	EQ	60.5	0	17.5	25
5	39	T24	1	1	1	2	2	80	0.725	EQ	60.5	0	17.5	25
5	39	T3	1	1	3	2	2	70	0.362	EQ	60.5	0	17.5	25
5	39	T48	1	1	1	2	2	80	0.725	EQ	60.5	0	17.5	25
5	39	T6	1	1	1	2	2	75	0.725	EQ	60.5	0	17.5	25
5	38	T0	3	3	3	2	1	#N/B	-0.095	EQ	39.3	0	79.5	230
5	38	T12	2	1	3	2	1	50	0.364	EQ	39.3	0	79.5	230

5	38	T3	3	3	3	2	1	#N/B	-0.095	EQ	39.3	0	79.5	230
5	38	T48	1	2	2	1	1	75	0.779	EQ	39.3	0	79.5	230
5	38	T6	2	3	3	3	2	35	-0.184	EQ	39.3	0	79.5	230
5	37	T0	2	2	1	2	1	30	0.623	EQ	78.6	1	16.5	10
5	37	T12	1	1	1	1	1	65	1	EQ	78.6	1	16.5	10
5	37	T24	1	1	1	2	1	80	0.796	EQ	78.6	1	16.5	10
5	37	T3	1	1	1	1	1	70	1	EQ	78.6	1	16.5	10
5	37	T6	1	1	1	1	1	90	1	EQ	78.6	1	16.5	10
5	37	T60	2	1	1	1	1	80	0.85	EQ	78.6	1	16.5	10
5	36	T0	2	2	3	2	1	65	0.26	EQ	33.4	1	5.5	7
5	36	T24	1	1	3	2	3	50	0.197	EQ	33.4	1	5.5	7
5	36	T3	1	1	3	2	2	50	0.362	EQ	33.4	1	5.5	7
5	36	T48	1	1	2	2	1	50	0.76	EQ	33.4	1	5.5	7
5	36	T6	1	1	3	2	3	30	0.197	EQ	33.4	1	5.5	7
5	35	T0	2	2	3	2	1	70	0.26	EQ	41.4	1	26	18
5	35	T48	1	1	1	1	1	100	1	EQ	41.4	1	26	18
5	34	T0	2	3	3	3	1	90	-0.113	EQ	39.6	1	16	11
5	34	T24	1	1	1	2	1	95	0.796	EQ	39.6	1	16	11
5	34	T3	1	1	3	2	2	80	0.362	EQ	39.6	1	16	11
5	34	T48	1	1	1	2	1	85	0.796	EQ	39.6	1	16	11
5	34	T6	1	1	2	2	2	80	0.689	EQ	39.6	1	16	11
5	33	T0	2	3	3	2	2	60	0.079	EQ	41.1	1	25	23
5	33	T12	1	2	1	2	1	70	0.692	EQ	41.1	1	25	23
5	33	T24	1	1	1	2	1	87	0.796	EQ	41.1	1	25	23
5	33	T3	1	2	2	2	1	70	0.656	EQ	41.1	1	25	23
5	33	T6	1	2	3	2	2	50	0.258	EQ	41.1	1	25	23
5	33	T60	1	2	1	2	1	85	0.692	EQ	41.1	1	25	23
5	32	T0	3	3	3	3	1	60	-0.358	EQ	42.7	1	34	10
5	32	T12	1	1	1	2	1	80	0.796	EQ	42.7	1	34	10
5	32	T24	2	1	2	2	1	85	0.691	EQ	42.7	1	34	10
5	32	T3	1	1	3	2	1	80	0.433	EQ	42.7	1	34	10
5	32	T6	1	1	2	2	1	95	0.76	EQ	42.7	1	34	10
5	32	T72	1	1	1	2	1	100	0.796	EQ	42.7	1	34	10
5	31	T0	2	3	3	2	2	50	0.079	EQ	46.3	0	39	26
5	31	T12	1	1	2	2	1	70	0.76	EQ	46.3	0	39	26
5	31	T24	1	1	2	2	2	65	0.689	EQ	46.3	0	39	26
5	31	T3	1	2	2	2	1	80	0.656	EQ	46.3	0	39	26
5	31	T6	1	1	2	2	1	85	0.76	EQ	46.3	0	39	26
5	31	T72	1	1	1	2	2	80	0.725	EQ	46.3	0	39	26
5	30	T0	3	3	3	3	1	40	-0.358	EQ	23.5	0	75	61
5	30	T12	2	1	2	2	2	50	0.62	EQ	23.5	0	75	61
5	30	T24	1	1	1	1	1	80	1	EQ	23.5	0	75	61
5	30	T3	1	1	2	2	2	31	0.689	EQ	23.5	0	75	61
5	30	T6	2	1	2	2	2	20	0.62	EQ	23.5	0	75	61
5	30	T72	1	1	1	2	1	75	0.796	EQ	23.5	0	75	61
5	29	T0	1	1	2	2	2	70	0.689	EQ	21.2	1	17	19
5	29	T12	1	1	3	2	3	60	0.197	EQ	21.2	1	17	19
5	29	T24	1	1	1	2	2	90	0.725	EQ	21.2	1	17	19
5	29	T3	1	1	2	2	2	70	0.689	EQ	21.2	1	17	19
5	29	T6	1	1	3	2	3	70	0.197	EQ	21.2	1	17	19
5	29	T72	1	1	1	1	1	88	1	EQ	21.2	1	17	19

5	28	T0	2	2	3	2	3	0	0.024	EQ	52	0	4	3
5	28	T12	2	1	2	3	3	30	-0.077	EQ	52	0	4	3
5	28	T24	2	1	1	2	3	20	0.222	EQ	52	0	4	3
5	28	T3	2	1	1	2	2	85	0.656	EQ	52	0	4	3
5	28	T6	2	1	2	2	2	50	0.62	EQ	52	0	4	3
5	28	T60	1	1	1	1	2	20	0.848	EQ	52	0	4	3
5	27	T0	2	3	3	2	1	85	0.15	EQ	73	1	7	6
5	27	T12	1	1	1	1	1	66	1	EQ	73	1	7	6
5	27	T24	1	1	1	1	1	61	1	EQ	73	1	7	6
5	27	T3	1	1	1	2	1	85	0.796	EQ	73	1	7	6
5	27	T6	1	1	1	1	1	80	1	EQ	73	1	7	6
5	27	T72	1	1	1	1	1	80	1	EQ	73	1	7	6
5	26	T0	1	1	2	1	2	30	0.812	EQ	45.3	1	16.3	6
5	26	T12	1	2	1	1	2	75	0.744	EQ	45.3	1	16.3	6
5	26	T3	1	1	1	1	2	50	0.848	EQ	45.3	1	16.3	6
5	26	T6	1	1	1	2	2	45	0.725	EQ	45.3	1	16.3	6
5	26	T72	1	1	1	1	2	60	0.848	EQ	45.3	1	16.3	6
5	25	T0	1	2	1	2	1	95	0.692	EQ	40.2	1	11.3	14
5	25	T12	1	2	1	3	2	78	0.089	EQ	40.2	1	11.3	14
5	25	T3	1	2	2	2	2	85	0.585	EQ	40.2	1	11.3	14
5	25	T6	1	1	1	3	2	65	0.193	EQ	40.2	1	11.3	14
5	25	T72	1	1	1	2	1	100	0.796	EQ	40.2	1	11.3	14
5	24	T0	1	1	3	2	3	45	0.197	EQ	49.9	1	24	27
5	24	T12	1	1	3	2	2	76	0.362	EQ	49.9	1	24	27
5	24	T24	1	2	3	2	2	55	0.258	EQ	49.9	1	24	27
5	24	T6	1	1	3	3	2	50	0.099	EQ	49.9	1	24	27
5	24	T72	1	1	2	2	2	75	0.689	EQ	49.9	1	24	27
5	23	T0	3	3	3	3	3	2	-0.594	EQ	57.7	0	38	64
5	23	T12	1	3	1	2	2	57	0.242	EQ	57.7	0	38	64
5	23	T24	1	1	1	2	2	45	0.725	EQ	57.7	0	38	64
5	23	T3	2	3	3	3	3	65	-0.349	EQ	57.7	0	38	64
5	23	T6	1	1	1	3	2	80	0.193	EQ	57.7	0	38	64
5	23	T72	1	1	1	2	2	63	0.725	EQ	57.7	0	38	64
5	22	T0	2	3	3	3	2	20	-0.184	EQ	67.3	0	14.3	59
5	22	T12	2	1	2	2	2	62	0.62	EQ	67.3	0	14.3	59
5	22	T24	1	1	1	2	2	50	0.725	EQ	67.3	0	14.3	59
5	22	T3	2	2	3	3	2	30	-0.074	EQ	67.3	0	14.3	59
5	22	T6	2	2	2	3	2	46	-0.016	EQ	67.3	0	14.3	59
5	22	T72	2	1	1	2	1	75	0.727	EQ	67.3	0	14.3	59
5	21	T0	2	2	3	3	3	48	-0.239	EQ	35.3	1	18.5	24
5	21	T12	1	1	2	1	2	47	0.812	EQ	35.3	1	18.5	24
5	21	T24	1	1	1	1	2	60	0.848	EQ	35.3	1	18.5	24
5	21	T3	1	1	1	1	2	69	0.848	EQ	35.3	1	18.5	24
5	21	T6	1	1	1	1	2	44	0.848	EQ	35.3	1	18.5	24
5	21	T72	1	1	1	1	1	70	1	EQ	35.3	1	18.5	24
5	20	T0	1	2	1	2	1	55	0.692	EQ	29.9	1	18	18
5	20	T12	1	1	1	2	2	80	0.725	EQ	29.9	1	18	18
5	20	T3	1	1	2	2	1	80	0.76	EQ	29.9	1	18	18
5	20	T6	1	1	1	2	2	75	0.725	EQ	29.9	1	18	18
5	20	T72	2	1	1	2	1	75	0.727	EQ	29.9	1	18	18
5	19	T0	2	2	3	2	2	78	0.189	EQ	19.3	1	5	8

5	19	T24	1	1	2	1	3	70	0.378	EQ	19.3	1	5	8
5	19	T3	1	1	2	2	2	60	0.689	EQ	19.3	1	5	8
5	19	T6	1	1	2	2	2	60	0.689	EQ	19.3	1	5	8
5	19	T84	1	1	1	2	2	80	0.725	EQ	19.3	1	5	8
5	18	T0	2	3	3	2	3	20	-0.086	EQ	46.3	1	43	24
5	18	T12	1	1	1	2	2	69	0.725	EQ	46.3	1	43	24
5	18	T3	1	1	1	2	1	81	0.796	EQ	46.3	1	43	24
5	18	T6	2	1	2	2	2	70	0.62	EQ	46.3	1	43	24
5	18	T84	1	1	1	1	1	70	1	EQ	46.3	1	43	24
5	17	T0	3	3	3	2	3	30	-0.331	EQ	39.5	1	80	58
5	17	T12	1	1	1	2	2	85	0.725	EQ	39.5	1	80	58
5	17	T3	1	2	2	2	2	55	0.585	EQ	39.5	1	80	58
5	17	T6	1	1	1	2	2	61	0.725	EQ	39.5	1	80	58
5	17	T84	1	1	1	2	1	85	0.796	EQ	39.5	1	80	58
5	16	T0	3	3	3	2	1	25	-0.095	EQ	50.1	1	48	30
5	16	T12	1	1	3	2	2	45	0.362	EQ	50.1	1	48	30
5	16	T24	1	3	2	2	2	40	0.206	EQ	50.1	1	48	30
5	16	T3	1	1	3	2	1	40	0.433	EQ	50.1	1	48	30
5	16	T6	2	1	1	2	2	43	0.656	EQ	50.1	1	48	30
5	16	T72	1	1	2	2	1	50	0.76	EQ	50.1	1	48	30
5	15	T0	3	3	3	2	2	35	-0.166	EQ	50.3	0	36.5	46
5	15	T12	1	1	1	2	2	60	0.725	EQ	50.3	0	36.5	46
5	15	T24	1	1	2	2	2	55	0.689	EQ	50.3	0	36.5	46
5	15	T3	3	2	3	2	2	45	-0.056	EQ	50.3	0	36.5	46
5	15	T6	2	2	3	3	3	17	-0.239	EQ	50.3	0	36.5	46
5	15	T72	1	1	2	2	2	70	0.689	EQ	50.3	0	36.5	46
5	14	T0	2	2	3	2	1	40	0.26	EQ	46.6	1	15	6
5	14	T12	1	1	1	1	1	90	1	EQ	46.6	1	15	6
5	14	T24	1	1	1	1	1	95	1	EQ	46.6	1	15	6
5	14	T3	1	1	3	2	1	70	0.433	EQ	46.6	1	15	6
5	14	T6	1	1	3	1	1	86	0.556	EQ	46.6	1	15	6
5	14	T84	1	1	1	1	1	95	1	EQ	46.6	1	15	6
5	13	T0	1	1	2	2	1	40	0.76	EQ	45.9	1	16	7
5	13	T12	2	1	3	3	2	10	0.03	EQ	45.9	1	16	7
5	13	T24	2	1	3	3	2	5	0.03	EQ	45.9	1	16	7
5	13	T3	1	1	3	1	3	20	0.32	EQ	45.9	1	16	7
5	13	T6	1	1	3	2	2	10	0.362	EQ	45.9	1	16	7
5	13	T72	2	1	3	3	2	35	0.03	EQ	45.9	1	16	7
5	12	T0	2	1	2	2	1	80	0.691	EQ	46.2	1	5.3	1
5	12	T12	1	1	1	1	1	100	1	EQ	46.2	1	5.3	1
5	12	T3	1	1	1	2	1	96	0.796	EQ	46.2	1	5.3	1
5	12	T6	1	1	1	1	1	100	1	EQ	46.2	1	5.3	1
5	12	T72	1	1	1	1	1	93	1	EQ	46.2	1	5.3	1
5	11	T0	1	1	2	2	1	75	0.76	EQ	27.8	1	7	3
5	11	T12	1	1	1	1	1	99	1	EQ	27.8	1	7	3
5	11	T24	1	1	1	1	1	90	1	EQ	27.8	1	7	3
5	11	T3	1	1	1	1	1	95	1	EQ	27.8	1	7	3
5	11	T6	1	1	1	1	1	90	1	EQ	27.8	1	7	3
5	11	T72	1	1	1	1	1	100	1	EQ	27.8	1	7	3
5	10	T0	1	1	3	2	1	80	0.433	EQ	47.5	1	7	10
5	10	T12	1	1	1	2	1	90	0.796	EQ	47.5	1	7	10

5	10	T24	1	1	1	2	2	95	0.725	EQ	47.5	1	7	10
5	10	T3	1	1	1	2	1	95	0.796	EQ	47.5	1	7	10
5	10	T6	1	1	1	2	1	95	0.796	EQ	47.5	1	7	10
5	10	T72	1	1	1	2	2	90	0.725	EQ	47.5	1	7	10
5	9	T0	2	2	3	2	1	37	0.26	EQ	40	1	4.5	10
5	9	T12	1	1	2	2	1	88	0.76	EQ	40	1	4.5	10
5	9	T24	1	1	1	2	1	95	0.796	EQ	40	1	4.5	10
5	9	T3	2	1	2	2	1	70	0.691	EQ	40	1	4.5	10
5	9	T6	2	1	2	2	1	75	0.691	EQ	40	1	4.5	10
5	9	T84	1	1	1	2	1	95	0.796	EQ	40	1	4.5	10
5	8	T0	1	1	1	1	1	90	1	EQ	44.8	1	14	17
5	8	T12	1	1	1	1	1	95	1	EQ	44.8	1	14	17
5	8	T24	1	1	1	1	1	99	1	EQ	44.8	1	14	17
5	8	T3	1	1	2	2	1	85	0.76	EQ	44.8	1	14	17
5	8	T6	1	1	1	1	1	90	1	EQ	44.8	1	14	17
5	8	T72	1	1	1	1	1	90	1	EQ	44.8	1	14	17
5	7	T0	3	2	1	2	2	70	0.038	EQ	41.5	1	1.5	3
5	7	T12	2	1	2	2	2	60	0.62	EQ	41.5	1	1.5	3
5	7	T24	1	1	1	1	1	85	1	EQ	41.5	1	1.5	3
5	7	T3	2	1	2	2	1	60	0.691	EQ	41.5	1	1.5	3
5	7	T6	2	1	2	2	2	80	0.62	EQ	41.5	1	1.5	3
5	7	T84	1	1	1	2	1	95	0.796	EQ	41.5	1	1.5	3
5	6	T0	3	2	3	3	2	#N/B	-0.319	EQ	43.3	1	51.5	63
5	6	T12	2	2	3	3	1	20	-0.003	EQ	43.3	1	51.5	63
5	6	T24	2	2	2	3	1	70	0.055	EQ	43.3	1	51.5	63
5	6	T3	2	2	3	3	1	35	-0.003	EQ	43.3	1	51.5	63
5	6	T6	2	2	3	3	1	40	-0.003	EQ	43.3	1	51.5	63
5	6	T84	2	1	2	2	1	97	0.691	EQ	43.3	1	51.5	63
5	5	T0	2	2	3	2	2	40	0.189	EQ	36.4	1	10.6	27
5	5	T12	1	1	1	1	1	95	1	EQ	36.4	1	10.6	27
5	5	T24	1	1	1	1	1	60	1	EQ	36.4	1	10.6	27
5	5	T3	1	1	2	2	2	70	0.689	EQ	36.4	1	10.6	27
5	5	T6	1	1	2	2	2	75	0.689	EQ	36.4	1	10.6	27
5	5	T84	1	1	1	2	3	50	0.291	EQ	36.4	1	10.6	27
5	4	T0	3	3	3	2	2	70	-0.166	EQ	49.4	0	12	27
5	4	T12	1	1	1	1	2	80	0.848	EQ	49.4	0	12	27
5	4	T24	1	1	2	1	3	60	0.378	EQ	49.4	0	12	27
5	4	T3	1	2	2	2	3	65	0.151	EQ	49.4	0	12	27
5	4	T6	1	1	2	2	3	60	0.255	EQ	49.4	0	12	27
5	4	T84	1	1	2	1	2	80	0.812	EQ	49.4	0	12	27
5	3	T0	1	1	1	1	1	95	1	EQ	56.9	1	4.8	6
5	3	T12	1	1	1	1	1	95	1	EQ	56.9	1	4.8	6
5	3	T24	1	1	1	1	1	95	1	EQ	56.9	1	4.8	6
5	3	T3	1	1	1	1	1	95	1	EQ	56.9	1	4.8	6
5	3	T6	1	1	1	2	1	90	0.796	EQ	56.9	1	4.8	6
5	3	T84	1	1	1	1	1	90	1	EQ	56.9	1	4.8	6
5	2	T0	2	3	3	3	3	8	-0.349	EQ	37.3	0	16.5	27
5	2	T12	1	1	1	2	2	70	0.725	EQ	37.3	0	16.5	27
5	2	T24	1	1	1	2	2	60	0.725	EQ	37.3	0	16.5	27
5	2	T3	1	1	2	2	2	39	0.689	EQ	37.3	0	16.5	27
5	2	T6	1	1	2	3	2	41	0.157	EQ	37.3	0	16.5	27

5	2	T84	1	1	1	2	1	90	0.796	EQ	37.3	0	16.5	27
5	1	T0	2	2	3	2	2	90	0.189	EQ	52.2	1	36	31
5	1	T12	1	1	1	1	1	100	1	EQ	52.2	1	36	31
5	1	T24	1	1	1	1	1	#N/B	1	EQ	52.2	1	36	31
5	1	T3	1	1	3	2	1	95	0.433	EQ	52.2	1	36	31
5	1	T6	1	1	1	2	1	100	0.796	EQ	52.2	1	36	31
5	1	T84	1	1	1	1	1	95	1	EQ	52.2	1	36	31
6	4038	T12	2	1	1	2	3	#N/B	0.222	SF	38.9	0	10	23
6	4038	T24	1	1	1	2	1	#N/B	0.796	SF	38.9	0	10	23
6	4037	T12	1	1	1	2	2	#N/B	0.725	SF	47.8	1	6	13
6	4037	T24	1	1	1	2	3	#N/B	0.291	SF	47.8	1	6	13
6	4037	T6	2	1	1	2	2	#N/B	0.656	SF	47.8	1	6	13
6	4035	T6	1	1	1	2	2	#N/B	0.725	SF	32.9	0	5	9
6	4034	T6	1	1	2	2	1	#N/B	0.76	SF	46.5	1	8	11
6	4033	T12	1	1	1	2	3	#N/B	0.291	SF	56.4	1	15	13
6	4033	T24	1	1	2	2	2	#N/B	0.689	SF	56.4	1	15	13
6	4033	T6	2	1	1	2	2	#N/B	0.656	SF	56.4	1	15	13
6	4031	T24	1	1	1	2	1	#N/B	0.796	SF	43.9	1	12	11
6	4031	T36	1	1	1	1	1	#N/B	1	SF	43.9	1	12	11
6	4031	T6	1	1	1	2	2	#N/B	0.725	SF	43.9	1	12	11
6	4030	T24	1	1	1	2	1	#N/B	0.796	SF	44.5	1	10	10
6	4030	T36	1	1	1	1	1	#N/B	1	SF	44.5	1	10	10
6	4030	T6	2	1	1	1	2	#N/B	0.779	SF	44.5	1	10	10
6	4029	T24	1	1	1	2	2	#N/B	0.725	SF	46.1	1	16	13
6	4029	T36	1	1	1	2	1	#N/B	0.796	SF	46.1	1	16	13
6	4029	T6	1	1	1	2	2	#N/B	0.725	SF	46.1	1	16	13
6	4024	T6	1	1	1	2	2	#N/B	0.725	SF	44.7	1	31	26
6	4021	T12	1	1	1	1	1	#N/B	1	SF	57.1	1	10	9
6	4021	T24	1	1	2	2	1	#N/B	0.76	SF	57.1	1	10	9
6	4021	T36	2	1	2	2	1	#N/B	0.691	SF	57.1	1	10	9
6	4021	T6	1	1	1	2	1	#N/B	0.796	SF	57.1	1	10	9
6	4019	T6	2	3	1	2	3	#N/B	0.008	SF	45.6	0	15	33
6	4017	T36	1	1	1	2	2	#N/B	0.725	SF	31.4	1	8	10
6	4017	T6	1	1	1	2	2	#N/B	0.725	SF	31.4	1	8	10
6	4016	T12	2	1	1	2	2	#N/B	0.656	SF	30.8	0	6	13
6	4016	T36	1	1	1	2	2	#N/B	0.725	SF	30.8	0	6	13
6	4016	T6	2	1	1	2	2	#N/B	0.656	SF	30.8	0	6	13
6	4014	T12	1	1	1	1	1	#N/B	1	SF	43	1	3	9
6	4014	T6	1	1	1	2	2	#N/B	0.725	SF	43	1	3	9
6	4010	T6	1	1	2	1	2	#N/B	0.812	SF	29.3	1	60	35
6	4009	T12	1	1	2	2	2	#N/B	0.689	SF	45.6	1	6	8
6	4007	T24	1	1	1	2	1	#N/B	0.796	SF	32.4	1	12	6
6	4007	T6	1	1	1	2	1	#N/B	0.796	SF	32.4	1	12	6
6	4006	T12	1	1	1	2	2	#N/B	0.725	SF	59.7	1	2	8
6	4006	T24	1	1	1	2	1	#N/B	0.796	SF	59.7	1	2	8
6	4006	T36	1	1	1	1	1	#N/B	1	SF	59.7	1	2	8
6	4006	T6	1	1	1	2	1	#N/B	0.796	SF	59.7	1	2	8
6	4005	T12	2	1	1	1	2	#N/B	0.779	SF	42.7	0	12	9
6	4005	T24	1	1	1	2	2	#N/B	0.725	SF	42.7	0	12	9
6	4005	T6	1	1	1	2	1	#N/B	0.796	SF	42.7	0	12	9
6	4003	T12	1	1	1	1	2	#N/B	0.848	SF	35.7	1	19	13

6	4003	T36	1	1	1	1	1	#N/B	1	SF	35.7	1	19	13
6	4003	T6	1	1	2	1	2	#N/B	0.812	SF	35.7	1	19	13
6	4002	T12	1	1	1	2	1	#N/B	0.796	SF	37.7	0	10	14
6	4002	T24	2	1	1	2	3	#N/B	0.222	SF	37.7	0	10	14
6	4002	T36	1	1	1	2	1	#N/B	0.796	SF	37.7	0	10	14
6	4002	T6	1	1	1	2	1	#N/B	0.796	SF	37.7	0	10	14
6	4001	T24	1	1	1	2	1	#N/B	0.796	SF	21.4	0	15	12
6	3083	T12	2	1	1	2	1	#N/B	0.727	SF	42.4	1	15	23
6	3081	T12	1	1	1	2	2	#N/B	0.725	SF	32.6	1	11	26
6	3081	T24	1	1	1	2	1	#N/B	0.796	SF	32.6	1	11	26
6	3081	T36	1	1	1	1	1	#N/B	1	SF	32.6	1	11	26
6	3079	T12	1	1	1	2	2	#N/B	0.725	SF	44.9	0	21	26
6	3079	T24	2	1	1	2	1	#N/B	0.727	SF	44.9	0	21	26
6	3079	T36	1	1	1	1	1	#N/B	1	SF	44.9	0	21	26
6	3078	T12	1	1	1	2	1	#N/B	0.796	SF	26.9	0	12	31
6	3078	T6	1	1	1	2	3	#N/B	0.291	SF	26.9	0	12	31
6	3076	T12	1	1	1	2	2	#N/B	0.725	SF	25.5	0	25	19
6	3076	T6	1	1	1	2	1	#N/B	0.796	SF	25.5	0	25	19
6	3075	T12	1	1	1	2	2	#N/B	0.725	SF	58.1	1	2	8
6	3075	T24	1	1	1	1	2	#N/B	0.848	SF	58.1	1	2	8
6	3075	T36	1	1	1	1	2	#N/B	0.848	SF	58.1	1	2	8
6	3075	T6	2	1	2	2	2	#N/B	0.62	SF	58.1	1	2	8
6	3072	T12	1	1	2	2	2	#N/B	0.689	SF	60	1	4	17
6	3072	T24	1	1	2	2	2	#N/B	0.689	SF	60	1	4	17
6	3072	T36	1	1	2	2	2	#N/B	0.689	SF	60	1	4	17
6	3072	T6	1	1	2	2	3	#N/B	0.255	SF	60	1	4	17
6	3070	T24	1	1	1	1	3	#N/B	0.414	SF	19.3	1	13	16
6	3069	T6	1	1	1	2	1	#N/B	0.796	SF	29.2	1	21	14
6	3067	T24	2	1	1	2	1	#N/B	0.727	SF	34.2	1	7	28
6	3067	T36	1	1	1	2	1	#N/B	0.796	SF	34.2	1	7	28
6	3067	T6	1	1	1	2	2	#N/B	0.725	SF	34.2	1	7	28
6	3066	T6	1	1	1	2	2	#N/B	0.725	SF	54.4	1	9	13
6	3065	T24	1	1	1	2	1	#N/B	0.796	SF	53.6	1	24	22
6	3065	T6	1	1	1	2	2	#N/B	0.725	SF	53.6	1	24	22
6	3060	T24	1	1	1	3	1	#N/B	0.264	SF	48.2	1	8	18
6	3060	T36	1	1	1	2	1	#N/B	0.796	SF	48.2	1	8	18
6	3060	T6	1	1	1	1	3	#N/B	0.414	SF	48.2	1	8	18
6	3059	T24	1	2	1	2	2	#N/B	0.621	SF	34.7	1	20	32
6	3059	T6	1	1	2	2	3	#N/B	0.255	SF	34.7	1	20	32
6	3058	T6	1	1	1	1	2	#N/B	0.848	SF	21	0	5	24
6	3056	T24	1	1	1	2	2	#N/B	0.725	SF	41.2	1	20	25
6	3056	T36	1	1	1	2	1	#N/B	0.796	SF	41.2	1	20	25
6	3056	T6	1	1	1	1	2	#N/B	0.848	SF	41.2	1	20	25
6	3052	T12	1	1	1	2	2	#N/B	0.725	SF	40.1	1	41	47
6	3052	T24	1	1	1	2	1	#N/B	0.796	SF	40.1	1	41	47
6	3052	T36	1	1	1	1	1	#N/B	1	SF	40.1	1	41	47
6	3052	T6	1	1	1	2	3	#N/B	0.291	SF	40.1	1	41	47
6	3047	T12	1	1	1	2	3	#N/B	0.291	SF	21.6	1	8	10
6	3047	T24	1	1	1	2	1	#N/B	0.796	SF	21.6	1	8	10
6	3047	T36	1	1	1	1	3	#N/B	0.414	SF	21.6	1	8	10
6	3047	T6	1	1	1	2	2	#N/B	0.725	SF	21.6	1	8	10

6	3046	T12	2	1	1	2	1	#N/B	0.727	SF	30	1	2	9
6	3045	T12	1	1	1	2	2	#N/B	0.725	SF	39.8	1	4	15
6	3045	T24	1	1	1	2	2	#N/B	0.725	SF	39.8	1	4	15
6	3045	T36	1	1	1	1	1	#N/B	1	SF	39.8	1	4	15
6	3045	T6	2	1	1	1	2	#N/B	0.779	SF	39.8	1	4	15
6	3043	T12	1	1	1	2	2	#N/B	0.725	SF	44.5	1	10	8
6	3043	T24	1	1	1	1	1	#N/B	1	SF	44.5	1	10	8
6	3043	T36	1	1	1	1	1	#N/B	1	SF	44.5	1	10	8
6	3043	T6	2	1	1	2	3	#N/B	0.222	SF	44.5	1	10	8
6	3041	T12	1	1	1	2	2	#N/B	0.725	SF	39.2	1	5	12
6	3041	T24	1	1	1	2	1	#N/B	0.796	SF	39.2	1	5	12
6	3041	T6	1	1	2	2	2	#N/B	0.689	SF	39.2	1	5	12
6	3039	T6	1	1	1	2	3	#N/B	0.291	SF	35	1	18	18
6	3037	T12	2	1	1	2	2	#N/B	0.656	SF	55.2	1	7	13
6	3037	T36	1	1	1	1	1	#N/B	1	SF	55.2	1	7	13
6	3037	T6	1	1	1	2	1	#N/B	0.796	SF	55.2	1	7	13
6	3036	T6	1	1	1	2	1	#N/B	0.796	SF	19.7	1	61	80
6	3034	T12	1	1	1	2	1	#N/B	0.796	SF	43	0	14	18
6	3032	T12	1	1	1	2	2	#N/B	0.725	SF	19.3	0	6	95
6	3032	T24	1	1	1	2	1	#N/B	0.796	SF	19.3	0	6	95
6	3032	T36	2	1	1	2	2	#N/B	0.656	SF	19.3	0	6	95
6	3032	T6	1	1	2	2	2	#N/B	0.689	SF	19.3	0	6	95
6	3031	T36	1	1	1	2	1	#N/B	0.796	SF	27.6	1	22	36
6	3031	T6	1	1	1	1	1	#N/B	1	SF	27.6	1	22	36
6	3030	T12	1	1	1	2	3	#N/B	0.291	SF	36.9	1	13	17
6	3030	T24	1	1	1	1	3	#N/B	0.414	SF	36.9	1	13	17
6	3030	T36	1	1	1	1	1	#N/B	1	SF	36.9	1	13	17
6	3030	T6	1	1	1	2	2	#N/B	0.725	SF	36.9	1	13	17
6	3029	T12	1	1	1	2	2	#N/B	0.725	SF	31.8	1	14	12
6	3029	T24	1	1	1	2	2	#N/B	0.725	SF	31.8	1	14	12
6	3029	T36	1	1	1	2	1	#N/B	0.796	SF	31.8	1	14	12
6	3029	T6	1	1	1	2	1	#N/B	0.796	SF	31.8	1	14	12
6	3027	T12	3	3	3	1	3	#N/B	-0.208	SF	26.6	1	31	44
6	3027	T24	1	3	1	1	1	#N/B	0.436	SF	26.6	1	31	44
6	3027	T6	1	3	3	2	2	#N/B	0.148	SF	26.6	1	31	44
6	3026	T12	2	1	2	2	1	#N/B	0.691	SF	38.5	1	62	97
6	3026	T6	2	1	1	2	2	#N/B	0.656	SF	38.5	1	62	97
6	3025	T6	1	1	1	2	2	#N/B	0.725	SF	38.9	1	3	15
6	3023	T12	1	1	1	2	3	#N/B	0.291	SF	19.5	0	22	33
6	3023	T6	1	1	1	1	2	#N/B	0.848	SF	19.5	0	22	33
6	3022	T12	1	1	1	2	2	#N/B	0.725	SF	53.5	1	18	28
6	3022	T36	2	1	1	2	1	#N/B	0.727	SF	53.5	1	18	28
6	3022	T6	1	1	1	2	2	#N/B	0.725	SF	53.5	1	18	28
6	3020	T12	2	1	1	1	1	#N/B	0.85	SF	45.9	1	31	48
6	3020	T36	2	1	1	2	1	#N/B	0.727	SF	45.9	1	31	48
6	3020	T6	1	1	1	2	2	#N/B	0.725	SF	45.9	1	31	48
6	3017	T12	1	1	1	2	1	#N/B	0.796	SF	24.9	1	21	33
6	3014	T12	2	1	1	2	2	#N/B	0.656	SF	36.7	1	35	31
6	3014	T36	1	1	1	2	2	#N/B	0.725	SF	36.7	1	35	31
6	3014	T6	1	1	1	2	1	#N/B	0.796	SF	36.7	1	35	31
6	3010	T6	1	1	2	2	1	#N/B	0.76	SF	24.4	1	22	55

6	3008	T36	1	1	2	2	2	#N/B	0.689	SF	42	0	10	14
6	2102	T12	2	1	1	2	2	#N/B	0.656	SF	45.4	1	5	13
6	2100	T24	1	1	1	2	1	#N/B	0.796	SF	31.9	1	2	10
6	2100	T36	1	1	1	1	1	#N/B	1	SF	31.9	1	2	10
6	2099	T24	1	1	1	2	2	#N/B	0.725	SF	33.3	1	10	24
6	2097	T12	1	1	2	2	2	#N/B	0.689	SF	40.1	1	13	17
6	2096	T12	1	1	1	2	1	#N/B	0.796	SF	36.3	1	3	17
6	2096	T24	2	1	1	2	1	#N/B	0.727	SF	36.3	1	3	17
6	2096	T36	1	1	1	1	1	#N/B	1	SF	36.3	1	3	17
6	2095	T24	1	3	3	1	1	#N/B	0.342	SF	39.1	1	18	33
6	2092	T12	2	1	1	2	2	#N/B	0.656	SF	34.4	1	9	18
6	2092	T36	1	1	1	2	1	#N/B	0.796	SF	34.4	1	9	18
6	2092	T6	2	1	3	2	2	#N/B	0.293	SF	34.4	1	9	18
6	2091	T12	1	1	2	2	2	#N/B	0.689	SF	54.9	1	5	31
6	2091	T24	1	1	1	2	2	#N/B	0.725	SF	54.9	1	5	31
6	2091	T36	1	1	1	1	1	#N/B	1	SF	54.9	1	5	31
6	2091	T6	1	1	1	2	2	#N/B	0.725	SF	54.9	1	5	31
6	2090	T6	1	1	1	2	1	#N/B	0.796	SF	41.7	1	4	26
6	2089	T6	1	1	2	2	2	#N/B	0.689	SF	36.1	1	15	23
6	2088	T12	1	1	1	1	2	#N/B	0.848	SF	36.4	0	8	28
6	2088	T36	1	1	2	2	2	#N/B	0.689	SF	36.4	0	8	28
6	2088	T6	1	1	1	3	1	#N/B	0.264	SF	36.4	0	8	28
6	2087	T12	1	1	1	1	1	#N/B	1	SF	37.1	0	3	20
6	2087	T24	1	1	1	2	1	#N/B	0.796	SF	37.1	0	3	20
6	2087	T6	1	1	1	2	1	#N/B	0.796	SF	37.1	0	3	20
6	2084	T24	1	1	1	2	1	#N/B	0.796	SF	39.6	1	5	30
6	2083	T12	1	1	1	2	2	#N/B	0.725	SF	37.5	0	18	16
6	2083	T24	1	1	1	2	2	#N/B	0.725	SF	37.5	0	18	16
6	2083	T36	1	1	1	1	1	#N/B	1	SF	37.5	0	18	16
6	2083	T6	1	1	1	2	2	#N/B	0.725	SF	37.5	0	18	16
6	2079	T6	1	2	1	2	2	#N/B	0.621	SF	42.1	1	8	28
6	2078	T6	1	1	1	2	1	#N/B	0.796	SF	33	1	13	28
6	2074	T24	1	1	1	2	2	#N/B	0.725	SF	38	0	5	20
6	2074	T6	1	1	1	2	2	#N/B	0.725	SF	38	0	5	20
6	2073	T24	1	1	1	1	2	#N/B	0.848	SF	47.5	1	19	20
6	2073	T36	1	1	1	1	2	#N/B	0.848	SF	47.5	1	19	20
6	2073	T6	1	1	1	2	2	#N/B	0.725	SF	47.5	1	19	20
6	2070	T24	2	1	1	2	2	#N/B	0.656	SF	38.6	1	8	28
6	2070	T6	1	1	1	1	1	#N/B	1	SF	38.6	1	8	28
6	2066	T6	1	1	1	2	2	#N/B	0.725	SF	53.2	1	12	16
6	2065	T24	1	1	1	2	2	#N/B	0.725	SF	47.4	1	9	14
6	2065	T6	2	1	1	2	2	#N/B	0.656	SF	47.4	1	9	14
6	2059	T12	1	1	1	2	2	#N/B	0.725	SF	27.3	1	6	24
6	2059	T6	1	1	1	1	1	#N/B	1	SF	27.3	1	6	24
6	2058	T24	1	1	1	1	2	#N/B	0.848	SF	64.7	1	11	140
6	2058	T6	1	1	1	2	1	#N/B	0.796	SF	64.7	1	11	140
6	2053	T12	1	1	1	2	2	#N/B	0.725	SF	39.1	0	18	21
6	2053	T6	1	1	1	2	1	#N/B	0.796	SF	39.1	0	18	21
6	2050	T12	1	1	2	2	2	#N/B	0.689	SF	59.6	1	6	36
6	2050	T24	1	1	1	2	2	#N/B	0.725	SF	59.6	1	6	36
6	2050	T6	1	1	1	2	2	#N/B	0.725	SF	59.6	1	6	36

6	2047	T12	1	1	1	2	1	#N/B	0.796	SF	36.7	0	5	18
6	2047	T24	1	1	1	2	1	#N/B	0.796	SF	36.7	0	5	18
6	2047	T36	1	1	1	1	2	#N/B	0.848	SF	36.7	0	5	18
6	2047	T6	1	1	2	2	3	#N/B	0.255	SF	36.7	0	5	18
6	2046	T12	1	1	1	2	1	#N/B	0.796	SF	34.6	1	2	17
6	2046	T24	1	1	1	2	2	#N/B	0.725	SF	34.6	1	2	17
6	2046	T6	2	1	1	2	2	#N/B	0.656	SF	34.6	1	2	17
6	2044	T12	2	1	2	2	1	#N/B	0.691	SF	61.3	1	37	74
6	2044	T24	1	1	2	2	2	#N/B	0.689	SF	61.3	1	37	74
6	2044	T36	2	1	2	2	2	#N/B	0.62	SF	61.3	1	37	74
6	2044	T6	2	1	1	2	1	#N/B	0.727	SF	61.3	1	37	74
6	2043	T36	1	1	1	2	2	#N/B	0.725	SF	38.6	1	5	40
6	2042	T6	1	1	1	2	2	#N/B	0.725	SF	36.6	1	2	18
6	2041	T12	1	1	1	2	2	#N/B	0.725	SF	35.5	0	2	26
6	2041	T24	1	1	2	2	1	#N/B	0.76	SF	35.5	0	2	26
6	2041	T36	1	1	1	2	1	#N/B	0.796	SF	35.5	0	2	26
6	2041	T6	1	1	1	3	1	#N/B	0.264	SF	35.5	0	2	26
6	2038	T12	1	1	1	2	2	#N/B	0.725	SF	41.3	0	16	42
6	2038	T24	2	1	1	2	2	#N/B	0.656	SF	41.3	0	16	42
6	2038	T6	1	1	1	2	2	#N/B	0.725	SF	41.3	0	16	42
6	2035	T12	1	1	1	2	1	#N/B	0.796	SF	18.5	1	25	113
6	2035	T24	2	1	1	2	2	#N/B	0.656	SF	18.5	1	25	113
6	2035	T36	1	1	1	1	1	#N/B	1	SF	18.5	1	25	113
6	2032	T24	2	2	3	2	1	#N/B	0.26	SF	52.2	1	10	51
6	2032	T6	3	3	3	1	3	#N/B	-0.208	SF	52.2	1	10	51
6	2030	T6	2	1	1	1	2	#N/B	0.779	SF	24.9	0	6	14
6	2022	T24	1	1	1	2	2	#N/B	0.725	SF	46.8	1	8	44
6	2022	T36	1	1	1	2	2	#N/B	0.725	SF	46.8	1	8	44
6	2022	T6	1	1	1	2	2	#N/B	0.725	SF	46.8	1	8	44
6	2020	T12	1	1	2	1	2	#N/B	0.812	SF	50.3	0	42	246
6	2020	T24	1	1	1	2	1	#N/B	0.796	SF	50.3	0	42	246
6	2020	T36	2	1	2	2	2	#N/B	0.62	SF	50.3	0	42	246
6	2020	T6	1	1	1	1	1	#N/B	1	SF	50.3	0	42	246
6	2017	T12	1	1	1	2	1	#N/B	0.796	SF	43.6	1	17	47
6	2017	T24	1	1	1	2	2	#N/B	0.725	SF	43.6	1	17	47
6	2017	T36	1	1	1	1	2	#N/B	0.848	SF	43.6	1	17	47
6	2017	T6	2	1	1	2	1	#N/B	0.727	SF	43.6	1	17	47
6	2016	T12	1	1	1	2	1	#N/B	0.796	SF	28.4	1	5	16
6	2016	T36	1	1	1	1	2	#N/B	0.848	SF	28.4	1	5	16
6	2003	T6	1	1	1	1	2	#N/B	0.848	SF	38	1	2	22
6	2001	T6	1	1	1	1	2	#N/B	0.848	SF	64	1	2	12
6	1157	T12	1	1	1	2	2	#N/B	0.725	SF	42.8	1	5	6
6	1155	T12	1	1	1	2	3	#N/B	0.291	SF	62.5	0	6	7
6	1154	T24	1	1	1	2	2	#N/B	0.725	SF	29.7	1	2	12
6	1154	T36	1	1	1	1	1	#N/B	1	SF	29.7	1	5	30
6	1152	T6	1	1	1	1	2	#N/B	0.848	SF	21.2	0	2	6
6	1151	T12	2	1	2	3	3	#N/B	-0.077	SF	47.8	1	30	47
6	1151	T24	1	1	1	2	1	#N/B	0.796	SF	47.8	1	30	47
6	1151	T6	2	1	1	2	1	#N/B	0.727	SF	47.8	1	30	47
6	1143	T6	1	1	2	1	1	#N/B	0.883	SF	28.7	1	2	7
6	1139	T24	1	1	1	2	1	#N/B	0.796	SF	51	1	18	10

6	1139	T36	1	1	1	1	1	#N/B	1	SF	51	1	18	10
6	1138	T24	1	1	2	2	2	#N/B	0.689	SF	19	1	18	10
6	1138	T6	1	1	1	2	2	#N/B	0.725	SF	19	1	18	10
6	1136	T24	1	1	2	1	3	#N/B	0.378	SF	51.8	1	5	15
6	1135	T24	1	1	1	2	2	#N/B	0.725	SF	53.2	0	7	6
6	1135	T36	1	1	1	1	1	#N/B	1	SF	53.2	0	7	6
6	1135	T6	1	1	2	2	2	#N/B	0.689	SF	53.2	0	7	6
6	1133	T24	1	1	1	1	1	#N/B	1	SF	53.3	0	4	18
6	1133	T36	1	1	1	1	2	#N/B	0.848	SF	53.3	0	4	18
6	1133	T6	1	2	1	2	2	#N/B	0.621	SF	53.3	0	4	18
6	1129	T24	1	1	1	2	1	#N/B	0.796	SF	35.8	1	3	17
6	1129	T36	1	1	1	2	1	#N/B	0.796	SF	35.8	1	3	17
6	1129	T6	1	1	1	2	2	#N/B	0.725	SF	35.8	1	3	17
6	1127	T24	1	1	1	2	3	#N/B	0.291	SF	47.5	1	4	15
6	1127	T6	1	1	1	2	2	#N/B	0.725	SF	47.5	1	4	15
6	1126	T24	1	1	2	1	1	#N/B	0.883	SF	60.9	1	2	12
6	1126	T6	2	1	1	2	2	#N/B	0.656	SF	60.9	1	2	12
6	1123	T24	1	1	1	2	1	#N/B	0.796	SF	30.3	0	6	8
6	1123	T6	1	1	1	1	2	#N/B	0.848	SF	30.3	0	6	8
6	1114	T12	1	1	2	2	1	#N/B	0.76	SF	59.5	0	9	25
6	1114	T24	2	1	1	2	1	#N/B	0.727	SF	59.5	0	9	25
6	1114	T36	1	1	1	1	1	#N/B	1	SF	59.5	0	9	25
6	1114	T6	2	1	1	2	3	#N/B	0.222	SF	59.5	0	9	25
6	1107	T36	1	1	1	2	2	#N/B	0.725	SF	45.7	1	2	13
6	1101	T12	1	1	1	2	2	#N/B	0.725	SF	55.1	1	2	29
6	1101	T6	1	1	1	2	1	#N/B	0.796	SF	55.1	1	2	29
6	1100	T12	1	1	2	2	1	#N/B	0.76	SF	38	1	1	7
6	1100	T24	1	1	1	2	2	#N/B	0.725	SF	38	1	4	7
6	1100	T36	1	1	1	1	2	#N/B	0.848	SF	38	1	4	7
6	1100	T6	1	1	2	2	1	#N/B	0.76	SF	38	1	6	7
6	1095	T12	2	1	2	2	2	#N/B	0.62	SF	41.5	1	37	17
6	1095	T6	1	1	2	1	1	#N/B	0.883	SF	41.5	1	12	31
6	1092	T12	1	2	1	2	2	#N/B	0.621	SF	28.9	1	6	5
6	1092	T24	1	1	1	1	1	#N/B	1	SF	28.9	1	6	5
6	1092	T36	1	1	1	1	1	#N/B	1	SF	28.9	1	6	5
6	1092	T6	2	1	2	2	2	#N/B	0.62	SF	28.9	1	6	5
6	1089	T12	2	1	3	2	2	#N/B	0.293	SF	40.5	1	11	12
6	1089	T24	2	1	3	1	1	#N/B	0.487	SF	40.5	1	11	12
6	1089	T6	1	1	1	1	2	#N/B	0.848	SF	40.5	1	11	12
6	1086	T6	1	1	1	2	1	#N/B	0.796	SF	41.5	0	7	14
6	1075	T12	1	1	1	2	3	#N/B	0.291	SF	42.5	1	8	5
6	1075	T24	1	1	1	1	2	#N/B	0.848	SF	42.5	1	8	5
6	1075	T6	1	1	1	2	1	#N/B	0.796	SF	42.5	1	2	5
6	1068	T12	1	1	1	2	1	#N/B	0.796	SF	42.2	1	6	12
6	1068	T6	1	1	1	2	2	#N/B	0.725	SF	42.2	1	6	12
6	1065	T24	1	1	1	2	1	#N/B	0.796	SF	29.5	1	24	87
6	1065	T6	1	1	1	2	1	#N/B	0.796	SF	29.5	1	24	87
6	1063	T24	2	1	1	1	1	#N/B	0.85	SF	20.7	1	2	15
6	1062	T12	2	1	1	2	1	#N/B	0.727	SF	51.8	1	21	19
6	1062	T24	1	1	1	2	1	#N/B	0.796	SF	51.8	1	21	19
6	1062	T36	1	1	1	1	1	#N/B	1	SF	51.8	1	21	19

6	1062	T6	1	1	1	2	2	#N/B	0.725	SF	51.8	1	21	19
6	1056	T12	1	1	2	3	1	#N/B	0.228	SF	47.1	1	4	16
6	1056	T6	2	2	2	1	2	#N/B	0.639	SF	47.1	1	4	16
6	1055	T12	1	1	1	1	3	#N/B	0.414	SF	33.4	1	11	10
6	1055	T6	1	1	2	2	2	#N/B	0.689	SF	33.4	1	11	10
6	1054	T12	1	1	1	2	1	#N/B	0.796	SF	63.2	1	11	5
6	1054	T6	1	1	1	2	1	#N/B	0.796	SF	63.2	1	11	5
6	1053	T12	1	2	2	2	1	#N/B	0.656	SF	34.1	1	45	76
6	1053	T24	1	2	2	2	1	#N/B	0.656	SF	34.1	1	45	76
6	1053	T36	1	1	1	2	2	#N/B	0.725	SF	34.1	1	45	76
6	1053	T6	1	1	1	2	1	#N/B	0.796	SF	34.1	1	45	76
6	1047	T12	1	1	1	2	1	#N/B	0.796	SF	44.8	0	14	18
6	1047	T24	1	1	1	2	2	#N/B	0.725	SF	44.8	0	14	18
6	1047	T36	1	1	1	1	1	#N/B	1	SF	44.8	0	14	18
6	1047	T6	2	1	1	2	1	#N/B	0.727	SF	44.8	0	14	18
6	1043	T6	2	3	1	2	3	#N/B	0.008	SF	23.3	1	24	86
6	1039	T6	2	1	2	2	3	#N/B	0.186	SF	25.9	0	10	7
6	1038	T24	1	1	1	2	1	#N/B	0.796	SF	43.7	1	15	21
6	1038	T36	1	1	1	1	1	#N/B	1	SF	43.7	1	15	21
6	1038	T6	1	1	2	2	1	#N/B	0.76	SF	43.7	1	15	21
6	1037	T12	1	1	2	2	1	#N/B	0.76	SF	28.9	1	13	25
6	1037	T24	1	1	2	1	2	#N/B	0.812	SF	28.9	1	13	25
6	1037	T36	1	1	1	2	1	#N/B	0.796	SF	28.9	1	13	25
6	1037	T6	1	1	1	2	2	#N/B	0.725	SF	28.9	1	13	25
6	1034	T12	1	1	1	2	1	#N/B	0.796	SF	33	0	7	12
6	1034	T6	1	1	2	2	2	#N/B	0.689	SF	33	0	7	12
6	1033	T6	1	1	1	2	2	#N/B	0.725	SF	33.3	1	12	17
6	1032	T6	1	1	1	2	3	#N/B	0.291	SF	42.6	0	4	23
6	1030	T12	1	1	1	2	1	#N/B	0.796	SF	43.6	1	21	36
6	1027	T12	1	1	1	2	1	#N/B	0.796	SF	20.5	1	27	18
6	1027	T6	1	1	1	2	2	#N/B	0.725	SF	20.5	1	27	18
6	1020	T12	1	1	1	2	1	#N/B	0.796	SF	40.1	0	1	10
6	1020	T6	2	1	1	2	2	#N/B	0.656	SF	40.1	0	1	10
6	1019	T12	1	1	1	1	2	#N/B	0.848	SF	52.4	1	8	44
6	1019	T36	1	1	1	1	1	#N/B	1	SF	52.4	1	8	44
6	1019	T6	1	1	1	1	2	#N/B	0.848	SF	52.4	1	8	44
6	1018	T12	1	1	1	2	1	#N/B	0.796	SF	52.2	1	18	16
6	1018	T36	1	1	1	2	1	#N/B	0.796	SF	52.2	1	18	16
6	1018	T6	1	1	1	2	2	#N/B	0.725	SF	52.2	1	18	16
6	1016	T6	1	1	1	2	2	#N/B	0.725	SF	43.4	1	17	26
6	1011	T6	1	1	2	3	1	#N/B	0.228	SF	54.9	1	16	73
6	1010	T12	1	1	1	1	2	#N/B	0.848	SF	24.1	1	1	13
6	1010	T36	1	1	1	1	1	#N/B	1	SF	24.1	1	1	13
6	1010	T6	1	1	1	2	3	#N/B	0.291	SF	24.1	1	1	13
6	1007	T6	2	1	1	2	2	#N/B	0.656	SF	71.1	1	7	9
6	1006	T6	1	1	1	2	2	#N/B	0.725	SF	72.6	0	6	17
6	1005	T36	1	3	1	1	1	#N/B	0.436	SF	31.4	1	8	6
6	1004	T12	1	1	1	2	1	#N/B	0.796	SF	39	1	45	111
6	1004	T36	2	1	2	2	2	#N/B	0.62	SF	39	1	45	111
6	1003	T12	1	1	1	1	1	#N/B	1	SF	51.4	0	37	73
6	1003	T36	1	1	1	2	1	#N/B	0.796	SF	51.4	0	37	73

6	1003	T6	2	1	1	2	2	#N/B	0.656	SF	51.4	0	37	73
6	284	T12	3	2	3	2	3	#N/B	-0.221	SF	19.3	1	14	71
6	284	T24	1	1	1	1	2	#N/B	0.848	SF	19.3	1	14	71
6	281	T12	1	1	1	1	2	#N/B	0.848	SF	56.6	0	2	24
6	279	T12	2	1	1	1	2	#N/B	0.779	SF	49.7	0	30	28
6	278	T12	1	1	1	2	1	#N/B	0.796	SF	42.8	0	15	20
6	278	T24	1	1	1	2	1	#N/B	0.796	SF	42.8	0	15	20
6	277	T24	1	1	1	1	2	#N/B	0.848	SF	36	1	3	12
6	277	T36	1	1	1	1	1	#N/B	1	SF	36	1	3	12
6	276	T12	1	1	1	2	1	#N/B	0.796	SF	19.1	1	43	45
6	276	T36	2	1	2	2	1	#N/B	0.691	SF	19.1	1	43	45
6	275	T12	2	1	1	1	2	#N/B	0.779	SF	19.2	1	40	17
6	267	T12	1	1	1	1	2	#N/B	0.848	SF	27.5	0	2	12
6	266	T12	2	1	1	2	2	#N/B	0.656	SF	30.1	0	2	7
6	261	T12	1	1	1	1	2	#N/B	0.848	SF	63.7	1	22	34
6	261	T24	1	1	3	3	1	#N/B	0.17	SF	63.7	1	22	34
6	261	T36	2	1	2	2	1	#N/B	0.691	SF	63.7	1	22	34
6	261	T6	2	3	3	2	3	#N/B	-0.086	SF	63.7	1	22	34
6	260	T12	1	1	1	2	2	#N/B	0.725	SF	63.7	1	21	24
6	260	T24	1	1	1	2	2	#N/B	0.725	SF	63.7	1	21	24
6	260	T36	1	1	1	1	1	#N/B	1	SF	63.7	1	21	24
6	258	T12	1	1	1	1	2	#N/B	0.848	SF	48.8	1	21	17
6	258	T24	1	1	1	1	1	#N/B	1	SF	48.8	1	21	17
6	258	T6	1	1	1	2	2	#N/B	0.725	SF	48.8	1	21	17
6	249	T12	2	1	1	2	2	#N/B	0.656	SF	24.5	0	11	10
6	242	T36	1	1	1	1	1	#N/B	1	SF	40.8	1	18	13
6	242	T6	1	1	1	2	1	#N/B	0.796	SF	40.8	1	2	13
6	240	T24	1	1	1	2	1	#N/B	0.796	SF	49.3	1	18	21
6	239	T6	1	1	1	2	2	#N/B	0.725	SF	40.2	1	31	24
6	237	T24	1	1	1	1	1	#N/B	1	SF	57.8	1	1	7
6	234	T24	1	1	1	2	2	#N/B	0.725	SF	39	1	10	15
6	234	T36	1	1	1	1	1	#N/B	1	SF	39	1	10	15
6	234	T6	2	1	2	2	1	#N/B	0.691	SF	39	1	10	15
6	233	T6	1	1	1	2	1	#N/B	0.796	SF	38	1	13	13
6	231	T24	1	2	1	1	2	#N/B	0.744	SF	21.5	1	5	45
6	231	T36	1	1	1	1	1	#N/B	1	SF	21.5	1	5	45
6	227	T24	2	1	1	2	1	#N/B	0.727	SF	31.6	1	24	44
6	227	T36	1	1	1	2	2	#N/B	0.725	SF	31.6	1	24	54
6	227	T6	1	1	3	2	3	#N/B	0.197	SF	31.6	1	24	19
6	221	T6	1	1	1	2	1	#N/B	0.796	SF	54.2	0	1	63
6	218	T24	2	1	1	1	2	#N/B	0.779	SF	41.7	1	19	19
6	217	T6	3	3	1	2	1	#N/B	-0.001	SF	51.9	0	35	47
6	211	T6	1	1	1	2	1	#N/B	0.796	SF	19.3	1	8	12
6	206	T24	1	1	1	2	2	#N/B	0.725	SF	31.3	1	45	35
6	206	T36	1	1	1	2	1	#N/B	0.796	SF	31.3	1	45	35
6	206	T6	1	1	1	2	2	#N/B	0.725	SF	31.3	1	45	35
6	204	T24	1	1	1	2	3	#N/B	0.291	SF	25.5	0	11	10
6	204	T36	1	1	1	1	1	#N/B	1	SF	25.5	0	6	10
6	204	T6	1	1	1	2	2	#N/B	0.725	SF	25.5	0	8	10
6	201	T24	1	1	2	2	1	#N/B	0.76	SF	47.4	1	25	28
6	201	T36	1	1	1	2	1	#N/B	0.796	SF	47.4	1	5	28

6	201	T6	2	1	2	1	3	#N/B	0.309	SF	47.4	1	4	28
6	200	T6	1	1	1	2	1	#N/B	0.796	SF	20.1	1	1	13
6	197	T6	2	3	1	2	1	#N/B	0.244	SF	30.7	1	22	34
6	196	T24	1	1	1	2	2	#N/B	0.725	SF	38.2	0	1	3
6	196	T36	1	1	1	1	1	#N/B	1	SF	38.2	0	1	3
6	196	T6	2	1	1	2	3	#N/B	0.222	SF	38.2	0	1	3
6	195	T24	2	1	1	2	2	#N/B	0.656	SF	39	1	49	46
6	195	T6	1	1	1	2	3	#N/B	0.291	SF	39	1	49	46
6	190	T6	1	1	2	2	2	#N/B	0.689	SF	48.6	1	4	41
6	189	T24	1	1	1	2	2	#N/B	0.725	SF	44.1	0	25	35
6	189	T36	1	1	1	1	2	#N/B	0.848	SF	44.1	0	25	35
6	189	T6	1	1	1	1	1	#N/B	1	SF	44.1	0	25	35
6	188	T24	1	1	1	2	3	#N/B	0.291	SF	49.3	1	2	15
6	188	T36	1	1	1	1	1	#N/B	1	SF	49.3	1	2	15
6	188	T6	1	1	1	1	2	#N/B	0.848	SF	49.3	1	2	15
6	182	T12	1	1	1	2	2	#N/B	0.725	SF	53.7	1	26	46
6	182	T24	1	1	1	1	3	#N/B	0.414	SF	53.7	1	26	45
6	182	T36	1	1	1	2	1	#N/B	0.796	SF	53.7	1	26	73
6	182	T6	1	1	2	2	1	#N/B	0.76	SF	53.7	1	26	132
6	179	T12	1	1	1	1	2	#N/B	0.848	SF	42.1	1	8	5
6	179	T6	1	1	1	2	2	#N/B	0.725	SF	42.1	1	8	5
6	178	T6	1	1	1	2	1	#N/B	0.796	SF	42.2	0	12	17
6	177	T12	1	1	1	1	3	#N/B	0.414	SF	50.4	1	1	13
6	177	T36	1	1	1	1	1	#N/B	1	SF	50.4	1	1	13
6	177	T6	1	1	1	1	1	#N/B	1	SF	50.4	1	1	13
6	176	T12	1	2	2	1	2	#N/B	0.708	SF	43.1	1	43	42
6	176	T24	1	1	1	2	2	#N/B	0.725	SF	43.1	1	14	42
6	176	T36	1	1	2	2	2	#N/B	0.689	SF	43.1	1	60	42
6	176	T6	1	1	1	2	1	#N/B	0.796	SF	43.1	1	14	42
6	175	T12	1	1	2	2	3	#N/B	0.255	SF	24.6	1	8	44
6	175	T24	1	1	1	2	2	#N/B	0.725	SF	24.6	1	17	44
6	175	T36	1	1	1	2	1	#N/B	0.796	SF	24.6	1	30	44
6	175	T6	1	1	1	2	1	#N/B	0.796	SF	24.6	1	60	44
6	174	T12	3	1	3	2	3	#N/B	-0.117	SF	42.3	1	14	23
6	174	T24	2	3	3	1	3	#N/B	0.037	SF	42.3	1	14	23
6	174	T6	3	1	3	1	2	#N/B	0.171	SF	42.3	1	14	23
6	163	T6	1	1	2	2	2	#N/B	0.689	SF	46.9	1	3	16
6	160	T12	2	1	2	1	3	#N/B	0.309	SF	43.2	1	71	234
6	160	T6	3	1	3	1	2	#N/B	0.171	SF	43.2	1	71	234
6	154	T12	2	1	1	2	1	#N/B	0.727	SF	60.4	0	28	144
6	154	T24	1	1	1	3	1	#N/B	0.264	SF	60.4	0	28	144
6	154	T36	2	1	1	2	2	#N/B	0.656	SF	60.4	0	28	144
6	154	T6	2	1	1	2	2	#N/B	0.656	SF	60.4	0	28	144
6	151	T12	1	1	2	2	1	#N/B	0.76	SF	48.3	0	18	21
6	151	T24	1	1	1	2	1	#N/B	0.796	SF	48.3	0	18	21
6	151	T36	1	1	1	2	1	#N/B	0.796	SF	48.3	0	18	21
6	151	T6	3	1	3	2	3	#N/B	-0.117	SF	48.3	0	18	21
6	147	T6	1	1	1	2	3	#N/B	0.291	SF	38	1	6	10
6	146	T12	1	1	1	2	2	#N/B	0.725	SF	41.5	1	3	29
6	146	T24	2	1	1	2	3	#N/B	0.222	SF	41.5	1	3	29
6	146	T6	1	1	1	1	2	#N/B	0.848	SF	41.5	1	3	29

6	145	T12	1	1	1	2	2	#N/B	0.725	SF	46.8	1	62	81
6	145	T24	1	1	1	1	2	#N/B	0.848	SF	46.8	1	62	81
6	145	T36	2	1	2	2	1	#N/B	0.691	SF	46.8	1	62	81
6	145	T6	1	1	1	2	2	#N/B	0.725	SF	46.8	1	62	81
6	144	T6	1	1	1	2	2	#N/B	0.725	SF	32.8	1	9	17
6	143	T12	2	1	1	2	2	#N/B	0.656	SF	58.1	0	7	7
6	142	T6	1	1	1	2	1	#N/B	0.796	SF	48.6	0	8	27
6	141	T12	1	1	1	1	2	#N/B	0.848	SF	50.9	1	37	19
6	141	T24	1	1	1	1	1	#N/B	1	SF	50.9	1	37	19
6	141	T36	1	1	1	1	1	#N/B	1	SF	50.9	1	37	19
6	141	T6	2	1	1	1	2	#N/B	0.779	SF	50.9	1	37	19
6	140	T36	1	1	1	2	1	#N/B	0.796	SF	48.7	0	34	44
6	139	T12	1	1	1	2	3	#N/B	0.291	SF	45.4	0	5	34
6	139	T24	1	1	1	2	1	#N/B	0.796	SF	45.4	0	31	34
6	139	T36	1	1	1	2	1	#N/B	0.796	SF	45.4	0	13	34
6	139	T6	1	1	1	2	2	#N/B	0.725	SF	45.4	0	3	34
6	138	T12	1	1	1	2	1	#N/B	0.796	SF	39.5	0	19	19
6	137	T12	2	1	1	2	2	#N/B	0.656	SF	20.4	1	2	10
6	137	T24	2	1	1	2	2	#N/B	0.656	SF	20.4	1	2	10
6	137	T36	1	1	1	2	1	#N/B	0.796	SF	20.4	1	2	10
6	137	T6	1	1	1	2	3	#N/B	0.291	SF	20.4	1	2	10
6	136	T12	1	1	1	2	2	#N/B	0.725	SF	18.7	1	5	14
6	134	T12	1	1	1	2	2	#N/B	0.725	SF	32.1	1	15	23
6	134	T36	1	1	1	1	1	#N/B	1	SF	32.1	1	15	23
6	134	T6	1	1	1	2	2	#N/B	0.725	SF	32.1	1	15	23
6	128	T12	1	1	2	2	1	#N/B	0.76	SF	42.1	0	21	19
6	128	T6	1	1	1	2	2	#N/B	0.725	SF	42.1	0	1	19
6	127	T12	1	1	1	2	2	#N/B	0.725	SF	27.9	1	4	13
6	124	T12	1	1	1	2	2	#N/B	0.725	SF	35.5	1	11	13
6	122	T12	1	1	1	2	2	#N/B	0.725	SF	25.9	1	12	11
6	118	T6	1	1	2	2	1	#N/B	0.76	SF	51.5	0	4	13
6	117	T12	2	1	1	2	2	#N/B	0.656	SF	29.3	1	9	7
6	117	T36	1	1	1	1	1	#N/B	1	SF	29.3	1	9	7
6	117	T6	1	1	1	2	1	#N/B	0.796	SF	29.3	1	9	7
6	116	T12	1	1	1	2	1	#N/B	0.796	SF	20.7	1	5	11
6	116	T36	1	1	1	2	2	#N/B	0.725	SF	20.7	1	5	11
6	116	T6	1	1	2	2	2	#N/B	0.689	SF	20.7	1	5	11
6	115	T12	1	1	1	2	1	#N/B	0.796	SF	49.4	0	7	9
6	115	T36	1	1	1	1	1	#N/B	1	SF	49.4	0	7	9
6	115	T6	1	1	1	2	1	#N/B	0.796	SF	49.4	0	7	9
6	110	T12	2	1	1	2	1	#N/B	0.727	SF	55.1	1	52	76
6	110	T36	2	1	2	1	2	#N/B	0.743	SF	55.1	1	52	76
6	110	T6	1	1	1	2	1	#N/B	0.796	SF	55.1	1	52	76
6	108	T12	1	1	2	2	2	#N/B	0.689	SF	24.1	0	49	127
6	108	T36	2	1	1	1	1	#N/B	0.85	SF	24.1	0	49	127
6	107	T12	1	1	1	2	1	#N/B	0.796	SF	38.8	0	11	15
6	107	T36	1	1	1	2	1	#N/B	0.796	SF	38.8	0	7	15
6	107	T6	1	1	1	1	2	#N/B	0.848	SF	38.8	0	19	15
6	105	T6	1	1	1	2	1	#N/B	0.796	SF	20.7	1	5	7
6	104	T12	1	1	1	2	1	#N/B	0.796	SF	21.9	1	14	12
6	104	T36	1	1	1	1	1	#N/B	1	SF	21.9	1	14	12

6	104	T6	1	1	1	2	2	#N/B	0.725	SF	21.9	1	14	12
6	103	T12	1	1	1	2	1	#N/B	0.796	SF	30.2	1	13	18
6	103	T36	1	1	1	1	1	#N/B	1	SF	30.2	1	18	18
6	103	T6	1	1	1	2	3	#N/B	0.291	SF	30.2	1	11	18
6	102	T12	1	1	1	2	2	#N/B	0.725	SF	31.9	1	1	3
6	102	T6	1	1	1	2	1	#N/B	0.796	SF	31.9	1	1	3
6	99	T24	1	1	1	2	3	#N/B	0.291	SF	51.8	0	8	21
6	99	T6	1	1	3	2	1	#N/B	0.433	SF	51.8	0	8	21
6	97	T12	2	1	1	2	2	#N/B	0.656	SF	29.1	0	13	7
6	97	T24	1	2	2	2	3	#N/B	0.151	SF	29.1	0	13	7
6	97	T36	2	2	2	3	3	#N/B	-0.181	SF	29.1	0	13	7
6	97	T6	1	1	1	2	2	#N/B	0.725	SF	29.1	0	13	7
6	96	T12	1	1	1	2	1	#N/B	0.796	SF	34.7	1	4	21
6	96	T24	1	1	1	2	2	#N/B	0.725	SF	34.7	1	4	21
6	96	T36	1	1	1	1	1	#N/B	1	SF	34.7	1	4	21
6	96	T6	1	1	1	2	2	#N/B	0.725	SF	34.7	1	4	21
6	95	T12	1	1	1	1	3	#N/B	0.414	SF	24.8	1	8	8
6	95	T24	1	1	1	2	1	#N/B	0.796	SF	24.8	1	8	8
6	95	T36	1	1	1	2	1	#N/B	0.796	SF	24.8	1	8	8
6	95	T6	1	1	1	1	2	#N/B	0.848	SF	24.8	1	8	8
6	94	T12	2	2	2	1	1	#N/B	0.71	SF	42.6	1	9	11
6	94	T24	1	1	2	3	1	#N/B	0.228	SF	42.6	1	9	11
6	94	T36	2	1	2	3	2	#N/B	0.088	SF	42.6	1	9	11
6	94	T6	2	1	1	1	1	#N/B	0.85	SF	42.6	1	9	11
6	93	T12	1	1	1	2	1	#N/B	0.796	SF	26.6	1	10	18
6	93	T24	1	1	1	2	1	#N/B	0.796	SF	26.6	1	13	18
6	93	T36	2	1	1	1	1	#N/B	0.85	SF	26.6	1	13	18
6	93	T6	1	1	1	1	2	#N/B	0.848	SF	26.6	1	8	18
6	92	T36	1	1	1	1	2	#N/B	0.848	SF	30.4	1	4	8
6	92	T6	1	1	1	2	2	#N/B	0.725	SF	30.4	1	4	8
6	89	T6	1	1	1	2	1	#N/B	0.796	SF	49.9	1	3	38
6	88	T12	1	1	1	2	2	#N/B	0.725	SF	51.1	0	9	16
6	88	T36	1	1	1	1	1	#N/B	1	SF	51.1	0	9	16
6	87	T12	2	1	1	2	2	#N/B	0.656	SF	41	0	8	19
6	87	T24	1	1	1	2	1	#N/B	0.796	SF	41	0	8	19
6	87	T36	1	1	1	1	1	#N/B	1	SF	41	0	8	19
6	87	T6	1	1	1	2	3	#N/B	0.291	SF	41	0	8	19
6	86	T12	1	1	1	1	1	#N/B	1	SF	26.6	1	10	9
6	86	T24	1	1	1	1	2	#N/B	0.848	SF	26.6	1	10	9
6	86	T36	1	1	1	2	2	#N/B	0.725	SF	26.6	1	10	9
6	86	T6	1	1	1	1	2	#N/B	0.848	SF	26.6	1	10	9
6	84	T12	1	1	1	2	2	#N/B	0.725	SF	22.2	0	12	17
6	83	T12	1	1	1	2	3	#N/B	0.291	SF	32.3	0	11	15
6	83	T24	1	1	1	2	1	#N/B	0.796	SF	32.3	0	11	15
6	83	T36	1	1	1	1	1	#N/B	1	SF	32.3	0	11	15
6	83	T6	1	1	2	2	2	#N/B	0.689	SF	32.3	0	11	15
6	82	T12	1	1	2	2	2	#N/B	0.689	SF	47.4	1	68	54
6	82	T24	1	1	2	2	2	#N/B	0.689	SF	47.4	1	68	54
6	82	T36	1	1	1	1	1	#N/B	1	SF	47.4	1	68	54
6	81	T12	1	1	1	2	2	#N/B	0.725	SF	38.9	1	8	18
6	81	T24	1	1	1	1	1	#N/B	1	SF	38.9	1	8	18

6	81	T36	1	1	1	1	1	#N/B	1	SF	38.9	1	8	18
6	81	T6	1	1	1	3	1	#N/B	0.264	SF	38.9	1	8	18
6	79	T6	1	1	1	2	1	#N/B	0.796	SF	61	0	4	16
6	76	T12	1	1	1	2	2	#N/B	0.725	SF	41.4	1	11	8
6	76	T24	1	1	1	2	1	#N/B	0.796	SF	41.4	1	11	8
6	76	T36	1	1	1	1	2	#N/B	0.848	SF	41.4	1	11	8
6	75	T6	1	1	1	2	2	#N/B	0.725	SF	37.9	1	12	19
6	73	T6	1	1	2	2	1	#N/B	0.76	SF	47.2	1	7	7
6	72	T12	3	2	1	2	2	#N/B	0.038	SF	44.7	1	44	37
6	72	T24	1	1	1	2	2	#N/B	0.725	SF	44.7	1	44	37
6	72	T6	1	1	1	2	1	#N/B	0.796	SF	44.7	1	44	37
6	69	T12	1	1	1	2	3	#N/B	0.291	SF	39.8	1	15	13
6	69	T6	1	1	1	2	2	#N/B	0.725	SF	39.8	1	15	13
6	68	T12	1	1	1	2	1	#N/B	0.796	SF	32.3	1	13	20
6	68	T24	1	1	1	2	2	#N/B	0.725	SF	32.3	1	13	20
6	68	T36	1	1	1	1	1	#N/B	1	SF	32.3	1	13	20
6	68	T6	1	1	1	2	2	#N/B	0.725	SF	32.3	1	13	20
6	67	T12	1	1	2	2	2	#N/B	0.689	SF	36.5	1	10	29
6	67	T36	2	1	2	2	2	#N/B	0.62	SF	36.5	1	10	29
6	66	T12	1	1	1	2	1	#N/B	0.796	SF	18.2	0	4	2
6	66	T24	1	1	1	2	2	#N/B	0.725	SF	18.2	0	4	2
6	64	T12	1	1	1	2	1	#N/B	0.796	SF	50.6	1	11	11
6	64	T24	1	1	2	2	2	#N/B	0.689	SF	50.6	1	11	11
6	64	T6	1	1	1	2	2	#N/B	0.725	SF	50.6	1	11	11
6	61	T12	1	1	1	2	1	#N/B	0.796	SF	46.2	1	23	39
6	61	T24	1	1	1	2	3	#N/B	0.291	SF	46.2	1	23	39
6	61	T36	1	1	1	1	2	#N/B	0.848	SF	46.2	1	23	39
6	61	T6	2	1	1	2	2	#N/B	0.656	SF	46.2	1	23	39
6	60	T6	1	1	1	2	2	#N/B	0.725	SF	41.9	1	55	121
6	59	T12	1	1	1	1	2	#N/B	0.848	SF	26.5	1	2	5
6	59	T24	1	1	1	2	1	#N/B	0.796	SF	26.5	1	2	5
6	59	T6	1	1	1	1	2	#N/B	0.848	SF	26.5	1	2	5
6	58	T12	2	1	2	2	1	#N/B	0.691	SF	45.3	1	25	22
6	58	T24	1	1	1	2	1	#N/B	0.796	SF	45.3	1	25	26
6	58	T36	1	1	2	2	2	#N/B	0.689	SF	45.3	1	25	31
6	58	T6	1	1	1	2	2	#N/B	0.725	SF	45.3	1	25	26
6	57	T6	1	1	1	2	3	#N/B	0.291	SF	25.5	1	21	11
6	56	T12	1	1	1	2	1	#N/B	0.796	SF	54.7	1	14	42
6	56	T24	1	1	1	2	1	#N/B	0.796	SF	54.7	1	14	42
6	56	T36	1	1	1	1	1	#N/B	1	SF	54.7	1	14	42
6	56	T6	1	1	1	2	2	#N/B	0.725	SF	54.7	1	14	42
6	55	T12	1	1	2	2	1	#N/B	0.76	SF	41	1	45	73
6	55	T24	1	1	2	2	1	#N/B	0.76	SF	41	1	45	73
6	55	T36	2	1	1	1	1	#N/B	0.85	SF	41	1	45	73
6	55	T6	1	1	2	2	2	#N/B	0.689	SF	41	1	45	73
6	54	T12	1	1	1	2	1	#N/B	0.796	SF	28.6	0	6	17
6	54	T24	1	1	1	2	2	#N/B	0.725	SF	28.6	0	6	17
6	54	T36	1	1	1	1	1	#N/B	1	SF	28.6	0	6	17
6	54	T6	1	1	1	2	2	#N/B	0.725	SF	28.6	0	6	17
6	53	T12	2	1	1	2	1	#N/B	0.727	SF	55.3	0	3	15
6	53	T24	3	3	3	1	1	#N/B	0.028	SF	55.3	0	3	15

6	53	T6	1	1	2	2	2	#N/B	0.689	SF	55.3	0	3	15
6	50	T12	2	1	1	2	1	#N/B	0.727	SF	34.5	1	15	21
6	49	T12	1	1	1	2	1	#N/B	0.796	SF	42.2	0	5	12
6	49	T24	1	1	1	2	1	#N/B	0.796	SF	42.2	0	5	6
6	49	T36	1	1	1	1	1	#N/B	1	SF	42.2	0	11	19
6	49	T6	1	1	2	2	2	#N/B	0.689	SF	42.2	0	8	17
6	48	T12	1	1	2	2	1	#N/B	0.76	SF	31.1	1	29	13
6	48	T36	2	1	2	2	2	#N/B	0.62	SF	31.1	1	29	13
6	48	T6	2	1	1	2	3	#N/B	0.222	SF	31.1	1	29	13
6	47	T12	1	1	1	2	1	#N/B	0.796	SF	18.1	1	11	11
6	47	T24	1	1	1	2	2	#N/B	0.725	SF	18.1	1	11	11
6	47	T36	1	1	1	1	1	#N/B	1	SF	18.1	1	11	11
6	47	T6	1	1	1	2	1	#N/B	0.796	SF	18.1	1	11	11
6	46	T6	2	1	1	1	2	#N/B	0.779	SF	28.5	1	15	17
6	41	T12	2	1	1	2	2	#N/B	0.656	SF	37.9	0	12	75
6	41	T24	1	1	1	2	2	#N/B	0.725	SF	37.9	0	12	75
6	40	T12	1	1	1	2	3	#N/B	0.291	SF	50.5	1	4	5
6	40	T24	1	1	1	2	2	#N/B	0.725	SF	50.5	1	4	5
6	40	T6	1	1	1	2	2	#N/B	0.725	SF	50.5	1	4	5
6	38	T6	1	1	1	2	2	#N/B	0.725	SF	33.4	0	24	132
6	37	T12	1	1	1	2	1	#N/B	0.796	SF	34.1	1	20	63
6	37	T24	2	1	1	2	3	#N/B	0.222	SF	34.1	1	20	63
6	37	T36	2	1	2	3	2	#N/B	0.088	SF	34.1	1	20	63
6	37	T6	1	2	1	1	1	#N/B	0.815	SF	34.1	1	20	63
6	32	T12	2	1	1	2	1	#N/B	0.727	SF	56.1	1	13	16
6	32	T24	1	1	1	2	2	#N/B	0.725	SF	56.1	1	13	16
6	32	T36	1	1	1	1	1	#N/B	1	SF	56.1	1	13	16
6	32	T6	1	1	1	2	1	#N/B	0.796	SF	56.1	1	13	16
6	31	T12	2	1	1	2	1	#N/B	0.727	SF	32.2	1	17	12
6	31	T24	1	1	2	2	2	#N/B	0.689	SF	32.2	1	17	12
6	31	T6	1	1	1	2	2	#N/B	0.725	SF	32.2	1	17	12
6	30	T12	1	1	1	2	2	#N/B	0.725	SF	27.7	0	33	54
6	30	T24	1	1	1	2	2	#N/B	0.725	SF	27.7	0	33	54
6	29	T12	1	1	1	2	2	#N/B	0.725	SF	39.8	1	16	16
6	29	T24	1	1	1	1	2	#N/B	0.848	SF	39.8	1	16	16
6	29	T36	1	1	1	1	1	#N/B	1	SF	39.8	1	16	16
6	29	T6	1	1	1	2	2	#N/B	0.725	SF	39.8	1	16	16
6	27	T6	1	1	1	2	2	#N/B	0.725	SF	26.1	1	55	103
6	25	T12	1	1	1	1	2	#N/B	0.848	SF	37.1	1	21	15
6	25	T24	1	1	1	1	1	#N/B	1	SF	37.1	1	21	15
6	25	T36	1	1	1	1	1	#N/B	1	SF	37.1	1	21	15
6	25	T6	1	1	1	2	2	#N/B	0.725	SF	37.1	1	21	15
6	23	T12	2	1	1	2	2	#N/B	0.656	SF	25.7	1	5	9
6	23	T6	1	1	2	2	3	#N/B	0.255	SF	25.7	1	5	9
6	21	T12	1	1	1	2	1	#N/B	0.796	SF	45.5	1	14	45
6	21	T24	1	1	1	2	1	#N/B	0.796	SF	45.5	1	14	45
6	21	T36	1	1	1	2	2	#N/B	0.725	SF	45.5	1	14	45
6	20	T12	1	1	2	1	2	#N/B	0.812	SF	51.8	1	4	11
6	20	T6	1	2	1	2	2	#N/B	0.621	SF	51.8	1	4	11
6	17	T12	1	1	1	2	2	#N/B	0.725	SF	38.7	1	8	84
6	17	T24	2	1	1	3	2	#N/B	0.124	SF	38.7	1	8	84

6	17	T36	1	1	1	1	1	#N/B	1	SF	38.7	1	8	84
6	14	T12	1	1	1	2	2	#N/B	0.725	SF	60.1	0	11	12
6	14	T24	1	1	1	2	2	#N/B	0.725	SF	60.1	0	11	12
6	13	T12	1	1	2	1	2	#N/B	0.812	SF	57.3	0	21	38
6	13	T24	1	3	2	1	2	#N/B	0.329	SF	57.3	0	21	38
6	13	T6	2	1	1	2	2	#N/B	0.656	SF	57.3	0	21	38
6	9	T12	1	1	1	2	3	#N/B	0.291	SF	37.8	0	9	26
6	9	T24	1	1	1	2	3	#N/B	0.291	SF	37.8	0	9	26
6	9	T36	1	1	1	2	1	#N/B	0.796	SF	37.8	0	9	26
6	8	T12	1	1	2	1	2	#N/B	0.812	SF	55.3	0	39	36
6	8	T24	1	1	1	2	2	#N/B	0.725	SF	55.3	0	39	36
6	8	T36	1	1	1	1	1	#N/B	1	SF	55.3	0	39	36
6	7	T12	1	1	1	2	2	#N/B	0.725	SF	43.4	1	47	143
6	7	T24	1	1	1	2	2	#N/B	0.725	SF	43.4	1	47	143
6	7	T36	1	1	1	1	1	#N/B	1	SF	43.4	1	47	143
6	6	T12	1	1	2	2	1	#N/B	0.76	SF	36.6	1	76	111
6	4	T12	2	2	1	2	1	#N/B	0.623	SF	47.5	1	12	31
6	4	T24	2	1	2	2	2	#N/B	0.62	SF	47.5	1	12	31
6	4	T36	1	1	1	2	2	#N/B	0.725	SF	47.5	1	12	31
6	3	T24	1	1	1	2	3	#N/B	0.291	SF	41.3	1	1	8
6	1	T12	1	1	1	1	2	#N/B	0.848	SF	20.2	1	18	16
6	1	T24	1	1	1	2	1	#N/B	0.796	SF	20.2	1	18	16
6	1	T36	1	1	1	1	1	#N/B	1	SF	20.2	1	18	16
7	86	T12	1	1	1	1	1	100	1	EQ	50	0	7	46
7	86	T3	1	1	1	2	1	100	0.796	EQ	50	0	7	46
7	85	T12	2	2	2	2	2	100	0.516	EQ	65	1	19.5	58
7	85	T3	2	3	3	2	1	60	0.15	EQ	65	1	19.5	58
7	84	T12	1	1	1	1	1	90	1	EQ	79	1	0.75	14
7	84	T3	1	1	1	1	1	77	1	EQ	79	1	0.75	14
7	83	T3	1	1	1	1	1	95	1	EQ	22	1	0.5	9
7	82	T12	2	3	3	1	1	60	0.273	EQ	67	1	1.5	39
7	82	T3	2	2	3	2	1	70	0.26	EQ	67	1	1.5	39
7	81	T12	1	2	2	2	1	70	0.656	EQ	45	1	14	26
7	81	T3	1	2	2	2	1	100	0.656	EQ	45	1	14	26
7	80	T12	1	1	1	1	1	100	1	EQ	52	0	1	7
7	80	T3	1	1	1	1	1	100	1	EQ	52	0	1	7
7	79	T12	2	1	2	1	1	100	0.814	EQ	76	0	5	17
7	79	T3	2	1	2	2	1	90	0.691	EQ	76	0	5	17
7	78	T3	1	1	1	2	2	80	0.725	EQ	27	1	9	30
7	77	T12	1	1	1	1	1	85	1	EQ	23	1	21	43
7	77	T3	1	1	1	1	1	80	1	EQ	23	1	21	43
7	76	T12	1	2	2	1	1	75	0.779	EQ	57	0	2	2
7	76	T3	1	2	2	2	1	50	0.656	EQ	57	0	2	2
7	75	T12	2	1	1	2	2	70	0.656	EQ	62	0	16	29
7	75	T3	2	1	2	2	2	40	0.62	EQ	62	0	16	29
7	74	T12	1	1	1	2	1	71	0.796	EQ	29	1	25	2
7	74	T3	1	1	2	2	1	70	0.76	EQ	29	1	25	2
7	73	T12	2	1	2	2	2	100	0.62	EQ	22	0	6	20
7	73	T3	2	2	2	2	2	60	0.516	EQ	22	0	6	20
7	72	T12	1	2	2	1	1	80	0.779	EQ	66	1	3	20
7	72	T3	1	2	2	2	1	70	0.656	EQ	66	1	3	20

7	71	T12	1	1	1	1	1	80	1	EQ	21	1	15	24
7	71	T3	1	1	1	1	1	70	1	EQ	21	1	15	24
7	69	T12	1	1	1	1	1	100	1	EQ	46	1	1	7
7	69	T3	1	1	1	2	1	80	0.796	EQ	46	1	1	7
7	68	T12	2	2	2	2	1	54	0.587	EQ	57	1	6	46
7	68	T3	2	1	1	2	1	90	0.727	EQ	57	1	6	46
7	67	T12	1	1	2	1	1	100	0.883	EQ	55	1	7	39
7	67	T3	1	1	1	2	1	80	0.796	EQ	55	1	7	39
7	66	T12	2	1	2	2	3	60	0.186	EQ	72	0	5	21
7	66	T3	2	1	2	2	3	50	0.186	EQ	72	0	5	21
7	65	T12	1	1	1	1	1	52	1	EQ	41	1	5	30
7	65	T3	1	1	1	1	2	70	0.848	EQ	41	1	5	30
7	64	T12	1	1	1	1	2	90	0.848	EQ	47	1	28	51
7	64	T3	1	1	2	2	1	80	0.76	EQ	47	1	28	51
7	63	T12	2	1	1	1	1	100	0.85	EQ	56	1	1.5	12
7	63	T3	1	1	1	1	1	80	1	EQ	56	1	1.5	12
7	62	T12	1	1	1	1	1	90	1	EQ	65	1	30.5	42
7	62	T3	1	2	2	1	1	95	0.779	EQ	65	1	30.5	42
7	61	T12	1	1	2	2	1	90	0.76	EQ	18	1	6	19
7	61	T3	2	1	2	2	1	78	0.691	EQ	18	1	6	19
7	60	T12	1	1	2	1	1	70	0.883	EQ	35	1	19	45
7	60	T3	1	1	3	1	1	80	0.556	EQ	35	1	19	45
7	59	T12	1	1	2	3	1	60	0.228	EQ	44	0	20	24
7	59	T3	2	2	2	2	2	40	0.516	EQ	44	0	20	24
7	57	T12	1	2	2	1	2	70	0.708	EQ	81	0	5	43
7	57	T3	1	2	2	1	1	50	0.779	EQ	81	0	5	43
7	56	T12	1	1	1	1	2	80	0.848	EQ	42	1	31	71
7	54	T12	1	1	1	1	1	95	1	EQ	36	0	0.5	0
7	54	T3	1	1	1	1	1	90	1	EQ	36	0	0.5	0
7	53	T12	1	1	1	1	1	80	1	EQ	41	1	0.5	4
7	53	T3	1	1	1	1	1	90	1	EQ	41	1	0.5	4
7	52	T3	1	1	2	1	1	85	0.883	EQ	51	0	11.5	25
7	51	T12	1	1	2	2	3	92	0.255	EQ	20	1	7.5	21
7	51	T3	1	1	3	1	2	55	0.485	EQ	20	1	7.5	21
7	50	T3	1	1	1	1	1	78	1	EQ	72	1	2	16
7	49	T3	1	1	1	1	1	#N/B	1	EQ	43	1	6	16
7	48	T12	1	1	1	2	2	80	0.725	EQ	38	0	2.5	6
7	48	T3	1	1	1	2	1	70	0.796	EQ	38	0	2.5	6
7	47	T12	1	1	1	1	1	80	1	EQ	40	0	1.5	5
7	47	T3	1	1	1	2	1	90	0.796	EQ	40	0	1.5	5
7	46	T3	1	1	2	2	1	75	0.76	EQ	41	1	3	9
7	45	T12	1	1	1	1	1	70	1	EQ	59	0	2	4
7	45	T3	1	1	1	1	1	70	1	EQ	59	0	2	4
7	44	T12	1	1	1	2	1	70	0.796	EQ	57	1	0.2	0
7	43	T12	1	1	1	1	1	94	1	EQ	64	1	4.5	31
7	40	T12	2	1	2	2	2	73	0.62	EQ	61	1	4	7
7	40	T3	2	1	1	2	1	90	0.727	EQ	61	1	4	7
7	38	T12	1	1	2	2	1	70	0.76	EQ	29	0	5	20
7	36	T3	2	1	2	1	1	95	0.814	EQ	58	1	1.5	6
7	35	T3	2	1	2	3	1	60	0.159	EQ	36	1	19	10
7	34	T3	1	1	1	1	1	90	1	EQ	57	1	4	4

7	33	T12	1	2	2	2	3	30	0.151	EQ	50	0	8	34
7	33	T3	1	2	2	2	3	40	0.151	EQ	50	0	8	34
7	32	T3	2	1	3	2	2	40	0.293	EQ	34	1	13.5	16
7	31	T12	1	1	1	1	1	90	1	EQ	56	0	0.5	4
7	31	T3	1	1	1	2	1	90	0.796	EQ	56	0	0.5	4
7	30	T3	2	1	2	2	1	80	0.691	EQ	34	0	1	0
7	29	T12	1	1	1	1	1	90	1	EQ	23	1	8	8
7	29	T3	1	1	2	1	1	95	0.883	EQ	23	1	8	8
7	27	T12	1	1	1	1	1	70	1	EQ	59	0	13	28
7	27	T3	1	1	2	2	2	70	0.689	EQ	59	0	13	28
7	25	T12	1	1	1	1	1	80	1	EQ	61	0	6	5
7	25	T3	1	1	1	2	2	80	0.725	EQ	61	0	6	5
7	24	T3	1	1	1	1	1	70	1	EQ	70	1	7	22
7	22	T3	1	1	1	2	2	80	0.725	EQ	20	1	7.5	17
7	20	T12	1	1	1	1	1	100	1	EQ	34	0	3	1
7	20	T3	1	1	1	1	1	80	1	EQ	34	0	3	1
7	18	T12	1	1	1	1	1	85	1	EQ	60	1	3	18
7	18	T3	1	1	1	2	2	#N/B	0.725	EQ	60	1	3	18
7	17	T12	1	1	1	1	1	80	1	EQ	54	0	10	31
7	17	T3	1	1	3	2	1	80	0.433	EQ	54	0	10	31
7	16	T12	1	2	2	2	1	75	0.656	EQ	54	0	23	34
7	16	T3	1	3	3	1	2	50	0.271	EQ	54	0	23	34
7	15	T12	1	1	1	1	1	50	1	EQ	44	0	7.5	17
7	14	T12	1	1	1	2	1	60	0.796	EQ	63	0	12	21
7	14	T3	1	1	1	2	2	80	0.725	EQ	63	0	12	21
7	13	T12	1	1	2	2	2	60	0.689	EQ	27	0	7	19
7	13	T3	1	1	2	2	2	50	0.689	EQ	27	0	7	19
7	12	T12	1	1	1	1	1	90	1	EQ	34	1	20	19
7	12	T3	1	1	2	2	1	70	0.76	EQ	34	1	20	19
7	11	T12	1	1	1	2	1	80	0.796	EQ	73	0	1	1
7	11	T3	1	1	1	1	1	90	1	EQ	73	0	1	1
7	10	T12	1	1	1	2	1	70	0.796	EQ	21	0	20	32
7	10	T3	1	2	3	2	1	40	0.329	EQ	21	0	20	32
7	9	T3	1	1	1	1	1	70	1	EQ	30	1	0.5	0
7	8	T3	1	1	1	1	1	85	1	EQ	34	1	15	24
7	7	T12	1	1	2	2	1	80	0.76	EQ	54	0	15	26
7	6	T12	1	1	1	1	2	100	0.848	EQ	49	0	1	3
7	5	T3	1	1	2	2	2	67.5	0.689	EQ	26	1	13	24
7	4	T12	1	1	1	1	1	100	1	EQ	38	1	2	5
7	4	T3	1	1	3	2	1	80	0.433	EQ	38	1	2	5
7	3	T12	1	1	2	1	2	60	0.812	EQ	49	0	7.5	21
7	3	T3	1	1	2	1	2	40	0.812	EQ	49	0	7.5	21
7	2	T12	2	2	2	2	1	65	0.587	EQ	67	1	2.5	9
7	2	T3	2	2	2	2	1	70	0.587	EQ	67	1	2.5	9
7	1	T12	2	1	2	2	2	60	0.62	EQ	25	0	10	34
8	260	T0.75	2	3	3	2	2	25	0.079	EQ	18	0	25	48
8	260	T18	1	1	2	2	2	35	0.689	EQ	18	0	25	48
8	260	T3	2	2	2	2	2	80	0.516	EQ	18	0	25	48
8	260	T9	1	2	2	2	2	70	0.585	EQ	18	0	25	48
8	259	T0.75	2	2	3	2	2	75	0.189	EQ	56	1	14	32
8	259	T18	1	1	2	2	1	80	0.76	EQ	56	1	14	32

8	259	T3	2	2	2	2	2	65	0.516	EQ	56	1	14	32
8	259	T9	1	2	2	2	2	80	0.585	EQ	56	1	14	32
8	258	T0.75	2	1	3	2	1	95	0.364	EQ	43	1	10	15
8	258	T18	1	2	1	2	1	90	0.692	EQ	43	1	10	15
8	258	T3	1	2	2	2	1	80	0.656	EQ	43	1	10	15
8	258	T9	1	2	1	2	1	90	0.692	EQ	43	1	10	15
8	257	T0.75	1	2	2	2	2	70	0.585	EQ	36	1	33	41
8	257	T3	1	2	3	2	2	65	0.258	EQ	36	1	33	41
8	257	T9	1	2	3	3	3	58	-0.17	EQ	36	1	33	41
8	256	T0.75	3	3	3	2	2	60	-0.166	EQ	42	1	31	75
8	256	T3	1	2	3	2	2	45	0.258	EQ	42	1	31	75
8	256	T9	1	1	2	2	3	50	0.255	EQ	42	1	31	75
8	255	T0.75	1	2	2	1	1	70	0.779	EQ	46	1	27	42
8	255	T18	1	1	2	2	2	90	0.689	EQ	46	1	27	42
8	255	T3	1	1	2	2	1	80	0.76	EQ	46	1	27	42
8	255	T9	1	1	2	2	1	50	0.76	EQ	46	1	27	42
8	254	T18	2	2	3	2	2	70	0.189	EQ	24	1	50	101
8	254	T3	2	3	3	2	2	65	0.079	EQ	24	1	50	101
8	254	T9	2	3	3	2	2	65	0.079	EQ	24	1	50	101
8	253	T18	2	2	2	2	2	70	0.516	EQ	53	0	37	77
8	253	T9	2	2	2	2	2	65	0.516	EQ	53	0	37	77
8	252	T0.75	3	3	3	2	2	35	-0.166	EQ	36	0	40	139
8	251	T0.75	2	1	3	2	2	80	0.293	EQ	29	1	12	35
8	251	T3	2	1	2	3	2	55	0.088	EQ	29	1	12	35
8	251	T9	2	2	2	2	2	60	0.516	EQ	29	1	12	35
8	250	T0.75	2	2	3	2	3	50	0.024	EQ	42	1	25	44
8	250	T3	2	2	2	2	1	40	0.587	EQ	42	1	25	44
8	250	T9	2	2	3	2	1	60	0.26	EQ	42	1	25	44
8	249	T0.75	3	1	3	2	1	70	0.119	EQ	35	1	22	28
8	249	T3	1	1	1	1	1	75	1	EQ	35	1	22	28
8	249	T9	1	1	1	2	2	80	0.725	EQ	35	1	22	28
8	248	T0.75	2	2	3	2	1	60	0.26	EQ	55	1	33	24
8	248	T3	1	2	2	2	1	75	0.656	EQ	55	1	33	24
8	247	T0.75	2	2	1	1	1	83	0.746	EQ	55	1	15	18
8	247	T18	1	1	1	1	1	95	1	EQ	55	1	15	18
8	247	T3	1	1	1	1	1	90	1	EQ	55	1	15	18
8	247	T9	1	1	1	1	1	90	1	EQ	55	1	15	18
8	246	T0.75	2	2	3	2	2	50	0.189	EQ	23	1	23	28
8	246	T3	1	1	2	2	2	50	0.689	EQ	23	1	23	28
8	246	T9	1	1	2	2	2	80	0.689	EQ	23	1	23	28
8	245	T0.75	2	1	2	2	2	60	0.62	EQ	36	1	13	6
8	245	T3	2	2	2	2	2	50	0.516	EQ	36	1	13	6
8	245	T9	1	2	2	2	2	60	0.585	EQ	36	1	13	6
8	244	T0.75	2	2	2	2	1	75	0.587	EQ	19	1	1	12
8	244	T18	1	1	1	2	2	70	0.725	EQ	19	1	1	12
8	244	T3	1	1	2	2	2	85	0.689	EQ	19	1	1	12
8	244	T9	1	1	1	2	2	60	0.725	EQ	19	1	1	12
8	243	T0.75	2	2	3	1	1	75	0.383	EQ	48	1	43	26
8	243	T18	1	1	1	1	1	93	1	EQ	48	1	43	26
8	243	T3	2	1	1	1	2	75	0.779	EQ	48	1	43	26
8	243	T9	1	1	1	1	1	80	1	EQ	48	1	43	26

8	242	T0.75	1	1	3	1	1	80	0.556	EQ	41	0	21	26
8	242	T18	1	1	1	1	1	90	1	EQ	41	0	21	26
8	242	T3	1	1	1	1	2	70	0.848	EQ	41	0	21	26
8	242	T9	1	1	1	1	2	75	0.848	EQ	41	0	21	26
8	241	T0.75	1	2	3	1	1	80	0.452	EQ	23	0	10	21
8	241	T18	1	1	1	1	1	98	1	EQ	23	0	10	21
8	241	T3	1	1	2	1	1	85	0.883	EQ	23	0	10	21
8	241	T9	1	1	1	1	1	85	1	EQ	23	0	10	21
8	240	T0.75	2	2	1	2	2	50	0.552	EQ	36	1	9	69
8	240	T18	2	2	2	2	2	60	0.516	EQ	36	1	9	69
8	240	T3	2	2	2	3	2	45	-0.016	EQ	36	1	9	69
8	240	T9	2	2	2	2	1	55	0.587	EQ	36	1	9	69
8	239	T0.75	2	2	2	2	2	74	0.516	EQ	53	1	8	21
8	239	T18	1	1	2	2	2	60	0.689	EQ	53	1	8	21
8	239	T3	2	1	2	2	2	70	0.62	EQ	53	1	8	21
8	239	T9	1	1	2	2	2	70	0.689	EQ	53	1	8	21
8	238	T0.75	2	3	3	2	2	60	0.079	EQ	65	0	11.5	35
8	238	T18	1	2	1	2	1	90	0.692	EQ	65	0	11.5	35
8	238	T3	3	3	2	2	1	70	-0.037	EQ	65	0	11.5	35
8	238	T9	1	2	2	2	1	70	0.656	EQ	65	0	11.5	35
8	237	T0.75	1	2	3	1	1	80	0.452	EQ	49	1	17	33
8	237	T3	1	1	2	2	1	80	0.76	EQ	49	1	17	33
8	237	T9	1	1	1	1	1	90	1	EQ	49	1	17	33
8	236	T0.75	2	3	3	1	2	50	0.202	EQ	49	1	25	36
8	236	T3	2	2	2	2	2	69	0.516	EQ	49	1	25	36
8	236	T9	2	1	2	2	2	#N/B	0.62	EQ	49	1	25	36
8	235	T0.75	2	2	2	2	2	73	0.516	EQ	31	1	8.5	29
8	235	T3	2	1	1	2	2	78	0.656	EQ	31	1	8.5	29
8	235	T9	1	1	2	2	2	60	0.689	EQ	31	1	8.5	29
8	234	T0.75	1	2	2	2	2	40	0.585	EQ	33	0	40	60
8	233	T9	2	1	2	2	3	50	0.186	EQ	51	0	25	31
8	232	T0.75	1	1	2	1	1	100	0.883	EQ	22	1	10	21
8	232	T3	1	1	1	1	1	95	1	EQ	22	1	10	21
8	232	T9	1	1	1	1	1	100	1	EQ	22	1	10	21
8	231	T0.75	1	1	2	2	1	35	0.76	EQ	41	1	20	18
8	231	T18	1	1	1	1	2	75	0.848	EQ	41	1	20	18
8	231	T3	1	2	2	2	1	60	0.656	EQ	41	1	20	18
8	231	T9	1	1	1	1	1	80	1	EQ	41	1	20	18
8	230	T18	1	2	1	2	1	88	0.692	EQ	44	1	5	19
8	230	T3	2	1	3	2	1	100	0.364	EQ	44	1	5	19
8	230	T9	2	1	2	2	1	80	0.691	EQ	44	1	5	19
8	229	T0.75	2	2	2	2	2	70	0.516	EQ	40	1	11	16
8	229	T18	1	1	2	2	1	85	0.76	EQ	40	1	11	16
8	229	T3	1	2	2	2	1	60	0.656	EQ	40	1	11	16
8	229	T9	1	2	2	2	2	70	0.585	EQ	40	1	11	16
8	228	T0.75	3	3	3	3	3	40	-0.594	EQ	33	1	35	164
8	228	T18	2	2	2	2	1	78	0.587	EQ	33	1	35	164
8	228	T9	3	3	3	3	2	78	-0.429	EQ	33	1	35	164
8	227	T3	1	1	1	2	1	90	0.796	EQ	18	0	5	4
8	226	T3	1	1	1	1	1	100	1	EQ	38	1	20	24
8	225	T0.75	2	1	3	2	1	70	0.364	EQ	21	1	50	37

8	225	T3	1	1	2	1	1	70	0.883	EQ	21	1	50	37
8	225	T9	1	1	2	1	1	85	0.883	EQ	21	1	50	37
8	224	T0.75	1	2	2	1	1	90	0.779	EQ	36	1	6	14
8	224	T18	1	1	2	2	2	89	0.689	EQ	36	1	6	14
8	224	T3	1	1	3	2	1	84	0.433	EQ	36	1	6	14
8	224	T9	1	2	2	2	2	88	0.585	EQ	36	1	6	14
8	223	T0.75	1	1	1	2	1	90	0.796	EQ	23	1	25	113
8	223	T3	2	3	3	2	1	60	0.15	EQ	23	1	25	113
8	222	T18	1	1	1	1	1	80	1	EQ	44	1	5	10
8	222	T3	2	1	2	2	1	75	0.691	EQ	44	1	5	10
8	222	T9	2	1	2	2	1	60	0.691	EQ	44	1	5	10
8	221	T0.75	1	1	2	2	1	60	0.76	EQ	32	1	40	40
8	221	T18	1	1	2	1	1	100	0.883	EQ	32	1	40	40
8	221	T3	1	1	2	2	2	55	0.689	EQ	32	1	40	40
8	221	T9	1	1	2	1	1	85	0.883	EQ	32	1	40	40
8	220	T0.75	1	1	1	2	2	45	0.725	EQ	25	0	25	27
8	219	T0.75	2	2	3	2	1	60	0.26	EQ	40	1	18	14
8	219	T3	1	1	2	2	1	80	0.76	EQ	40	1	18	14
8	219	T9	1	1	1	1	1	90	1	EQ	40	1	18	14
8	218	T0.75	1	1	2	1	1	#N/B	0.883	EQ	38	1	20	16
8	218	T18	1	1	1	1	1	90	1	EQ	38	1	20	16
8	218	T3	1	1	1	1	1	95	1	EQ	38	1	20	16
8	218	T9	1	1	1	1	1	95	1	EQ	38	1	20	16
8	217	T0.75	1	1	2	2	1	#N/B	0.76	EQ	30	0	2	17
8	217	T18	1	1	1	1	1	95	1	EQ	30	0	2	17
8	217	T3	1	1	2	2	1	85	0.76	EQ	30	0	2	17
8	217	T9	1	1	1	2	1	95	0.796	EQ	30	0	2	17
8	216	T0.75	2	2	3	2	1	70	0.26	EQ	45	1	65	81
8	216	T3	1	1	1	2	1	80	0.796	EQ	45	1	65	81
8	215	T18	1	2	1	2	1	90	0.692	EQ	44	1	60	58
8	215	T3	1	1	2	2	1	80	0.76	EQ	44	1	60	58
8	215	T9	1	2	1	2	1	85	0.692	EQ	44	1	60	58
8	214	T0.75	1	1	1	2	1	95	0.796	EQ	49	0	1	12
8	214	T3	2	1	2	2	2	95	0.62	EQ	49	0	1	12
8	213	T0.75	1	2	2	1	1	80	0.779	EQ	50	1	16	23
8	213	T3	1	2	2	2	2	60	0.585	EQ	50	1	16	23
8	213	T9	1	1	2	2	1	78	0.76	EQ	50	1	16	23
8	212	T0.75	2	2	3	2	2	40	0.189	EQ	18	0	18	50
8	212	T3	2	2	2	2	3	40	0.082	EQ	18	0	18	50
8	212	T9	1	1	2	2	2	75	0.689	EQ	18	0	18	50
8	211	T0.75	3	1	3	2	2	40	0.048	EQ	43	1	18	27
8	211	T3	3	1	3	2	2	42	0.048	EQ	43	1	18	27
8	210	T0.75	1	1	2	1	1	64	0.883	EQ	42	1	8	24
8	210	T3	1	1	2	2	1	58	0.76	EQ	42	1	8	24
8	209	T0.75	1	1	1	2	2	75	0.725	EQ	51	0	10	37
8	209	T18	1	1	1	2	1	75	0.796	EQ	51	0	10	37
8	209	T3	1	1	2	1	1	70	0.883	EQ	51	0	10	37
8	209	T9	1	1	1	2	2	70	0.725	EQ	51	0	10	37
8	208	T0.75	2	3	3	2	2	60	0.079	EQ	53	1	18	56
8	208	T18	1	1	1	1	1	80	1	EQ	53	1	18	56
8	208	T3	1	1	1	1	1	90	1	EQ	53	1	18	56

8	208	T9	1	1	1	1	1	100	1	EQ	53	1	18	56
8	207	T0.75	2	2	2	2	1	65	0.587	EQ	27	1	27	107
8	207	T18	1	1	1	2	1	80	0.796	EQ	27	1	27	107
8	207	T3	2	2	2	2	1	65	0.587	EQ	27	1	27	107
8	207	T9	2	2	2	1	1	75	0.71	EQ	27	1	27	107
8	206	T0.75	2	2	2	2	3	60	0.082	EQ	39	0	38	150
8	205	T0.75	2	2	2	3	2	40	-0.016	EQ	20	0	14	36
8	205	T18	1	1	1	1	1	85	1	EQ	20	0	14	36
8	205	T3	1	1	2	2	2	70	0.689	EQ	20	0	14	36
8	205	T9	1	1	2	2	1	70	0.76	EQ	20	0	14	36
8	202	T0.75	2	2	3	2	1	50	0.26	EQ	50	1	60	58
8	202	T18	1	1	2	2	1	85	0.76	EQ	50	1	60	58
8	202	T3	2	2	3	1	1	50	0.383	EQ	50	1	60	58
8	202	T9	2	1	2	2	1	65	0.691	EQ	50	1	60	58
8	201	T0.75	1	2	1	2	2	85	0.621	EQ	21	0	8	45
8	201	T18	1	1	1	1	1	90	1	EQ	21	0	8	45
8	201	T3	1	2	2	2	2	85	0.585	EQ	21	0	8	45
8	201	T9	1	1	2	2	1	90	0.76	EQ	21	0	8	45
8	200	T0.75	2	3	3	3	2	30	-0.184	EQ	53	0	31	71
8	200	T18	2	3	3	3	2	60	-0.184	EQ	53	0	31	71
8	200	T3	2	3	3	2	2	50	0.079	EQ	53	0	31	71
8	200	T9	2	3	3	3	2	20	-0.184	EQ	53	0	31	71
8	199	T0.75	1	2	2	3	1	60	0.124	EQ	49	1	6	37
8	199	T3	1	1	1	2	1	70	0.796	EQ	49	1	6	37
8	199	T9	1	1	1	2	1	90	0.796	EQ	49	1	6	37
8	198	T0.75	2	2	3	1	1	65	0.383	EQ	55	0	45	46
8	198	T3	2	1	1	2	1	80	0.727	EQ	55	0	45	46
8	197	T0.75	2	2	3	2	1	50	0.26	EQ	35	1	25	37
8	197	T18	1	1	2	2	2	70	0.689	EQ	35	1	25	37
8	197	T3	2	2	3	2	3	65	0.024	EQ	35	1	25	37
8	197	T9	2	2	2	2	2	70	0.516	EQ	35	1	25	37
8	196	T0.75	2	2	2	2	2	#N/B	0.516	EQ	38	0	25	23
8	196	T18	1	1	1	2	1	98	0.796	EQ	38	0	25	23
8	196	T3	1	2	2	2	1	60	0.656	EQ	38	0	25	23
8	196	T9	1	1	2	2	2	75	0.689	EQ	38	0	25	23
8	195	T0.75	2	2	2	2	1	69	0.587	EQ	18	1	19	46
8	195	T18	1	1	1	1	1	85	1	EQ	18	1	19	46
8	195	T3	2	2	1	2	1	75	0.623	EQ	18	1	19	46
8	195	T9	1	1	1	1	1	80	1	EQ	18	1	19	46
8	194	T0.75	2	2	2	3	2	85	-0.016	EQ	53	1	18	37
8	194	T18	1	1	2	2	1	75	0.76	EQ	53	1	18	37
8	194	T3	1	2	2	1	1	70	0.779	EQ	53	1	18	37
8	194	T9	1	2	2	1	2	66	0.708	EQ	53	1	18	37
8	193	T0.75	1	2	3	2	1	73	0.329	EQ	57	0	3.5	24
8	193	T18	1	1	1	1	1	85	1	EQ	57	0	3.5	24
8	193	T3	1	1	2	2	1	74	0.76	EQ	57	0	3.5	24
8	193	T9	1	1	2	2	1	70	0.76	EQ	57	0	3.5	24
8	192	T0.75	2	2	2	1	2	55	0.639	EQ	33	0	24.5	89
8	192	T18	1	1	2	1	1	70	0.883	EQ	33	0	24.5	89
8	192	T3	1	1	2	1	1	70	0.883	EQ	33	0	24.5	89
8	192	T9	1	1	1	1	1	70	1	EQ	33	0	24.5	89

8	191	T0.75	2	2	2	2	1	78	0.587	EQ	65	1	12	38
8	191	T18	1	1	1	1	1	98	1	EQ	65	1	12	38
8	191	T3	1	1	1	1	1	97	1	EQ	65	1	12	38
8	191	T9	1	1	1	1	1	98	1	EQ	65	1	12	38
8	190	T0.75	1	2	3	2	2	70	0.258	EQ	25	1	4.25	45
8	189	T0.75	2	2	3	2	2	40	0.189	EQ	30	1	14	44
8	189	T18	1	2	2	2	1	85	0.656	EQ	30	1	14	44
8	189	T3	1	2	2	2	2	78	0.585	EQ	30	1	14	44
8	189	T9	1	1	1	2	1	78	0.796	EQ	30	1	14	44
8	188	T0.75	2	2	2	2	1	62	0.587	EQ	47	0	24	57
8	188	T18	1	1	2	2	2	52	0.689	EQ	47	0	24	57
8	188	T3	1	2	2	3	2	#N/B	0.053	EQ	47	0	24	57
8	188	T9	1	1	2	2	2	62	0.689	EQ	47	0	24	57
8	187	T0.75	1	2	2	2	2	70	0.585	EQ	64	0	15.75	38
8	187	T18	1	1	1	1	1	85	1	EQ	64	0	15.75	38
8	187	T3	1	1	1	2	2	80	0.725	EQ	64	0	15.75	38
8	187	T9	1	1	1	2	2	77	0.725	EQ	64	0	15.75	38
8	186	T0.75	3	3	3	3	2	20	-0.429	EQ	54	0	50.5	67
8	186	T18	1	1	2	2	2	70	0.689	EQ	54	0	50.5	67
8	186	T3	2	2	2	2	1	76	0.587	EQ	54	0	50.5	67
8	186	T9	1	1	2	2	1	70	0.76	EQ	54	0	50.5	67
8	185	T0.75	2	1	2	2	1	90	0.691	EQ	75	1	5	6
8	185	T3	2	1	1	2	1	90	0.727	EQ	75	1	5	6
8	185	T9	1	1	1	1	1	90	1	EQ	75	1	5	6
8	184	T0.75	1	2	3	2	2	78	0.258	EQ	25	1	8	25
8	184	T3	1	1	3	2	2	78	0.362	EQ	25	1	8	25
8	184	T9	1	1	1	1	2	78	0.848	EQ	25	1	8	25
8	183	T0.75	1	3	3	3	1	65	-0.044	EQ	34	0	15	25
8	183	T18	1	1	1	2	1	91	0.796	EQ	34	0	15	25
8	183	T3	1	1	2	2	1	70	0.76	EQ	34	0	15	25
8	183	T9	1	1	2	2	1	90	0.76	EQ	34	0	15	25
8	182	T0.75	2	2	2	2	2	60	0.516	EQ	41	1	24	34
8	182	T18	1	1	1	1	1	90	1	EQ	41	1	24	34
8	182	T3	1	1	1	2	1	80	0.796	EQ	41	1	24	34
8	182	T9	1	1	1	1	1	85	1	EQ	41	1	24	34
8	181	T0.75	3	2	3	2	1	65	0.015	EQ	37	1	21	21
8	181	T18	1	1	1	1	1	100	1	EQ	37	1	21	21
8	181	T3	2	1	3	2	1	65	0.364	EQ	37	1	21	21
8	181	T9	1	1	2	1	1	95	0.883	EQ	37	1	21	21
8	180	T0.75	1	1	1	1	1	60	1	EQ	60	1	12	24
8	180	T18	1	1	1	1	1	78	1	EQ	60	1	12	24
8	180	T3	1	1	1	1	1	60	1	EQ	60	1	12	24
8	180	T9	1	1	1	1	1	85	1	EQ	60	1	12	24
8	179	T0.75	1	1	1	1	1	70	1	EQ	20	1	21	30
8	179	T3	1	1	1	1	1	85	1	EQ	20	1	21	30
8	178	T0.75	1	1	2	1	1	87	0.883	EQ	55	0	15	17
8	178	T3	1	1	1	1	1	92	1	EQ	55	0	15	17
8	177	T18	1	1	2	2	2	70	0.689	EQ	25	1	20	29
8	177	T9	1	2	2	3	3	52	-0.112	EQ	25	1	20	29
8	176	T0.75	1	1	2	2	1	90	0.76	EQ	42	1	5	86
8	176	T3	1	1	2	2	2	70	0.689	EQ	42	1	5	86

8	175	T0.75	1	1	1	2	1	#N/B	0.796	EQ	19	1	2	8
8	175	T3	1	1	1	1	1	85	1	EQ	19	1	2	8
8	175	T9	1	1	1	1	1	95	1	EQ	19	1	2	8
8	174	T0.75	1	1	1	2	1	80	0.796	EQ	50	1	6	10
8	173	T0.75	2	2	2	2	1	80	0.587	EQ	35	1	4	9
8	173	T3	1	1	1	1	1	85	1	EQ	35	1	4	9
8	173	T9	1	1	1	1	1	85	1	EQ	35	1	4	9
8	172	T0.75	2	2	2	2	1	75	0.587	EQ	52	1	10	6
8	171	T0.75	1	1	2	1	1	50	0.883	EQ	72	1	4	23
8	171	T18	1	1	1	1	1	100	1	EQ	72	1	4	23
8	171	T9	2	2	2	2	3	40	0.082	EQ	72	1	4	23
8	170	T0.75	1	1	1	2	2	88	0.725	EQ	37	1	15	6
8	169	T0.75	1	1	1	1	1	70	1	EQ	57	1	1	10
8	169	T18	1	1	1	1	1	80	1	EQ	57	1	1	10
8	169	T3	1	1	1	1	1	75	1	EQ	57	1	1	10
8	169	T9	1	1	1	1	1	70	1	EQ	57	1	1	10
8	168	T3	1	1	2	1	2	70	0.812	EQ	49	1	5	7
8	168	T9	1	2	2	2	1	80	0.656	EQ	49	1	5	7
8	167	T0.75	2	1	2	2	2	80	0.62	EQ	42	1	7	7
8	167	T18	1	1	1	1	1	98	1	EQ	42	1	7	7
8	167	T3	1	1	1	1	1	90	1	EQ	42	1	7	7
8	167	T9	1	1	1	1	1	100	1	EQ	42	1	7	7
8	166	T3	1	1	1	1	1	95	1	EQ	49	1	3	3
8	165	T0.75	1	1	1	1	1	90	1	EQ	44	1	2	7
8	164	T3	1	2	2	2	1	70	0.656	EQ	32	0	4	10
8	164	T9	1	1	2	2	1	80	0.76	EQ	32	0	4	10
8	163	T0.75	1	1	3	2	1	60	0.433	EQ	43	1	4	8
8	163	T18	1	1	1	1	1	100	1	EQ	43	1	4	8
8	163	T3	1	1	1	1	1	95	1	EQ	43	1	4	8
8	163	T9	1	1	1	1	1	97	1	EQ	43	1	4	8
8	162	T0.75	2	1	3	2	1	60	0.364	EQ	31	1	3	9
8	162	T18	1	1	1	1	1	80	1	EQ	31	1	3	9
8	162	T3	2	1	2	2	1	70	0.691	EQ	31	1	3	9
8	161	T0.75	2	1	2	2	1	90	0.691	EQ	34	1	3	7
8	161	T18	1	1	1	1	1	100	1	EQ	34	1	3	7
8	161	T3	1	1	1	1	1	99	1	EQ	34	1	3	7
8	161	T9	1	1	1	1	1	100	1	EQ	34	1	3	7
8	160	T18	1	1	1	1	1	97	1	EQ	23	1	6	8
8	160	T3	1	1	2	1	1	84	0.883	EQ	23	1	6	8
8	160	T9	1	1	1	2	1	95	0.796	EQ	23	1	6	8
8	159	T0.75	2	2	3	2	2	60	0.189	EQ	29	1	3	7
8	158	T0.75	1	1	1	1	1	80	1	EQ	42	1	1	8
8	158	T18	1	1	1	1	1	83	1	EQ	42	1	1	8
8	158	T3	1	1	1	1	1	90	1	EQ	42	1	1	8
8	158	T9	1	1	1	1	1	80	1	EQ	42	1	1	8
8	157	T0.75	2	1	2	2	1	75	0.691	EQ	42	1	4	4
8	157	T18	1	1	1	1	1	85	1	EQ	42	1	4	4
8	157	T3	1	1	1	1	1	80	1	EQ	42	1	4	4
8	157	T9	1	1	1	1	1	80	1	EQ	42	1	4	4
8	156	T0.75	2	1	2	2	1	65	0.691	EQ	34	1	2	13
8	156	T3	1	1	1	2	2	90	0.725	EQ	34	1	2	13

8	155	T0.75	2	2	2	2	1	90	0.587	EQ	52	1	3	9
8	155	T18	1	1	2	2	1	90	0.76	EQ	52	1	3	9
8	155	T3	1	1	2	2	1	90	0.76	EQ	52	1	3	9
8	155	T9	1	1	1	2	1	90	0.796	EQ	52	1	3	9
8	154	T0.75	1	1	1	1	2	88	0.848	EQ	47	0	5	5
8	153	T0.75	2	2	2	3	1	60	0.055	EQ	68	0	8	35
8	153	T3	1	1	2	1	1	90	0.883	EQ	68	0	8	35
8	151	T0.75	1	1	1	1	2	95	0.848	EQ	45	1	11	9
8	150	T0.75	1	1	2	2	1	27	0.76	EQ	24	0	6	7
8	150	T18	1	1	1	1	1	60	1	EQ	24	0	6	7
8	150	T3	1	1	1	1	1	65	1	EQ	24	0	6	7
8	150	T9	1	1	1	1	1	70	1	EQ	24	0	6	7
8	149	T0.75	1	1	1	1	1	75	1	EQ	26	1	2	14
8	149	T3	1	1	1	2	1	85	0.796	EQ	26	1	2	14
8	148	T0.75	1	1	1	2	1	75	0.796	EQ	33	1	4	7
8	148	T18	1	1	1	2	1	90	0.796	EQ	33	1	4	7
8	148	T3	1	1	1	2	1	75	0.796	EQ	33	1	4	7
8	147	T0.75	1	1	2	2	1	#N/B	0.76	EQ	27	1	2	7
8	147	T3	1	1	2	2	1	78	0.76	EQ	27	1	2	7
8	147	T9	1	1	1	1	1	80	1	EQ	27	1	2	7
8	146	T0.75	2	1	2	2	1	90	0.691	EQ	28	1	21	30
8	146	T3	1	1	2	2	2	100	0.689	EQ	28	1	21	30
8	145	T0.75	1	1	1	1	1	98	1	EQ	22	1	6	24
8	144	T0.75	1	1	1	1	1	75	1	EQ	21	1	12	23
8	144	T3	1	1	1	1	1	80	1	EQ	21	1	12	23
8	143	T0.75	2	1	3	1	2	60	0.416	EQ	58	0	2	19
8	143	T3	2	1	1	2	1	60	0.727	EQ	58	0	2	19
8	142	T0.75	2	2	2	2	2	55	0.516	EQ	18	1	49	44
8	142	T3	1	1	1	1	1	75	1	EQ	18	1	49	44
8	141	T0.75	1	1	2	2	1	60	0.76	EQ	25	1	23	40
8	140	T0.75	1	1	2	2	1	75	0.76	EQ	19	1	6	23
8	140	T3	1	1	2	2	1	80	0.76	EQ	19	1	6	23
8	139	T0.75	2	2	3	3	3	#N/B	-0.239	EQ	44	1	4	20
8	138	T0.75	1	1	1	2	2	80	0.725	EQ	23	1	16	29
8	138	T3	1	1	1	2	2	80	0.725	EQ	23	1	16	29
8	138	T9	1	1	1	2	2	70	0.725	EQ	23	1	16	29
8	137	T0.75	2	1	2	2	3	60	0.186	EQ	28	0	15	29
8	137	T18	1	1	1	2	2	80	0.725	EQ	28	0	15	29
8	137	T3	2	1	2	2	3	80	0.186	EQ	28	0	15	29
8	137	T9	1	1	1	2	2	76	0.725	EQ	28	0	15	29
8	136	T0.75	2	1	2	1	1	85	0.814	EQ	36	1	10	21
8	136	T3	1	1	1	1	1	75	1	EQ	36	1	10	21
8	135	T0.75	2	1	2	2	2	50	0.62	EQ	42	0	12	25
8	135	T18	2	2	2	3	3	80	-0.181	EQ	42	0	12	25
8	135	T3	1	1	2	2	2	70	0.689	EQ	42	0	12	25
8	135	T9	1	1	1	2	2	80	0.725	EQ	42	0	12	25
8	134	T0.75	1	1	2	1	2	60	0.812	EQ	25	0	14	27
8	134	T3	1	1	1	2	2	60	0.725	EQ	25	0	14	27
8	134	T9	1	1	1	1	1	90	1	EQ	25	0	14	27
8	133	T0.75	1	1	2	2	2	80	0.689	EQ	44	1	10	25
8	133	T3	1	1	2	2	1	80	0.76	EQ	44	1	10	25

8	132	T0.75	1	1	1	1	1	67	1	EQ	46	0	5	11
8	132	T18	1	1	1	1	1	90	1	EQ	46	0	5	11
8	132	T3	1	1	1	1	1	66	1	EQ	46	0	5	11
8	132	T9	1	1	1	1	1	90	1	EQ	46	0	5	11
8	131	T0.75	1	1	1	1	1	80	1	EQ	24	1	5	14
8	130	T0.75	3	2	3	2	1	60	0.015	EQ	55	1	25	38
8	130	T18	2	1	1	1	1	90	0.85	EQ	55	1	25	38
8	130	T3	1	2	2	2	2	60	0.585	EQ	55	1	25	38
8	130	T9	2	2	2	1	1	70	0.71	EQ	55	1	25	38
8	129	T0.75	1	1	3	2	2	65	0.362	EQ	51	1	20	27
8	129	T3	1	1	1	2	1	87	0.796	EQ	51	1	20	27
8	129	T9	1	1	1	1	3	80	0.414	EQ	51	1	20	27
8	128	T0.75	1	1	1	1	1	70	1	EQ	29	1	6	27
8	128	T18	1	1	1	1	1	#N/B	1	EQ	29	1	6	27
8	128	T3	1	1	1	1	1	100	1	EQ	29	1	6	27
8	128	T9	1	1	1	1	1	#N/B	1	EQ	29	1	6	27
8	127	T0.75	1	2	2	2	1	99	0.656	EQ	57	1	6	26
8	127	T18	1	1	2	1	1	75	0.883	EQ	57	1	6	26
8	127	T3	1	1	3	2	1	85	0.433	EQ	57	1	6	26
8	127	T9	1	1	2	1	1	95	0.883	EQ	57	1	6	26
8	126	T0.75	2	1	2	1	1	95	0.814	EQ	45	1	3	18
8	126	T18	1	1	1	1	1	95	1	EQ	45	1	3	18
8	126	T3	1	1	1	1	1	97	1	EQ	45	1	3	18
8	126	T9	1	1	1	1	1	95	1	EQ	45	1	3	18
8	125	T0.75	1	1	2	2	1	70	0.76	EQ	45	0	3	10
8	125	T18	2	1	1	2	1	68	0.727	EQ	45	0	3	10
8	125	T3	1	1	1	2	1	75	0.796	EQ	45	0	3	10
8	125	T9	1	1	1	2	1	85	0.796	EQ	45	0	3	10
8	124	T0.75	1	1	2	2	2	75	0.689	EQ	24	1	21	24
8	124	T3	1	1	3	2	2	45	0.362	EQ	24	1	21	24
8	124	T9	1	1	2	1	1	70	0.883	EQ	24	1	21	24
8	123	T0.75	1	1	1	1	1	78	1	EQ	46	1	17	52
8	123	T18	1	1	1	1	1	88	1	EQ	46	1	17	52
8	123	T3	1	1	1	1	1	90	1	EQ	46	1	17	52
8	123	T9	1	1	1	1	1	98	1	EQ	46	1	17	52
8	122	T0.75	1	1	2	2	2	70	0.689	EQ	25	0	14	17
8	122	T18	1	1	1	1	1	70	1	EQ	25	0	14	17
8	122	T3	1	1	2	1	1	70	0.883	EQ	25	0	14	17
8	122	T9	1	1	2	1	2	70	0.812	EQ	25	0	14	17
8	120	T0.75	1	1	1	1	1	90	1	EQ	43	1	5	30
8	120	T18	1	1	2	1	1	90	0.883	EQ	43	1	5	30
8	120	T3	1	1	1	2	2	90	0.725	EQ	43	1	5	30
8	120	T9	1	1	1	2	2	85	0.725	EQ	43	1	5	30
8	119	T0.75	2	1	1	1	1	75	0.85	EQ	39	1	4	26
8	119	T18	1	1	1	1	1	90	1	EQ	39	1	4	26
8	119	T3	1	1	1	1	1	85	1	EQ	39	1	4	26
8	119	T9	1	1	1	1	1	80	1	EQ	39	1	4	26
8	118	T0.75	2	2	2	1	2	20	0.639	EQ	30	0	15	31
8	118	T18	1	1	2	1	2	50	0.812	EQ	30	0	15	31
8	118	T3	1	1	2	1	2	30	0.812	EQ	30	0	15	31
8	118	T9	1	1	2	2	2	40	0.689	EQ	30	0	15	31

8	117	T0.75	1	1	2	2	1	80	0.76	EQ	42	0	13	25
8	117	T18	1	1	1	1	1	89	1	EQ	42	0	13	25
8	117	T3	1	1	1	2	1	80	0.796	EQ	42	0	13	25
8	117	T9	1	1	1	1	2	84	0.848	EQ	42	0	13	25
8	116	T0.75	2	2	3	2	1	60	0.26	EQ	59	1	6	15
8	116	T18	3	3	2	2	3	60	-0.273	EQ	59	1	6	15
8	116	T3	2	1	1	2	2	75	0.656	EQ	59	1	6	15
8	116	T9	2	2	2	1	2	70	0.639	EQ	59	1	6	15
8	115	T0.75	2	2	2	2	2	60	0.516	EQ	48	0	7	14
8	114	T0.75	2	2	2	2	2	70	0.516	EQ	57	1	5	7
8	114	T18	1	1	1	2	1	90	0.796	EQ	57	1	5	7
8	114	T3	1	1	1	1	1	88	1	EQ	57	1	5	7
8	114	T9	1	1	1	2	1	88	0.796	EQ	57	1	5	7
8	113	T0.75	1	1	1	2	2	70	0.725	EQ	57	0	10	29
8	113	T3	1	1	1	2	1	68	0.796	EQ	57	0	10	29
8	113	T9	1	1	1	2	1	75	0.796	EQ	57	0	10	29
8	112	T0.75	1	1	2	1	1	90	0.883	EQ	32	0	25	25
8	112	T18	1	1	1	2	1	#N/B	0.796	EQ	32	0	25	25
8	112	T3	1	1	2	2	1	70	0.76	EQ	32	0	25	25
8	112	T9	1	1	2	2	1	95	0.76	EQ	32	0	25	25
8	111	T0.75	1	1	2	2	1	63	0.76	EQ	47	1	12	26
8	111	T18	1	1	1	1	1	74	1	EQ	47	1	12	26
8	111	T3	1	1	3	2	1	60	0.433	EQ	47	1	12	26
8	111	T9	1	1	2	2	1	70	0.76	EQ	47	1	12	26
8	110	T0.75	1	1	1	1	1	80	1	EQ	33	1	11	17
8	110	T18	1	1	2	1	2	70	0.812	EQ	33	1	11	17
8	110	T3	1	1	1	1	1	90	1	EQ	33	1	11	17
8	110	T9	1	1	1	1	1	70	1	EQ	33	1	11	17
8	109	T0.75	1	1	1	1	2	100	0.848	EQ	42	1	8	20
8	109	T18	1	1	2	2	2	80	0.689	EQ	42	1	8	20
8	109	T3	1	1	2	2	2	75	0.689	EQ	42	1	8	20
8	109	T9	1	1	2	2	1	90	0.76	EQ	42	1	8	20
8	108	T0.75	1	1	2	1	1	100	0.883	EQ	21	1	4	9
8	108	T18	1	1	1	1	1	98	1	EQ	21	1	4	9
8	108	T3	1	1	1	1	1	100	1	EQ	21	1	4	9
8	108	T9	1	1	1	1	1	100	1	EQ	21	1	4	9
8	107	T0.75	2	1	1	1	1	90	0.85	EQ	46	1	12	26
8	106	T0.75	1	1	1	2	1	95	0.796	EQ	29	1	12	10
8	106	T18	1	1	1	1	1	100	1	EQ	29	1	12	10
8	106	T3	1	1	1	2	1	75	0.796	EQ	29	1	12	10
8	106	T9	1	1	1	1	1	95	1	EQ	29	1	12	10
8	105	T0.75	2	2	1	1	1	65	0.746	EQ	18	1	23	22
8	105	T18	1	1	1	1	1	95	1	EQ	18	1	23	22
8	105	T3	1	1	1	1	1	80	1	EQ	18	1	23	22
8	105	T9	1	1	1	1	1	80	1	EQ	18	1	23	22
8	104	T0.75	2	2	2	3	2	65	-0.016	EQ	45	1	15	24
8	104	T18	1	1	1	1	2	68	0.848	EQ	45	1	15	24
8	104	T3	1	2	2	2	3	58	0.151	EQ	45	1	15	24
8	104	T9	1	1	1	2	2	55	0.725	EQ	45	1	15	24
8	103	T0.75	2	1	3	2	1	40	0.364	EQ	38	1	25	21
8	103	T18	1	1	2	2	2	68	0.689	EQ	38	1	25	21

8	103	T3	2	1	2	2	2	58	0.62	EQ	38	1	25	21
8	103	T9	1	1	2	2	2	58	0.689	EQ	38	1	25	21
8	102	T0.75	2	1	2	2	1	75	0.691	EQ	33	0	27	24
8	102	T18	1	1	2	2	1	85	0.76	EQ	33	0	27	24
8	102	T3	1	1	2	2	2	65	0.689	EQ	33	0	27	24
8	102	T9	1	1	2	2	2	80	0.689	EQ	33	0	27	24
8	101	T0.75	1	2	2	2	3	30	0.151	EQ	28	0	17	23
8	101	T18	1	1	2	2	3	40	0.255	EQ	28	0	17	23
8	101	T3	1	1	2	2	3	40	0.255	EQ	28	0	17	23
8	101	T9	1	1	2	2	2	60	0.689	EQ	28	0	17	23
8	100	T0.75	1	1	1	2	1	60	0.796	EQ	23	0	9.5	17
8	100	T3	1	1	2	2	1	55	0.76	EQ	23	0	9.5	17
8	100	T9	1	1	1	2	1	85	0.796	EQ	23	0	9.5	17
8	99	T0.75	2	3	3	3	2	40	-0.184	EQ	35	1	21	22
8	99	T18	2	1	2	2	2	40	0.62	EQ	35	1	21	22
8	99	T3	1	1	2	2	1	40	0.76	EQ	35	1	21	22
8	99	T9	1	1	2	3	2	40	0.157	EQ	35	1	21	22
8	98	T0.75	1	3	3	2	1	75	0.219	EQ	26	1	12.5	21
8	98	T3	1	1	2	1	1	95	0.883	EQ	26	1	12.5	21
8	97	T0.75	1	1	2	1	1	96	0.883	EQ	35	1	0.5	4
8	97	T3	1	1	1	1	1	90	1	EQ	35	1	0.5	4
8	97	T9	1	1	1	1	1	90	1	EQ	35	1	0.5	4
8	96	T0.75	1	2	2	2	1	30	0.656	EQ	44	0	8.5	29
8	96	T18	1	1	1	2	1	85	0.796	EQ	44	0	8.5	29
8	96	T3	1	1	2	2	1	90	0.76	EQ	44	0	8.5	29
8	96	T9	1	1	1	2	1	80	0.796	EQ	44	0	8.5	29
8	95	T0.75	2	1	1	1	1	89	0.85	EQ	36	1	1	16
8	95	T18	1	1	1	1	1	100	1	EQ	36	1	1	16
8	95	T3	1	1	1	2	1	90	0.796	EQ	36	1	1	16
8	95	T9	1	1	1	1	1	99	1	EQ	36	1	1	16
8	94	T0.75	1	3	3	2	2	65	0.148	EQ	49	1	8.5	21
8	94	T3	1	1	2	2	1	94	0.76	EQ	49	1	8.5	21
8	94	T9	1	1	1	1	1	90	1	EQ	49	1	8.5	21
8	93	T0.75	1	1	2	1	2	90	0.812	EQ	63	1	8.5	15
8	93	T18	1	1	1	2	2	80	0.725	EQ	63	1	8.5	15
8	93	T3	1	1	1	1	2	50	0.848	EQ	63	1	8.5	15
8	93	T9	1	1	1	1	1	89	1	EQ	63	1	8.5	15
8	92	T0.75	2	3	3	2	1	65	0.15	EQ	46	0	11	27
8	92	T18	1	1	1	2	1	75	0.796	EQ	46	0	11	27
8	92	T3	1	1	2	2	1	70	0.76	EQ	46	0	11	27
8	92	T9	1	1	1	1	1	70	1	EQ	46	0	11	27
8	91	T0.75	1	2	2	1	1	85	0.779	EQ	52	1	5	13
8	91	T18	1	1	1	1	1	95	1	EQ	52	1	5	13
8	91	T3	1	1	1	1	1	99	1	EQ	52	1	5	13
8	91	T9	1	1	1	1	1	95	1	EQ	52	1	5	13
8	90	T0.75	1	2	1	2	1	77	0.692	EQ	20	1	26.5	24
8	89	T0.75	2	1	1	1	1	80	0.85	EQ	24	1	9	17
8	89	T18	1	1	1	2	1	90	0.796	EQ	24	1	9	17
8	89	T3	1	1	2	1	1	80	0.883	EQ	24	1	9	17
8	89	T9	1	1	1	1	1	90	1	EQ	24	1	9	17
8	88	T0.75	3	2	3	3	2	30	-0.319	EQ	31	1	21.5	25

8	88	T18	1	1	1	1	1	90	1	EQ	31	1	21.5	25
8	88	T3	2	1	2	2	1	60	0.691	EQ	31	1	21.5	25
8	88	T9	1	1	1	2	2	75	0.725	EQ	31	1	21.5	25
8	87	T0.75	1	2	1	2	1	60	0.692	EQ	34	1	3.5	25
8	87	T18	1	1	1	2	2	80	0.725	EQ	34	1	3.5	25
8	87	T3	1	1	2	2	2	70	0.689	EQ	34	1	3.5	25
8	87	T9	1	1	2	2	1	60	0.76	EQ	34	1	3.5	25
8	86	T0.75	1	3	3	3	2	#N/B	-0.115	EQ	44	0	12.25	38
8	85	T0.75	3	2	2	2	2	24	0.002	EQ	55	1	0.5	21
8	85	T18	2	1	2	2	1	39	0.691	EQ	55	1	0.5	21
8	85	T3	2	1	2	2	1	60	0.691	EQ	55	1	0.5	21
8	85	T9	2	1	1	1	1	60	0.85	EQ	55	1	0.5	21
8	84	T0.75	2	2	2	2	2	69	0.516	EQ	41	0	7.75	26
8	84	T18	1	1	1	1	2	70	0.848	EQ	41	0	7.75	26
8	84	T3	1	1	2	1	2	#N/B	0.812	EQ	41	0	7.75	26
8	84	T9	1	1	2	1	2	60	0.812	EQ	41	0	7.75	26
8	83	T0.75	3	1	1	2	1	65	0.213	EQ	19	1	8	26
8	83	T18	1	1	1	1	1	96	1	EQ	19	1	8	26
8	83	T3	1	1	2	1	1	85	0.883	EQ	19	1	8	26
8	83	T9	1	1	1	1	1	#N/B	1	EQ	19	1	8	26
8	82	T0.75	3	1	3	2	1	80	0.119	EQ	42	1	1.5	23
8	82	T18	1	1	1	1	1	100	1	EQ	42	1	1.5	23
8	82	T3	1	1	1	2	1	95	0.796	EQ	42	1	1.5	23
8	81	T0.75	1	1	2	1	2	70	0.812	EQ	36	1	5.25	28
8	81	T18	1	1	1	1	1	70	1	EQ	36	1	5.25	28
8	81	T3	1	1	2	1	1	68	0.883	EQ	36	1	5.25	28
8	81	T9	1	1	1	2	1	70	0.796	EQ	36	1	5.25	28
8	80	T0.75	2	2	1	2	2	60	0.552	EQ	51	1	2.5	27
8	80	T18	1	1	2	1	2	68	0.812	EQ	51	1	2.5	27
8	80	T3	1	1	2	1	1	96	0.883	EQ	51	1	2.5	27
8	80	T9	1	1	2	2	2	80	0.689	EQ	51	1	2.5	27
8	79	T0.75	2	2	2	2	2	30	0.516	EQ	20	1	9.5	23
8	79	T18	1	1	1	2	2	90	0.725	EQ	20	1	9.5	23
8	79	T3	1	1	1	2	2	70	0.725	EQ	20	1	9.5	23
8	79	T9	1	1	1	2	1	80	0.796	EQ	20	1	9.5	23
8	78	T0.75	1	1	1	1	1	65	1	EQ	22	1	15	30
8	78	T3	1	1	1	2	1	85	0.796	EQ	22	1	15	30
8	78	T9	1	1	1	1	1	90	1	EQ	22	1	15	30
8	77	T0.75	2	1	1	1	1	100	0.85	EQ	53	0	5	21
8	77	T18	2	1	1	1	1	90	0.85	EQ	53	0	5	21
8	77	T3	2	1	1	2	1	90	0.727	EQ	53	0	5	21
8	77	T9	2	1	1	2	1	100	0.727	EQ	53	0	5	21
8	76	T0.75	2	3	3	2	1	68	0.15	EQ	61	1	8	30
8	76	T18	1	1	3	1	2	60	0.485	EQ	61	1	8	30
8	76	T3	1	2	3	2	1	60	0.329	EQ	61	1	8	30
8	76	T9	1	2	2	2	1	68	0.656	EQ	61	1	8	30
8	75	T0.75	1	1	1	1	1	90	1	EQ	54	1	10	4
8	75	T3	1	1	1	2	1	94	0.796	EQ	54	1	10	4
8	75	T9	1	1	1	1	1	90	1	EQ	54	1	10	4
8	74	T0.75	2	3	3	2	2	85	0.079	EQ	19	0	32	33
8	74	T3	1	1	1	2	2	30	0.725	EQ	19	0	32	33

8	73	T0.75	2	2	2	2	1	80	0.587	EQ	26	1	8	9
8	73	T3	1	1	1	1	1	98	1	EQ	26	1	8	9
8	73	T9	1	1	1	1	1	92	1	EQ	26	1	8	9
8	72	T0.75	3	2	3	2	1	94	0.015	EQ	38	1	5	12
8	72	T3	1	1	1	1	1	88	1	EQ	38	1	5	12
8	72	T9	2	1	2	1	1	78	0.814	EQ	38	1	5	12
8	71	T0.75	1	1	1	2	1	85	0.796	EQ	44	1	15	8
8	71	T18	1	1	1	1	1	88	1	EQ	44	1	15	8
8	71	T3	1	1	1	1	1	95	1	EQ	44	1	15	8
8	71	T9	1	1	1	1	1	95	1	EQ	44	1	15	8
8	70	T0.75	1	1	1	1	1	75	1	EQ	48	1	14	11
8	70	T18	1	1	1	1	1	87	1	EQ	48	1	14	11
8	70	T3	1	1	1	1	1	90	1	EQ	48	1	14	11
8	70	T9	1	1	1	1	1	100	1	EQ	48	1	14	11
8	69	T0.75	1	1	2	1	1	78	0.883	EQ	41	1	9	8
8	69	T18	1	1	1	1	1	85	1	EQ	41	1	9	8
8	69	T3	1	1	2	1	1	78	0.883	EQ	41	1	9	8
8	69	T9	1	1	1	1	1	84	1	EQ	41	1	9	8
8	68	T0.75	1	1	1	1	1	88	1	EQ	27	1	6	13
8	67	T0.75	1	2	2	1	2	65	0.708	EQ	22	0	2.5	11
8	66	T0.75	2	2	1	2	1	#N/B	0.623	EQ	38	1	10	20
8	66	T3	1	1	2	2	1	75	0.76	EQ	38	1	10	20
8	66	T9	1	1	1	2	1	80	0.796	EQ	38	1	10	20
8	65	T0.75	2	2	2	2	2	80	0.516	EQ	31	1	17	31
8	65	T18	1	1	1	1	1	90	1	EQ	31	1	17	31
8	65	T3	1	1	1	2	1	87	0.796	EQ	31	1	17	31
8	65	T9	1	1	2	1	1	88	0.883	EQ	31	1	17	31
8	64	T0.75	1	1	2	2	2	60	0.689	EQ	51	0	10	9
8	64	T18	1	1	1	1	1	80	1	EQ	51	0	10	9
8	64	T3	1	1	2	1	2	60	0.812	EQ	51	0	10	9
8	64	T9	1	1	1	1	1	70	1	EQ	51	0	10	9
8	63	T0.75	1	1	1	1	1	85	1	EQ	23	1	11.5	7
8	63	T18	1	1	1	1	1	75	1	EQ	23	1	11.5	7
8	63	T3	1	1	1	1	1	90	1	EQ	23	1	11.5	7
8	63	T9	1	1	1	1	1	100	1	EQ	23	1	11.5	7
8	62	T0.75	1	1	1	1	1	80	1	EQ	46	1	1	4
8	62	T18	1	1	1	1	1	95	1	EQ	46	1	1	4
8	62	T3	1	1	1	1	1	85	1	EQ	46	1	1	4
8	62	T9	1	1	1	1	1	90	1	EQ	46	1	1	4
8	61	T0.75	2	1	1	1	1	#N/B	0.85	EQ	64	1	5	4
8	61	T9	1	1	1	1	1	76	1	EQ	64	1	5	4
8	60	T0.75	1	1	1	1	2	70	0.848	EQ	31	1	6	5
8	60	T18	1	1	1	1	1	80	1	EQ	31	1	6	5
8	60	T3	1	1	1	1	1	90	1	EQ	31	1	6	5
8	60	T9	1	1	1	1	2	70	0.848	EQ	31	1	6	5
8	59	T0.75	1	2	2	2	1	70	0.656	EQ	21	1	13	11
8	59	T18	1	1	1	1	1	75	1	EQ	21	1	13	11
8	59	T9	1	1	1	1	1	70	1	EQ	21	1	13	11
8	58	T3	1	1	1	2	1	94	0.796	EQ	23	1	1	3
8	58	T9	1	1	1	2	1	95	0.796	EQ	23	1	1	3
8	57	T0.75	1	2	2	2	2	70	0.585	EQ	33	1	6	4

8	57	T18	1	1	1	2	2	65	0.725	EQ	33	1	6	4
8	57	T3	1	1	2	2	2	70	0.689	EQ	33	1	6	4
8	56	T0.75	1	1	2	2	1	80	0.76	EQ	21	1	5	4
8	56	T3	1	1	1	1	1	92	1	EQ	21	1	5	4
8	56	T9	1	1	1	1	1	98	1	EQ	21	1	5	4
8	55	T0.75	1	1	1	2	1	85	0.796	EQ	31	1	3	8
8	55	T3	1	1	1	2	1	82	0.796	EQ	31	1	3	8
8	54	T0.75	1	1	1	1	1	80	1	EQ	35	1	9	15
8	54	T3	1	1	1	1	1	78	1	EQ	35	1	9	15
8	53	T0.75	1	1	1	1	1	100	1	EQ	62	1	5	3
8	53	T3	1	1	1	1	1	100	1	EQ	62	1	5	3
8	52	T0.75	1	1	1	1	1	90	1	EQ	30	1	5	11
8	51	T0.75	1	1	1	1	1	98	1	EQ	35	1	6	10
8	51	T18	1	1	1	1	1	#N/B	1	EQ	35	1	6	10
8	51	T3	1	1	1	1	1	100	1	EQ	35	1	6	10
8	50	T0.75	1	1	1	1	1	90	1	EQ	46	1	5	5
8	50	T3	2	1	1	1	1	70	0.85	EQ	46	1	5	5
8	49	T0.75	1	1	1	1	1	70	1	EQ	54	1	13	10
8	49	T18	1	1	1	1	1	94	1	EQ	54	1	13	10
8	49	T3	1	1	1	1	1	80	1	EQ	54	1	13	10
8	49	T9	1	1	1	1	1	100	1	EQ	54	1	13	10
8	48	T0.75	1	1	2	2	2	65	0.689	EQ	50	0	4	14
8	48	T3	1	1	1	2	1	90	0.796	EQ	50	0	4	14
8	48	T9	1	1	1	2	1	80	0.796	EQ	50	0	4	14
8	47	T0.75	1	1	2	2	1	50	0.76	EQ	39	1	10	16
8	47	T18	1	1	1	1	1	80	1	EQ	39	1	10	16
8	47	T3	1	1	1	1	2	75	0.848	EQ	39	1	10	16
8	47	T9	1	1	1	1	1	80	1	EQ	39	1	10	16
8	46	T0.75	2	1	1	1	1	60	0.85	EQ	30	1	5	11
8	45	T0.75	1	1	1	1	1	99	1	EQ	42	1	10	6
8	45	T18	1	1	1	1	1	98	1	EQ	42	1	10	6
8	45	T3	1	1	1	1	1	100	1	EQ	42	1	10	6
8	45	T9	1	1	1	1	1	100	1	EQ	42	1	10	6
8	44	T0.75	1	1	1	1	1	75	1	EQ	40	1	2	5
8	44	T3	1	1	1	1	1	90	1	EQ	40	1	2	5
8	43	T18	1	1	1	1	1	70	1	EQ	53	0	3	6
8	42	T0.75	1	1	2	1	1	80	0.883	EQ	30	0	10	19
8	42	T18	1	1	1	1	1	90	1	EQ	30	0	10	19
8	42	T3	1	1	2	1	1	80	0.883	EQ	30	0	10	19
8	42	T9	1	1	1	1	1	90	1	EQ	30	0	10	19
8	41	T0.75	2	2	2	3	3	20	-0.181	EQ	38	0	8	31
8	41	T3	1	1	2	2	2	50	0.689	EQ	38	0	8	31
8	41	T9	1	1	1	2	2	50	0.725	EQ	38	0	8	31
8	40	T0.75	1	1	2	2	1	80	0.76	EQ	37	1	14	10
8	40	T18	1	1	1	1	1	97	1	EQ	37	1	14	10
8	40	T3	1	1	1	1	1	92	1	EQ	37	1	14	10
8	40	T9	1	1	1	1	2	90	0.848	EQ	37	1	14	10
8	39	T0.75	1	1	1	1	1	75	1	EQ	26	1	7	7
8	39	T18	1	1	1	1	1	90	1	EQ	26	1	7	7
8	39	T3	1	1	1	1	1	85	1	EQ	26	1	7	7
8	39	T9	1	1	1	1	1	90	1	EQ	26	1	7	7

8	38	T0.75	1	1	1	2	1	73	0.796	EQ	49	1	18	8
8	38	T18	1	1	1	1	1	88	1	EQ	49	1	18	8
8	38	T3	1	1	1	1	1	78	1	EQ	49	1	18	8
8	38	T9	1	1	1	1	1	85	1	EQ	49	1	18	8
8	37	T0.75	1	1	1	2	1	53	0.796	EQ	49	0	8	7
8	37	T18	1	1	1	2	1	50	0.796	EQ	49	0	8	7
8	37	T3	1	1	2	2	2	55	0.689	EQ	49	0	8	7
8	37	T9	1	1	1	2	2	58	0.725	EQ	49	0	8	7
8	36	T0.75	1	2	3	2	1	30	0.329	EQ	33	0	9	6
8	36	T18	1	1	1	2	1	97	0.796	EQ	33	0	9	6
8	36	T3	1	1	1	1	1	90	1	EQ	33	0	9	6
8	36	T9	1	1	1	2	1	85	0.796	EQ	33	0	9	6
8	35	T0.75	1	3	2	1	1	#N/B	0.4	EQ	72	0	6	6
8	35	T18	1	1	1	1	1	#N/B	1	EQ	72	0	6	6
8	35	T3	1	1	1	1	2	85	0.848	EQ	72	0	6	6
8	35	T9	1	1	1	1	1	#N/B	1	EQ	72	0	6	6
8	34	T18	1	1	2	2	1	70	0.76	EQ	31	0	18	13
8	34	T9	1	2	2	2	1	85	0.656	EQ	31	0	18	13
8	33	T0.75	1	2	2	2	1	65	0.656	EQ	41	1	4	10
8	33	T18	1	1	1	1	1	83	1	EQ	41	1	4	10
8	33	T3	1	1	2	2	1	78	0.76	EQ	41	1	4	10
8	33	T9	1	1	2	1	1	80	0.883	EQ	41	1	4	10
8	32	T0.75	3	3	2	2	2	#N/B	-0.108	EQ	34	1	23.5	16
8	31	T0.75	2	1	2	1	1	85	0.814	EQ	22	1	13	9
8	31	T18	1	1	1	1	1	97	1	EQ	22	1	13	9
8	31	T3	1	1	1	1	1	97	1	EQ	22	1	13	9
8	31	T9	1	1	1	1	1	95	1	EQ	22	1	13	9
8	30	T0.75	1	1	1	2	2	70	0.725	EQ	30	1	3	7
8	30	T18	1	1	1	1	1	70	1	EQ	30	1	3	7
8	30	T3	1	1	1	1	1	#N/B	1	EQ	30	1	3	7
8	29	T0.75	1	1	2	1	1	85	0.883	EQ	49	1	4.5	7
8	29	T18	1	1	1	1	1	80	1	EQ	49	1	4.5	7
8	29	T3	1	1	2	1	1	95	0.883	EQ	49	1	4.5	7
8	29	T9	1	1	1	1	1	90	1	EQ	49	1	4.5	7
8	28	T0.75	1	2	3	3	1	65	0.066	EQ	36	1	7	10
8	28	T18	1	1	1	2	1	75	0.796	EQ	36	1	7	10
8	28	T3	2	1	2	2	2	65	0.62	EQ	36	1	7	10
8	28	T9	2	1	1	2	1	70	0.727	EQ	36	1	7	10
8	27	T0.75	1	2	2	1	1	70	0.779	EQ	37	0	8	6
8	27	T18	1	1	2	1	1	70	0.883	EQ	37	0	8	6
8	27	T3	1	1	1	1	1	80	1	EQ	37	0	8	6
8	27	T9	1	1	1	1	1	50	1	EQ	37	0	8	6
8	26	T0.75	1	2	1	1	1	71	0.815	EQ	58	1	7	14
8	26	T18	1	1	1	2	2	60	0.725	EQ	58	1	7	14
8	26	T3	1	2	3	2	2	50	0.258	EQ	58	1	7	14
8	26	T9	1	1	1	1	2	80	0.848	EQ	58	1	7	14
8	25	T0.75	1	1	1	1	2	95	0.848	EQ	30	0	4	12
8	25	T18	1	1	1	2	2	97	0.725	EQ	30	0	4	12
8	25	T3	1	1	1	2	1	100	0.796	EQ	30	0	4	12
8	25	T9	1	1	1	2	1	100	0.796	EQ	30	0	4	12
8	24	T0.75	1	1	1	1	1	90	1	EQ	37	1	10.5	12

8	24	T18	1	1	1	1	1	100	1	EQ	37	1	10.5	12
8	24	T3	1	1	1	1	1	100	1	EQ	37	1	10.5	12
8	24	T9	1	1	1	1	1	93	1	EQ	37	1	10.5	12
8	23	T0.75	1	1	1	1	1	90	1	EQ	26	0	4	17
8	23	T3	1	1	1	1	1	97	1	EQ	26	0	4	17
8	23	T9	1	1	1	1	1	98	1	EQ	26	0	4	17
8	22	T0.75	1	2	1	2	2	65	0.621	EQ	40	1	11	12
8	22	T18	1	1	1	2	2	79	0.725	EQ	40	1	11	12
8	22	T3	1	1	1	2	2	70	0.725	EQ	40	1	11	12
8	22	T9	1	1	2	2	2	65	0.689	EQ	40	1	11	12
8	21	T0.75	1	3	1	1	1	80	0.436	EQ	54	1	4	14
8	21	T18	1	1	1	1	1	90	1	EQ	54	1	4	14
8	21	T3	1	1	1	2	1	#N/B	0.796	EQ	54	1	4	14
8	21	T9	1	1	1	1	2	85	0.848	EQ	54	1	4	14
8	20	T0.75	1	1	1	2	1	94	0.796	EQ	58	1	4	10
8	20	T18	1	1	1	1	1	98	1	EQ	58	1	4	10
8	20	T3	1	1	3	2	3	75	0.197	EQ	58	1	4	10
8	20	T9	1	1	1	1	1	77	1	EQ	58	1	4	10
8	19	T0.75	2	1	1	1	1	90	0.85	EQ	19	0	6.5	14
8	19	T3	1	1	1	1	1	70	1	EQ	19	0	6.5	14
8	18	T0.75	1	1	1	2	2	65	0.725	EQ	44	1	10	18
8	18	T18	1	1	1	1	1	88	1	EQ	44	1	10	18
8	18	T3	1	1	1	1	1	74	1	EQ	44	1	10	18
8	18	T9	1	1	1	1	1	86	1	EQ	44	1	10	18
8	17	T0.75	2	2	2	2	2	80	0.516	EQ	47	0	4	15
8	17	T3	1	1	1	2	1	90	0.796	EQ	47	0	4	15
8	17	T9	1	1	1	1	1	80	1	EQ	47	0	4	15
8	16	T0.75	1	1	1	2	1	76	0.796	EQ	39	1	7	6
8	16	T3	1	1	1	1	1	#N/B	1	EQ	39	1	7	6
8	16	T9	1	1	1	1	1	90	1	EQ	39	1	7	6
8	15	T0.75	2	2	2	2	1	51	0.587	EQ	37	0	7	6
8	15	T18	1	1	1	2	1	93	0.796	EQ	37	0	7	6
8	15	T3	1	1	1	2	1	90	0.796	EQ	37	0	7	6
8	15	T9	1	1	1	2	1	80	0.796	EQ	37	0	7	6
8	14	T0.75	1	1	1	1	1	85	1	EQ	47	1	4	7
8	14	T18	1	1	1	1	1	90	1	EQ	47	1	4	7
8	14	T3	1	1	1	1	1	95	1	EQ	47	1	4	7
8	14	T9	1	1	1	1	1	94	1	EQ	47	1	4	7
8	13	T0.75	1	1	2	1	1	78	0.883	EQ	41	1	4	4
8	13	T18	1	1	1	1	1	90	1	EQ	41	1	4	4
8	13	T3	1	1	1	1	1	90	1	EQ	41	1	4	4
8	13	T9	1	1	1	1	1	90	1	EQ	41	1	4	4
8	12	T0.75	2	2	3	2	2	60	0.189	EQ	20	1	6	21
8	12	T18	1	1	1	1	1	#N/B	1	EQ	20	1	6	21
8	12	T3	1	1	1	1	1	70	1	EQ	20	1	6	21
8	12	T9	1	1	1	1	1	77	1	EQ	20	1	6	21
8	11	T0.75	1	1	1	1	1	90	1	EQ	41	1	3	10
8	11	T18	1	1	1	1	2	100	0.848	EQ	41	1	3	10
8	11	T3	1	1	1	1	1	70	1	EQ	41	1	3	10
8	11	T9	1	1	1	2	1	100	0.796	EQ	41	1	3	10
8	10	T0.75	1	1	2	2	2	70	0.689	EQ	48	1	5	15

8	10	T18	1	1	1	1	1	90	1	EQ	48	1	5	15
8	10	T3	1	1	1	1	1	90	1	EQ	48	1	5	15
8	10	T9	1	1	1	1	1	98	1	EQ	48	1	5	15
8	9	T0.75	1	1	1	1	2	80	0.848	EQ	27	0	8	17
8	9	T18	1	1	1	1	1	95	1	EQ	27	0	8	17
8	9	T3	1	1	1	1	2	80	0.848	EQ	27	0	8	17
8	9	T9	1	1	1	1	1	87	1	EQ	27	0	8	17
8	8	T0.75	1	2	2	2	2	73	0.585	EQ	39	1	2.5	6
8	8	T18	1	1	1	2	1	90	0.796	EQ	39	1	2.5	6
8	8	T3	1	1	2	2	2	75	0.689	EQ	39	1	2.5	6
8	8	T9	1	1	1	2	2	87	0.725	EQ	39	1	2.5	6
8	7	T0.75	1	1	1	1	1	95	1	EQ	42	1	1.25	15
8	7	T18	1	1	1	1	1	95	1	EQ	42	1	1.25	15
8	7	T3	1	1	1	2	1	95	0.796	EQ	42	1	1.25	15
8	7	T9	1	1	1	1	1	84	1	EQ	42	1	1.25	15
8	6	T0.75	1	1	1	1	1	90	1	EQ	19	1	9	11
8	6	T3	1	1	1	1	1	100	1	EQ	19	1	9	11
8	5	T0.75	1	1	2	1	1	92	0.883	EQ	40	1	1.5	6
8	5	T18	1	1	1	2	1	80	0.796	EQ	40	1	1.5	6
8	5	T3	1	1	1	3	1	70	0.264	EQ	40	1	1.5	6
8	5	T9	1	1	1	2	1	88	0.796	EQ	40	1	1.5	6
8	4	T0.75	1	1	3	2	1	70	0.433	EQ	34	1	2.25	6
8	4	T18	1	1	1	1	1	95	1	EQ	34	1	2.25	6
8	4	T3	1	1	2	1	1	82	0.883	EQ	34	1	2.25	6
8	4	T9	1	1	1	2	1	90	0.796	EQ	34	1	2.25	6
8	3	T0.75	2	1	2	2	2	70	0.62	EQ	51	1	8	5
8	3	T18	1	1	1	1	2	#N/B	0.848	EQ	51	1	8	5
8	3	T3	2	1	2	2	1	70	0.691	EQ	51	1	8	5
8	3	T9	1	1	1	1	1	60	1	EQ	51	1	8	5
8	2	T0.75	1	2	2	1	1	70	0.779	EQ	47	1	7	14
8	1	T0.75	2	2	2	2	2	35	0.516	EQ	29	0	5	20
8	1	T18	1	1	1	1	1	75	1	EQ	29	0	5	20
8	1	T3	1	1	1	2	2	83	0.725	EQ	29	0	5	20
8	1	T9	1	1	1	1	1	84	1	EQ	29	0	5	20
9	307	T18	1	1	1	1	3	#N/B	0.414	SF	27	0	40	40
9	307	T3	1	1	2	2	3	#N/B	0.255	SF	27	0	40	40
9	306	T18	2	1	2	2	2	#N/B	0.62	SF	34	1	17	11
9	306	T3	2	2	1	3	2	#N/B	0.02	SF	34	1	17	11
9	305	T18	2	1	2	1	2	#N/B	0.743	SF	32	0	4	7
9	305	T3	1	1	2	2	1	#N/B	0.76	SF	32	0	4	7
9	304	T3	2	2	3	2	2	#N/B	0.189	SF	33	1	29	63
9	303	T3	2	1	2	2	2	#N/B	0.62	SF	36	1	5	15
9	302	T18	2	1	2	3	2	#N/B	0.088	SF	48	1	3	7
9	302	T3	2	1	2	3	3	#N/B	-0.077	SF	48	1	3	7
9	301	T18	2	1	2	2	2	#N/B	0.62	SF	58	1	23	20
9	301	T3	2	1	2	3	2	#N/B	0.088	SF	58	1	23	20
9	300	T18	2	1	2	3	2	#N/B	0.088	SF	76	1	7	11
9	300	T3	2	1	2	2	1	#N/B	0.691	SF	76	1	7	11
9	299	T18	1	1	2	2	2	#N/B	0.689	SF	44	1	6	7
9	299	T3	2	1	1	2	2	#N/B	0.656	SF	44	1	6	7
9	298	T18	2	1	1	2	2	#N/B	0.656	SF	63	1	16	17

9	298	T3	1	2	2	2	2	#N/B	0.585	SF	63	1	16	17
9	297	T18	2	1	2	1	2	#N/B	0.743	SF	44	0	35.5	64
9	296	T18	2	1	3	2	1	#N/B	0.364	SF	41	0	4	15
9	296	T3	2	3	3	2	2	#N/B	0.079	SF	41	0	4	15
9	295	T18	1	1	2	2	1	#N/B	0.76	SF	62	1	2	27
9	294	T18	1	1	1	2	2	#N/B	0.725	SF	31	1	10	12
9	294	T3	2	1	2	3	2	#N/B	0.088	SF	31	1	10	12
9	293	T18	1	1	1	1	1	#N/B	1	SF	40	1	8	24
9	293	T3	1	2	2	2	3	#N/B	0.151	SF	40	1	8	24
9	292	T18	1	1	1	2	1	#N/B	0.796	SF	40	1	7	21
9	292	T3	1	1	2	2	1	#N/B	0.76	SF	40	1	7	21
9	291	T18	2	1	2	2	2	#N/B	0.62	SF	36	1	25	48
9	291	T3	1	1	1	2	2	#N/B	0.725	SF	36	1	25	48
9	290	T18	2	1	2	2	2	#N/B	0.62	SF	64	0	4	17
9	290	T3	1	1	2	2	2	#N/B	0.689	SF	64	0	4	17
9	289	T3	1	1	1	1	1	#N/B	1	SF	48	1	20	13
9	288	T18	1	1	1	1	1	#N/B	1	SF	29	1	17	51
9	288	T3	1	1	2	2	2	#N/B	0.689	SF	29	1	17	51
9	287	T18	1	1	1	1	1	#N/B	1	SF	38	1	44	76
9	287	T3	1	1	1	2	2	#N/B	0.725	SF	38	1	44	76
9	286	T18	2	1	1	1	2	#N/B	0.779	SF	43	1	31	72
9	286	T3	2	1	2	3	2	#N/B	0.088	SF	43	1	31	72
9	285	T18	1	1	2	2	1	#N/B	0.76	SF	50	1	22	32
9	285	T3	2	1	2	2	2	#N/B	0.62	SF	50	1	22	32
9	284	T18	2	1	1	2	2	#N/B	0.656	SF	58	1	17	40
9	284	T3	1	1	1	1	2	#N/B	0.848	SF	58	1	17	40
9	283	T18	2	1	1	2	2	#N/B	0.656	SF	54	0	23	35
9	283	T3	2	2	2	3	2	#N/B	-0.016	SF	54	0	23	35
9	282	T18	2	1	1	2	2	#N/B	0.656	SF	53	1	31	33
9	282	T3	1	1	2	2	2	#N/B	0.689	SF	53	1	31	33
9	281	T18	1	1	1	2	2	#N/B	0.725	SF	27	1	12	23
9	281	T3	2	1	2	2	2	#N/B	0.62	SF	27	1	12	23
9	280	T18	1	1	1	2	1	#N/B	0.796	SF	52	1	6	9
9	280	T3	1	1	2	2	3	#N/B	0.255	SF	52	1	6	9
9	279	T18	1	1	1	2	1	#N/B	0.796	SF	58	1	11	9
9	278	T18	1	1	1	1	1	#N/B	1	SF	53	1	8	17
9	278	T3	2	1	2	2	1	#N/B	0.691	SF	53	1	8	17
9	277	T18	1	1	1	2	2	#N/B	0.725	SF	27	1	44	70
9	277	T3	1	1	1	2	1	#N/B	0.796	SF	27	1	44	70
9	276	T18	1	1	1	2	2	#N/B	0.725	SF	43	1	7	33
9	276	T3	1	2	1	1	2	#N/B	0.744	SF	43	1	7	33
9	275	T18	1	1	2	2	2	#N/B	0.689	SF	47	1	9.5	42
9	274	T18	1	1	1	2	1	#N/B	0.796	SF	31	1	4.5	8
9	274	T3	1	1	2	2	1	#N/B	0.76	SF	31	1	4.5	8
9	273	T18	1	1	1	2	2	#N/B	0.725	SF	46	1	19.25	43
9	273	T3	2	1	2	2	2	#N/B	0.62	SF	46	1	19.25	43
9	272	T18	1	1	1	1	2	#N/B	0.848	SF	55	1	18	23
9	272	T3	2	1	2	1	1	#N/B	0.814	SF	55	1	18	23
9	271	T18	1	1	1	1	1	#N/B	1	SF	22	1	29	38
9	270	T18	1	1	1	1	1	#N/B	1	SF	62	1	35	36
9	270	T3	1	1	1	2	1	#N/B	0.796	SF	62	1	35	36

9	269	T18	1	1	1	1	2	#N/B	0.848	SF	64	1	19	26
9	269	T3	1	1	2	2	3	#N/B	0.255	SF	64	1	19	26
9	268	T18	1	1	1	1	1	#N/B	1	SF	29	1	6	21
9	268	T3	1	1	1	1	1	#N/B	1	SF	29	1	6	21
9	267	T3	1	1	2	2	2	#N/B	0.689	SF	51	1	1	21
9	266	T3	2	1	2	2	3	#N/B	0.186	SF	45	0	8	12
9	265	T3	1	1	1	1	2	#N/B	0.848	SF	58	1	10	4
9	264	T3	1	1	2	2	2	#N/B	0.689	SF	36	0	19.5	22
9	263	T3	1	1	2	2	2	#N/B	0.689	SF	43	1	50	65
9	262	T3	2	1	3	2	3	#N/B	0.128	SF	44	1	65	66
9	261	T3	2	1	2	2	2	#N/B	0.62	SF	21	0	6	10
9	260	T3	1	1	2	2	2	#N/B	0.689	SF	38	1	9	17
9	259	T3	1	1	2	2	2	#N/B	0.689	SF	28	0	34	32
9	258	T3	1	1	1	2	2	#N/B	0.725	SF	40	1	9	19
9	257	T3	1	2	3	1	2	#N/B	0.381	SF	36	0	7	20
9	256	T3	1	1	2	2	3	#N/B	0.255	SF	38	1	5	6
9	255	T3	2	1	2	1	1	#N/B	0.814	SF	43	1	19	52
9	254	T18	1	1	2	2	2	#N/B	0.689	SF	43	1	1	4
9	254	T3	1	1	1	1	2	#N/B	0.848	SF	43	1	1	4
9	253	T18	2	1	2	3	1	#N/B	0.159	SF	48	1	11	9
9	253	T3	2	1	1	1	1	#N/B	0.85	SF	48	1	11	9
9	252	T18	2	1	2	2	3	#N/B	0.186	SF	43	1	13	20
9	252	T3	1	1	3	2	2	#N/B	0.362	SF	43	1	13	20
9	251	T18	1	1	2	1	2	#N/B	0.812	SF	45	0	2	21
9	251	T3	2	1	2	2	2	#N/B	0.62	SF	45	0	2	21
9	250	T18	1	1	1	2	2	#N/B	0.725	SF	72	1	10	26
9	250	T3	1	1	3	2	2	#N/B	0.362	SF	72	1	10	26
9	249	T18	1	1	1	1	2	#N/B	0.848	SF	21	1	3	19
9	249	T3	1	1	1	1	1	#N/B	1	SF	21	1	3	19
9	248	T18	1	1	1	2	2	#N/B	0.725	SF	46	1	29	33
9	248	T3	2	1	2	2	1	#N/B	0.691	SF	46	1	29	33
9	247	T18	1	1	2	1	2	#N/B	0.812	SF	55	0	1.5	5
9	247	T3	1	1	1	1	1	#N/B	1	SF	55	0	1.5	5
9	246	T18	1	1	1	2	3	#N/B	0.291	SF	46	1	5.5	26
9	246	T3	1	1	2	2	2	#N/B	0.689	SF	46	1	5.5	26
9	245	T18	1	1	1	2	2	#N/B	0.725	SF	62	1	3.5	9
9	245	T3	1	1	1	2	2	#N/B	0.725	SF	62	1	3.5	9
9	243	T18	1	1	1	2	2	#N/B	0.725	SF	70	1	5	17
9	243	T3	1	1	2	2	1	#N/B	0.76	SF	70	1	5	17
9	242	T18	1	1	1	1	1	#N/B	1	SF	44	1	12	17
9	242	T3	1	1	1	2	1	#N/B	0.796	SF	44	1	12	17
9	241	T18	1	1	1	1	2	#N/B	0.848	SF	30	1	18	16
9	241	T3	1	1	1	2	2	#N/B	0.725	SF	30	1	18	16
9	240	T18	1	1	1	2	2	#N/B	0.725	SF	62	1	17	13
9	240	T3	1	1	1	2	1	#N/B	0.796	SF	62	1	17	13
9	239	T18	1	1	2	2	1	#N/B	0.76	SF	53	0	25	26
9	239	T3	1	1	2	2	2	#N/B	0.689	SF	53	0	25	26
9	238	T18	1	1	2	1	1	#N/B	0.883	SF	44	1	17	13
9	238	T3	1	1	1	2	1	#N/B	0.796	SF	44	1	17	13
9	237	T18	1	1	1	2	1	#N/B	0.796	SF	58	1	16	15
9	237	T3	1	1	1	2	1	#N/B	0.796	SF	58	1	16	15

9	236	T18	1	1	1	1	1	#N/B	1	SF	67	1	23	9
9	236	T3	1	1	1	1	2	#N/B	0.848	SF	67	1	23	9
9	235	T18	1	1	1	1	1	#N/B	1	SF	19	0	5	4
9	235	T3	1	1	1	1	1	#N/B	1	SF	19	0	5	4
9	234	T18	2	1	1	2	1	#N/B	0.727	SF	29	0	5	9
9	234	T3	1	1	1	2	1	#N/B	0.796	SF	29	0	5	9
9	233	T18	2	2	2	2	2	#N/B	0.516	SF	42	1	60	93
9	233	T3	1	1	2	2	1	#N/B	0.76	SF	42	1	60	93
9	232	T18	1	1	1	1	1	#N/B	1	SF	26	1	2	5
9	232	T3	1	1	1	1	1	#N/B	1	SF	26	1	2	5
9	231	T18	1	1	1	2	2	#N/B	0.725	SF	45	1	2	2
9	231	T3	1	1	1	2	1	#N/B	0.796	SF	45	1	2	2
9	230	T18	1	1	1	1	1	#N/B	1	SF	46	1	25	52
9	229	T18	1	1	1	2	2	#N/B	0.725	SF	42	1	4	14
9	229	T3	2	1	2	2	2	#N/B	0.62	SF	42	1	4	14
9	228	T18	1	1	1	1	1	#N/B	1	SF	43	1	7	11
9	228	T3	1	1	1	1	1	#N/B	1	SF	43	1	7	11
9	227	T18	1	1	1	2	1	#N/B	0.796	SF	64	1	6	47
9	227	T3	1	1	1	1	1	#N/B	1	SF	64	1	6	47
9	226	T18	1	1	2	2	2	#N/B	0.689	SF	54	1	19	77
9	226	T3	1	1	1	2	1	#N/B	0.796	SF	54	1	19	77
9	225	T18	1	1	1	1	1	#N/B	1	SF	45	1	3.5	7
9	225	T3	1	1	1	1	1	#N/B	1	SF	45	1	3.5	7
9	224	T18	1	1	1	1	1	#N/B	1	SF	39	1	14	25
9	224	T3	2	1	1	2	1	#N/B	0.727	SF	39	1	14	25
9	223	T18	1	1	1	1	1	#N/B	1	SF	41	1	23	25
9	223	T3	1	1	1	2	2	#N/B	0.725	SF	41	1	23	25
9	222	T18	1	1	2	2	1	#N/B	0.76	SF	59	0	14	40
9	222	T3	2	1	2	2	1	#N/B	0.691	SF	59	0	14	40
9	221	T18	1	1	1	1	2	#N/B	0.848	SF	30	1	6	10
9	221	T3	1	1	1	1	1	#N/B	1	SF	30	1	6	10
9	220	T18	1	1	1	1	1	#N/B	1	SF	32	1	60	30
9	220	T3	2	1	1	2	1	#N/B	0.727	SF	32	1	60	30
9	219	T18	1	1	1	2	1	#N/B	0.796	SF	20	1	68	150
9	219	T3	2	1	2	1	2	#N/B	0.743	SF	20	1	68	150
9	218	T18	1	1	1	1	1	#N/B	1	SF	56	1	7	19
9	218	T3	1	1	1	1	2	#N/B	0.848	SF	56	1	7	19
9	217	T18	1	1	1	1	1	#N/B	1	SF	60	1	2	10
9	217	T3	2	1	1	2	2	#N/B	0.656	SF	60	1	2	10
9	216	T18	1	1	1	2	1	#N/B	0.796	SF	46	1	2	11
9	216	T3	1	1	1	1	1	#N/B	1	SF	46	1	2	11
9	214	T18	1	1	2	2	1	#N/B	0.76	SF	54	1	9.5	8
9	214	T3	1	1	1	1	1	#N/B	1	SF	54	1	9.5	8
9	213	T18	1	1	2	1	2	#N/B	0.812	SF	24	1	37	71
9	212	T18	1	1	1	1	1	#N/B	1	SF	63	1	11	23
9	212	T3	2	1	1	2	1	#N/B	0.727	SF	63	1	11	23
9	211	T18	1	1	1	1	1	#N/B	1	SF	52	0	3	5
9	211	T3	1	1	1	1	1	#N/B	1	SF	52	0	3	5
9	210	T18	1	1	1	2	1	#N/B	0.796	SF	45	1	6	24
9	210	T3	2	1	2	2	2	#N/B	0.62	SF	45	1	6	24
9	209	T18	1	1	1	1	1	#N/B	1	SF	59	1	6	21

9	209	T3	1	1	1	1	1	#N/B	1	SF	59	1	6	21
9	208	T18	2	1	2	2	1	#N/B	0.691	SF	30	0	22	74
9	208	T3	2	1	1	2	1	#N/B	0.727	SF	30	0	22	74
9	207	T18	1	1	1	2	1	#N/B	0.796	SF	47	1	37	37
9	207	T3	1	1	1	2	1	#N/B	0.796	SF	47	1	37	37
9	206	T18	1	1	1	2	1	#N/B	0.796	SF	18	1	6.5	13
9	206	T3	1	1	1	1	1	#N/B	1	SF	18	1	6.5	13
9	205	T18	1	1	1	1	2	#N/B	0.848	SF	33	1	9	14
9	205	T3	1	1	1	2	1	#N/B	0.796	SF	33	1	9	14
9	204	T18	1	1	1	1	1	#N/B	1	SF	41	1	7.25	13
9	204	T3	1	1	1	1	2	#N/B	0.848	SF	41	1	7.25	13
9	203	T18	1	1	1	2	1	#N/B	0.796	SF	39	1	57	154
9	203	T3	1	1	2	2	2	#N/B	0.689	SF	39	1	57	154
9	202	T18	1	1	1	1	1	#N/B	1	SF	50	1	5	10
9	202	T3	1	1	1	1	1	#N/B	1	SF	50	1	5	10
9	201	T18	1	1	1	1	1	#N/B	1	SF	48	1	13.5	25
9	201	T3	1	1	1	1	1	#N/B	1	SF	48	1	13.5	25
9	200	T18	1	1	1	1	1	#N/B	1	SF	42	1	25.5	34
9	200	T3	1	1	1	2	1	#N/B	0.796	SF	42	1	25.5	34
9	199	T18	1	1	1	1	2	#N/B	0.848	SF	42	0	1	12
9	199	T3	1	1	1	1	2	#N/B	0.848	SF	42	0	1	12
9	198	T18	1	1	1	1	1	#N/B	1	SF	33	1	2.5	10
9	198	T3	1	1	1	2	1	#N/B	0.796	SF	33	1	2.5	10
9	197	T18	1	1	1	1	1	#N/B	1	SF	34	1	8	9
9	197	T3	2	1	1	2	1	#N/B	0.727	SF	34	1	8	9
9	196	T18	1	1	1	1	1	#N/B	1	SF	61	1	6	9
9	196	T3	2	1	1	2	1	#N/B	0.727	SF	61	1	6	9
9	195	T18	1	1	2	2	1	#N/B	0.76	SF	48	0	1	17
9	195	T3	1	1	1	1	1	#N/B	1	SF	48	0	1	17
9	194	T18	1	1	1	2	1	#N/B	0.796	SF	31	0	14	37
9	194	T3	2	1	2	2	2	#N/B	0.62	SF	31	0	14	37
9	193	T18	1	1	1	2	1	#N/B	0.796	SF	48	1	3.5	13
9	193	T3	1	1	1	2	2	#N/B	0.725	SF	48	1	3.5	13
9	192	T18	1	1	1	1	1	#N/B	1	SF	36	1	2.75	22
9	192	T3	1	1	1	1	1	#N/B	1	SF	36	1	2.75	22
9	191	T18	1	1	1	1	1	#N/B	1	SF	37	1	8	17
9	191	T3	1	1	1	1	1	#N/B	1	SF	37	1	8	17
9	190	T18	1	1	1	1	1	#N/B	1	SF	56	1	22	17
9	190	T3	1	1	1	1	1	#N/B	1	SF	56	1	22	17
9	189	T18	1	1	1	2	1	#N/B	0.796	SF	53	1	4	11
9	189	T3	1	1	1	1	2	#N/B	0.848	SF	53	1	4	11
9	188	T18	1	1	1	1	1	#N/B	1	SF	49	0	8	32
9	188	T3	1	1	1	2	2	#N/B	0.725	SF	49	0	8	32
9	187	T18	2	1	1	2	1	#N/B	0.727	SF	63	0	25	33
9	187	T3	2	1	2	3	2	#N/B	0.088	SF	63	0	25	33
9	186	T18	1	1	1	1	1	#N/B	1	SF	42	1	4	47
9	186	T3	2	1	1	2	1	#N/B	0.727	SF	42	1	4	47
9	185	T18	1	1	1	1	1	#N/B	1	SF	24	1	6	4
9	185	T3	1	1	1	1	1	#N/B	1	SF	24	1	6	4
9	184	T18	1	1	1	2	1	#N/B	0.796	SF	24	1	4	3
9	184	T3	1	1	1	2	1	#N/B	0.796	SF	24	1	4	3

9	183	T18	1	1	1	1	1	#N/B	1	SF	21	1	50	48
9	183	T3	2	1	1	2	2	#N/B	0.656	SF	21	1	50	48
9	182	T18	1	1	1	2	1	#N/B	0.796	SF	33	1	15	19
9	182	T3	1	1	1	2	1	#N/B	0.796	SF	33	1	15	19
9	181	T18	2	1	2	2	2	#N/B	0.62	SF	49	0	25	67
9	181	T3	2	1	2	2	2	#N/B	0.62	SF	49	0	25	67
9	180	T18	1	1	1	1	1	#N/B	1	SF	31	1	10	15
9	180	T3	1	1	1	2	1	#N/B	0.796	SF	31	1	10	15
9	179	T18	1	1	1	1	1	#N/B	1	SF	50	1	25	28
9	179	T3	2	1	1	1	2	#N/B	0.779	SF	50	1	25	28
9	178	T18	1	1	1	2	1	#N/B	0.796	SF	19	0	8	10
9	178	T3	1	1	1	1	1	#N/B	1	SF	19	0	8	10
9	177	T18	1	1	1	1	1	#N/B	1	SF	40	1	3	3
9	177	T3	1	1	1	1	1	#N/B	1	SF	40	1	3	3
9	176	T18	1	1	1	1	1	#N/B	1	SF	50	1	4	6
9	176	T3	1	1	1	2	1	#N/B	0.796	SF	50	1	4	6
9	175	T18	1	1	1	1	1	#N/B	1	SF	44	1	12	8
9	175	T3	1	1	1	2	1	#N/B	0.796	SF	44	1	12	8
9	174	T18	1	1	1	1	1	#N/B	1	SF	21	1	14	22
9	174	T3	1	1	1	1	1	#N/B	1	SF	21	1	14	22
9	173	T18	1	1	1	1	1	#N/B	1	SF	52	1	7.5	15
9	173	T3	1	1	1	1	1	#N/B	1	SF	52	1	7.5	15
9	172	T18	2	1	2	2	1	#N/B	0.691	SF	53	1	5.5	22
9	172	T3	1	1	2	2	1	#N/B	0.76	SF	53	1	5.5	22
9	171	T18	1	1	1	2	2	#N/B	0.725	SF	74	0	5	22
9	171	T3	1	1	1	2	2	#N/B	0.725	SF	74	0	5	22
9	170	T18	1	1	1	1	1	#N/B	1	SF	25	1	36.5	60
9	170	T3	2	1	2	2	2	#N/B	0.62	SF	25	1	36.5	60
9	169	T18	1	1	1	1	1	#N/B	1	SF	38	1	25	60
9	169	T3	2	2	2	1	1	#N/B	0.71	SF	38	1	25	60
9	168	T18	1	1	1	1	1	#N/B	1	SF	23	1	3.5	8
9	168	T3	2	1	1	2	1	#N/B	0.727	SF	23	1	3.5	8
9	167	T3	1	1	2	2	1	#N/B	0.76	SF	49	1	16	33
9	166	T18	1	1	1	2	1	#N/B	0.796	SF	29	1	6.75	19
9	166	T3	1	1	1	2	1	#N/B	0.796	SF	29	1	6.75	19
9	165	T18	1	1	1	1	1	#N/B	1	SF	54	1	16	31
9	165	T3	1	1	2	2	1	#N/B	0.76	SF	54	1	16	31
9	164	T18	1	1	1	2	1	#N/B	0.796	SF	51	1	8	13
9	164	T3	1	1	2	2	1	#N/B	0.76	SF	51	1	8	13
9	163	T18	1	1	1	1	1	#N/B	1	SF	55	1	16	18
9	163	T3	2	1	1	2	1	#N/B	0.727	SF	55	1	16	18
9	162	T18	1	1	1	1	1	#N/B	1	SF	22	0	9	27
9	162	T3	2	1	1	2	1	#N/B	0.727	SF	22	0	9	27
9	161	T18	1	1	1	1	1	#N/B	1	SF	22	1	11	17
9	161	T3	1	1	1	1	1	#N/B	1	SF	22	1	11	17
9	160	T18	1	1	1	1	1	#N/B	1	SF	38	0	1.5	4
9	160	T3	1	1	1	1	1	#N/B	1	SF	38	0	1.5	4
9	159	T18	1	1	1	1	1	#N/B	1	SF	44	1	0.75	8
9	159	T3	1	1	1	2	1	#N/B	0.796	SF	44	1	0.75	8
9	158	T18	1	1	1	1	1	#N/B	1	SF	51	1	7	19
9	158	T3	1	1	1	1	1	#N/B	1	SF	51	1	7	19

9	157	T18	1	1	1	2	1	#N/B	0.796	SF	31	1	4.75	12
9	157	T3	1	1	1	1	1	#N/B	1	SF	31	1	4.75	12
9	156	T3	1	1	1	1	1	#N/B	1	SF	54	1	5	13
9	155	T3	1	1	1	2	1	#N/B	0.796	SF	30	1	32	46
9	154	T3	1	1	1	1	2	#N/B	0.848	SF	65	0	17	24
9	153	T3	1	1	1	2	1	#N/B	0.796	SF	23	1	9	7
9	152	T3	1	1	2	2	3	#N/B	0.255	SF	23	1	20	25
9	151	T3	1	1	1	1	1	#N/B	1	SF	21	1	15	14
9	150	T3	1	1	2	2	1	#N/B	0.76	SF	47	1	30	44
9	149	T3	1	1	1	1	1	#N/B	1	SF	20	1	5	4
9	148	T3	1	1	1	1	1	#N/B	1	SF	56	1	13	24
9	147	T3	1	1	1	1	1	#N/B	1	SF	55	1	8	10
9	146	T3	1	1	1	2	2	#N/B	0.725	SF	22	1	11	13
9	145	T3	1	1	1	1	1	#N/B	1	SF	30	1	5	13
9	144	T3	2	1	2	2	1	#N/B	0.691	SF	47	0	10	20
9	141	T3	1	1	2	2	1	#N/B	0.76	SF	45	1	6	11
9	140	T3	1	1	1	1	2	#N/B	0.848	SF	60	0	15	13
9	139	T3	1	1	1	2	2	#N/B	0.725	SF	55	1	1	8
9	138	T3	1	1	2	1	1	#N/B	0.883	SF	27	1	7	4
9	137	T3	1	1	1	1	1	#N/B	1	SF	30	1	4	7
9	136	T3	1	1	1	1	2	#N/B	0.848	SF	23	1	10	43
9	135	T3	2	1	2	2	1	#N/B	0.691	SF	21	1	4	9
9	134	T3	1	1	1	1	2	#N/B	0.848	SF	19	0	7	25
9	133	T3	1	1	1	1	1	#N/B	1	SF	45	1	5.5	8
9	132	T3	1	1	2	2	1	#N/B	0.76	SF	46	0	39	23
9	131	T3	1	1	2	2	1	#N/B	0.76	SF	51	1	11	18
9	130	T3	1	1	1	2	2	#N/B	0.725	SF	28	1	6	31
9	129	T3	1	1	1	1	2	#N/B	0.848	SF	21	0	3	11
9	128	T3	1	1	1	1	1	#N/B	1	SF	24	1	3	8
9	127	T3	2	1	1	2	1	#N/B	0.727	SF	41	0	1	14
9	126	T3	2	1	1	2	1	#N/B	0.727	SF	51	1	23	58
9	125	T3	1	1	1	1	2	#N/B	0.848	SF	36	1	4	13
9	124	T3	2	1	1	2	1	#N/B	0.727	SF	48	1	3	1
9	121	T3	1	1	1	2	1	#N/B	0.796	SF	36	1	45	81
9	120	T3	1	1	1	2	1	#N/B	0.796	SF	42	1	3	27
9	119	T3	1	1	2	2	2	#N/B	0.689	SF	60	0	10	21
9	118	T3	1	1	1	1	1	#N/B	1	SF	42	1	5	2
9	117	T3	1	1	1	2	1	#N/B	0.796	SF	42	1	11	15
9	116	T3	1	1	3	3	2	#N/B	0.099	SF	27	0	28	28
9	115	T3	1	1	1	2	1	#N/B	0.796	SF	59	1	10	21
9	114	T3	1	1	1	1	1	#N/B	1	SF	48	0	3	25
9	113	T3	2	1	2	2	2	#N/B	0.62	SF	25	0	6	19
9	111	T3	2	1	2	2	2	#N/B	0.62	SF	41	1	8	22
9	110	T3	1	1	2	2	2	#N/B	0.689	SF	53	1	22	43
9	109	T3	2	1	1	1	1	#N/B	0.85	SF	21	1	2.1	12
9	108	T3	1	1	1	1	1	#N/B	1	SF	35	1	11	23
9	107	T3	2	2	2	2	2	#N/B	0.516	SF	37	1	41	111
9	106	T3	1	1	1	1	1	#N/B	1	SF	51	1	7.5	33
9	104	T18	1	1	1	1	1	#N/B	1	SF	45	1	22	19
9	104	T3	1	1	1	1	1	#N/B	1	SF	45	1	22	19
9	103	T18	1	1	1	1	1	#N/B	1	SF	35	0	12	14

9	103	T3	1	1	1	1	2	#N/B	0.848	SF	35	0	12	14
9	102	T18	1	1	1	1	1	#N/B	1	SF	42	1	22	30
9	102	T3	1	1	1	1	1	#N/B	1	SF	42	1	22	30
9	101	T18	1	1	1	2	1	#N/B	0.796	SF	41	1	15	19
9	100	T18	1	1	1	1	1	#N/B	1	SF	43	1	10	7
9	100	T3	1	1	1	1	1	#N/B	1	SF	43	1	10	7
9	99	T18	1	1	1	2	1	#N/B	0.796	SF	37	1	15	22
9	99	T3	1	1	1	2	1	#N/B	0.796	SF	37	1	15	22
9	98	T18	1	1	1	1	1	#N/B	1	SF	19	1	4	5
9	98	T3	1	1	1	1	1	#N/B	1	SF	19	1	4	5
9	97	T18	1	1	1	1	1	#N/B	1	SF	22	1	19	24
9	97	T3	1	1	1	2	1	#N/B	0.796	SF	22	1	19	24
9	96	T18	1	1	1	1	2	#N/B	0.848	SF	48	0	11	28
9	96	T3	1	1	1	1	2	#N/B	0.848	SF	48	0	11	28
9	95	T18	1	1	1	2	1	#N/B	0.796	SF	20	1	20	25
9	95	T3	2	1	2	2	2	#N/B	0.62	SF	20	1	20	25
9	94	T18	1	1	1	2	1	#N/B	0.796	SF	20	1	2.5	4
9	94	T3	1	1	1	2	2	#N/B	0.725	SF	20	1	2.5	4
9	93	T18	1	1	1	1	1	#N/B	1	SF	41	1	6.5	14
9	93	T3	1	1	1	1	1	#N/B	1	SF	41	1	6.5	14
9	92	T18	1	1	1	2	1	#N/B	0.796	SF	40	1	2.75	6
9	92	T3	1	1	1	2	1	#N/B	0.796	SF	40	1	2.75	6
9	91	T18	1	1	1	1	1	#N/B	1	SF	38	0	8	85
9	91	T3	1	1	1	1	1	#N/B	1	SF	38	0	8	85
9	90	T18	1	1	1	2	1	#N/B	0.796	SF	57	0	10	5
9	90	T3	1	1	1	2	1	#N/B	0.796	SF	57	0	10	5
9	89	T18	1	1	1	1	1	#N/B	1	SF	40	1	3	8
9	89	T3	1	1	1	2	1	#N/B	0.796	SF	40	1	3	8
9	88	T18	1	1	1	1	1	#N/B	1	SF	39	1	2	9
9	88	T3	1	1	1	2	1	#N/B	0.796	SF	39	1	2	9
9	87	T18	1	1	1	1	1	#N/B	1	SF	18	1	14	18
9	87	T3	1	1	1	2	2	#N/B	0.725	SF	18	1	14	18
9	86	T18	1	1	1	1	1	#N/B	1	SF	22	1	34	16
9	86	T3	1	1	2	2	3	#N/B	0.255	SF	22	1	34	16
9	85	T18	1	1	1	1	1	#N/B	1	SF	20	0	2	2
9	85	T3	1	1	1	1	3	#N/B	0.414	SF	20	0	2	2
9	84	T18	1	1	1	2	1	#N/B	0.796	SF	60	1	27	30
9	84	T3	1	1	1	1	1	#N/B	1	SF	60	1	27	30
9	83	T18	1	1	1	1	2	#N/B	0.848	SF	62	0	18	12
9	83	T3	1	1	1	1	1	#N/B	1	SF	62	0	18	12
9	82	T18	1	1	1	2	1	#N/B	0.796	SF	58	0	11	21
9	82	T3	1	1	1	2	1	#N/B	0.796	SF	58	0	11	21
9	81	T18	1	1	1	1	1	#N/B	1	SF	21	1	7.5	6
9	81	T3	1	1	1	1	1	#N/B	1	SF	21	1	7.5	6
9	80	T18	1	1	1	1	1	#N/B	1	SF	48	1	6	8
9	80	T3	1	1	1	1	1	#N/B	1	SF	48	1	6	8
9	79	T18	1	1	1	2	1	#N/B	0.796	SF	24	1	3.5	7
9	79	T3	1	1	1	2	2	#N/B	0.725	SF	24	1	3.5	7
9	78	T18	2	1	1	1	1	#N/B	0.85	SF	34	1	10	23
9	77	T18	1	1	1	1	1	#N/B	1	SF	28	1	9	13
9	77	T3	1	1	1	1	1	#N/B	1	SF	28	1	9	13

9	76	T18	1	1	1	2	1	#N/B	0.796	SF	44	1	5	4
9	76	T3	1	1	1	1	2	#N/B	0.848	SF	44	1	5	4
9	75	T18	1	1	1	1	1	#N/B	1	SF	34	1	9	28
9	75	T3	1	1	1	2	1	#N/B	0.796	SF	34	1	9	28
9	74	T18	1	1	1	1	1	#N/B	1	SF	65	1	18	11
9	74	T3	1	1	1	1	1	#N/B	1	SF	65	1	18	11
9	73	T18	1	1	1	1	1	#N/B	1	SF	40	1	8	4
9	73	T3	1	1	1	2	2	#N/B	0.725	SF	40	1	8	4
9	72	T18	1	1	1	1	1	#N/B	1	SF	34	1	1	2
9	72	T3	1	1	1	1	1	#N/B	1	SF	34	1	1	2
9	71	T18	1	1	1	2	1	#N/B	0.796	SF	64	1	30	43
9	71	T3	1	1	1	1	1	#N/B	1	SF	64	1	30	43
9	70	T18	1	1	1	1	2	#N/B	0.848	SF	20	1	4	5
9	70	T3	1	1	1	1	1	#N/B	1	SF	20	1	4	5
9	69	T18	1	1	1	1	1	#N/B	1	SF	53	1	5	15
9	69	T3	1	1	1	1	1	#N/B	1	SF	53	1	5	15
9	68	T18	1	1	1	2	1	#N/B	0.796	SF	33	1	6	19
9	68	T3	1	1	1	1	1	#N/B	1	SF	33	1	6	19
9	67	T18	1	1	1	1	1	#N/B	1	SF	36	1	11	19
9	67	T3	1	1	1	1	1	#N/B	1	SF	36	1	11	19
9	66	T18	1	1	1	1	1	#N/B	1	SF	46	1	17	21
9	66	T3	1	1	1	2	1	#N/B	0.796	SF	46	1	17	21
9	65	T18	1	1	1	1	1	#N/B	1	SF	45	1	6	18
9	65	T3	1	1	1	1	1	#N/B	1	SF	45	1	6	18
9	64	T18	1	1	1	1	1	#N/B	1	SF	64	1	12	20
9	64	T3	1	1	1	1	2	#N/B	0.848	SF	64	1	12	20
9	63	T18	1	1	1	1	1	#N/B	1	SF	20	1	6	6
9	63	T3	1	1	1	1	1	#N/B	1	SF	20	1	6	6
9	62	T18	1	1	1	1	1	#N/B	1	SF	54	1	4.5	18
9	62	T3	1	1	1	1	1	#N/B	1	SF	54	1	4.5	18
9	61	T18	1	1	1	1	1	#N/B	1	SF	39	0	1.5	9
9	61	T3	1	1	1	1	2	#N/B	0.848	SF	39	0	1.5	9
9	60	T3	2	1	2	2	2	#N/B	0.62	SF	27	0	21	25
9	59	T3	1	1	1	1	1	#N/B	1	SF	39	1	2	5
9	58	T3	2	1	1	2	2	#N/B	0.656	SF	35	1	6	38
9	57	T3	1	1	1	1	1	#N/B	1	SF	35	1	2	5
9	56	T3	1	1	1	1	2	#N/B	0.848	SF	28	1	9	2
9	55	T3	1	1	1	1	1	#N/B	1	SF	54	1	3	5
9	54	T3	2	1	2	1	1	#N/B	0.814	SF	58	1	10	26
9	53	T3	1	1	1	1	1	#N/B	1	SF	25	1	8	32
9	52	T3	1	1	1	2	1	#N/B	0.796	SF	34	0	7	20
9	50	T3	1	1	1	1	1	#N/B	1	SF	23	0	2	2
9	49	T3	1	1	1	1	1	#N/B	1	SF	36	1	7	7
9	48	T3	1	1	2	2	2	#N/B	0.689	SF	20	0	8	8
9	46	T3	1	1	1	1	1	#N/B	1	SF	39	1	10.5	9
9	45	T3	1	1	1	1	1	#N/B	1	SF	37	1	9.45	21
9	44	T3	1	1	1	1	3	#N/B	0.414	SF	19	1	5	21
9	43	T18	1	1	1	1	2	#N/B	0.848	SF	20	1	9	9
9	43	T3	1	1	1	1	2	#N/B	0.848	SF	20	1	9	9
9	42	T18	1	1	1	2	1	#N/B	0.796	SF	44	1	3	8
9	42	T3	1	1	1	1	1	#N/B	1	SF	44	1	3	8

9	41	T18	1	1	1	2	1	#N/B	0.796	SF	45	1	13	84
9	41	T3	1	1	1	1	1	#N/B	1	SF	45	1	13	84
9	40	T18	1	1	1	2	2	#N/B	0.725	SF	60	0	16	27
9	40	T3	2	1	1	2	1	#N/B	0.727	SF	60	0	16	27
9	39	T18	1	1	1	1	2	#N/B	0.848	SF	40	1	0.5	15
9	39	T3	1	1	1	2	1	#N/B	0.796	SF	40	1	0.5	15
9	38	T18	1	1	1	1	1	#N/B	1	SF	19	1	10	8
9	38	T3	1	1	1	1	1	#N/B	1	SF	19	1	10	8
9	37	T18	1	1	1	1	1	#N/B	1	SF	21	1	8	15
9	37	T3	1	1	1	1	1	#N/B	1	SF	21	1	8	15
9	36	T18	2	1	2	2	2	#N/B	0.62	SF	44	1	12	11
9	36	T3	1	1	2	2	2	#N/B	0.689	SF	44	1	12	11
9	35	T18	1	1	1	1	1	#N/B	1	SF	62	1	23.5	31
9	35	T3	1	1	1	1	1	#N/B	1	SF	62	1	23.5	31
9	34	T18	1	1	1	2	1	#N/B	0.796	SF	60	1	16	29
9	34	T3	1	1	1	1	2	#N/B	0.848	SF	60	1	16	29
9	33	T18	1	1	1	1	1	#N/B	1	SF	59	1	5	8
9	33	T3	1	1	1	1	1	#N/B	1	SF	59	1	5	8
9	32	T18	1	1	1	1	1	#N/B	1	SF	25	1	2	6
9	32	T3	1	1	1	1	1	#N/B	1	SF	25	1	2	6
9	31	T18	1	1	2	2	1	#N/B	0.76	SF	58	1	15	26
9	31	T3	1	1	2	2	1	#N/B	0.76	SF	58	1	15	26
9	30	T18	1	1	1	1	1	#N/B	1	SF	18	1	9	5
9	30	T3	2	1	1	1	1	#N/B	0.85	SF	18	1	9	5
9	29	T18	1	1	1	1	1	#N/B	1	SF	38	1	25	9
9	29	T3	1	1	1	1	1	#N/B	1	SF	38	1	25	9
9	28	T18	1	1	1	1	1	#N/B	1	SF	20	1	17	23
9	28	T3	1	1	1	1	1	#N/B	1	SF	20	1	17	23
9	27	T18	1	1	1	1	1	#N/B	1	SF	23	1	19.75	43
9	27	T3	1	1	1	1	1	#N/B	1	SF	23	1	19.75	43
9	26	T18	1	1	1	1	1	#N/B	1	SF	18	1	6	10
9	26	T3	1	1	1	1	1	#N/B	1	SF	18	1	6	10
9	25	T18	1	1	1	1	1	#N/B	1	SF	32	1	43	46
9	25	T3	1	1	1	1	1	#N/B	1	SF	32	1	43	46
9	24	T18	1	1	1	1	1	#N/B	1	SF	18	1	4.5	19
9	24	T3	1	1	1	1	1	#N/B	1	SF	18	1	4.5	19
9	23	T3	1	1	1	1	1	#N/B	1	SF	21	1	14	13
9	22	T3	1	1	1	1	1	#N/B	1	SF	56	1	5	10
9	21	T3	1	1	1	1	1	#N/B	1	SF	47	1	50	37
9	20	T3	1	1	1	1	1	#N/B	1	SF	24	1	14	20
9	19	T3	1	1	1	1	1	#N/B	1	SF	40	1	3	7
9	18	T3	1	1	1	1	1	#N/B	1	SF	20	1	14	11
9	17	T3	1	1	1	1	1	#N/B	1	SF	23	1	6	15
9	16	T3	1	1	1	2	2	#N/B	0.725	SF	60	1	28	37
9	15	T3	1	1	2	2	2	#N/B	0.689	SF	37	1	4	30
9	14	T3	1	1	1	1	1	#N/B	1	SF	53	0	1	14
9	13	T3	1	1	1	1	1	#N/B	1	SF	43	1	1.5	2
9	12	T18	2	1	2	2	1	#N/B	0.691	SF	54	0	1	4
9	11	T18	2	1	2	2	2	#N/B	0.62	SF	49	0	21	32
9	10	T18	1	1	1	2	1	#N/B	0.796	SF	32	1	15	25
9	9	T18	1	1	1	1	1	#N/B	1	SF	35	1	10	13

9	8	T18	1	1	1	1	1	#N/B	1	SF	44	0	15	21
9	7	T18	1	1	1	2	1	#N/B	0.796	SF	55	1	9	17
9	6	T18	1	1	1	1	1	#N/B	1	SF	50	1	12	25
9	5	T18	1	1	1	1	1	#N/B	1	SF	55	0	8.75	20
9	4	T18	1	1	1	1	1	#N/B	1	SF	36	1	6	6
9	3	T18	1	1	1	1	1	#N/B	1	SF	21	1	30	28
9	2	T18	1	1	1	1	1	#N/B	1	SF	48	0	3	3
9	1	T18	1	1	1	1	1	#N/B	1	SF	20	0	1	3
10	150	T0.5	2	2	2	2	1	60	0.587	EQ	21	0	12	47
10	149	T0.5	2	2	3	1	1	60	0.383	EQ	69	0	30	58
10	148	T0.5	1	1	2	2	1	40	0.76	EQ	50	1	7	12
10	147	T0.5	1	1	1	1	1	80	1	EQ	31	1	11	7
10	146	T0.5	1	1	2	2	1	80	0.76	EQ	27	1	30	10
10	145	T0.5	1	1	1	2	1	60	0.796	EQ	25	0	10	14
10	144	T0.5	3	2	3	2	2	40	-0.056	EQ	71	1	8	45
10	143	T0.5	1	1	1	1	1	95	1	EQ	19	1	6	6
10	142	T0.5	1	1	2	2	1	85	0.76	EQ	63	0	4	11
10	142	T3	1	1	1	2	1	85	0.796	EQ	63	0	4	11
10	141	T0.5	2	1	2	2	2	90	0.62	EQ	18	0	10	11
10	140	T0.5	1	1	3	2	2	86	0.362	EQ	51	0	5	5
10	138	T0.5	1	1	1	1	1	80	1	EQ	47	1	6	3
10	138	T12	1	1	1	1	1	85	1	EQ	47	1	6	3
10	138	T18	1	1	1	1	1	80	1	EQ	47	1	6	3
10	138	T3	1	1	1	1	1	88	1	EQ	47	1	6	3
10	138	T6	1	1	1	1	1	90	1	EQ	47	1	6	3
10	137	T0.5	1	1	2	1	1	75	0.883	EQ	32	1	5	9
10	137	T12	1	1	1	1	1	95	1	EQ	32	1	5	9
10	137	T18	1	1	1	1	1	98	1	EQ	32	1	5	9
10	137	T3	1	1	1	1	1	95	1	EQ	32	1	5	9
10	137	T6	1	1	1	1	1	100	1	EQ	32	1	5	9
10	136	T0.5	1	1	2	1	1	76	0.883	EQ	20	1	2	7
10	136	T12	1	1	1	1	1	98	1	EQ	20	1	2	7
10	136	T18	1	1	1	1	1	98	1	EQ	20	1	2	7
10	136	T3	1	1	1	1	1	88	1	EQ	20	1	2	7
10	136	T6	1	1	1	1	1	95	1	EQ	20	1	2	7
10	135	T0.5	1	1	1	1	1	90	1	EQ	60	1	2	8
10	135	T12	1	1	1	1	1	98	1	EQ	60	1	2	8
10	135	T18	1	1	1	1	1	98	1	EQ	60	1	2	8
10	135	T3	1	1	1	1	1	90	1	EQ	60	1	2	8
10	135	T6	1	1	1	1	1	95	1	EQ	60	1	2	8
10	134	T0.5	2	2	2	2	1	50	0.587	EQ	43	1	6	11
10	134	T12	1	1	1	1	1	100	1	EQ	43	1	6	11
10	134	T18	1	1	1	1	1	85	1	EQ	43	1	6	11
10	134	T3	1	1	1	1	1	100	1	EQ	43	1	6	11
10	134	T6	1	1	1	1	1	100	1	EQ	43	1	6	11
10	132	T0.5	1	2	2	2	1	90	0.656	EQ	31	1	5	5
10	132	T12	1	1	1	1	1	100	1	EQ	31	1	5	5
10	132	T18	1	1	1	1	1	97	1	EQ	31	1	5	5
10	132	T3	1	1	1	1	1	97	1	EQ	31	1	5	5
10	132	T6	1	1	1	1	1	97	1	EQ	31	1	5	5
10	131	T0.5	2	2	2	2	3	28	0.082	EQ	48	1	4	12

10	131	T12	1	1	1	1	2	80	0.848	EQ	48	1	4	12
10	131	T18	1	1	1	1	2	70	0.848	EQ	48	1	4	12
10	131	T3	2	1	1	2	2	65	0.656	EQ	48	1	4	12
10	131	T6	1	1	1	1	2	80	0.848	EQ	48	1	4	12
10	130	T0.5	1	2	2	2	1	95	0.656	EQ	51	1	12	12
10	130	T12	1	1	1	1	1	99	1	EQ	51	1	12	12
10	130	T18	1	1	1	1	1	100	1	EQ	51	1	12	12
10	130	T3	1	1	1	1	1	100	1	EQ	51	1	12	12
10	130	T6	1	1	1	1	1	100	1	EQ	51	1	12	12
10	129	T0.5	1	2	2	2	1	80	0.656	EQ	41	1	12	22
10	129	T12	1	1	1	1	1	98	1	EQ	41	1	12	22
10	129	T18	1	1	1	1	1	100	1	EQ	41	1	12	22
10	129	T3	1	1	1	1	1	92	1	EQ	41	1	12	22
10	129	T6	1	1	1	1	1	90	1	EQ	41	1	12	22
10	127	T0.5	1	1	1	1	1	83	1	EQ	40	1	9	6
10	127	T12	1	1	1	1	1	95	1	EQ	40	1	9	6
10	127	T18	1	1	1	1	1	98	1	EQ	40	1	9	6
10	127	T3	1	1	1	1	1	80	1	EQ	40	1	9	6
10	127	T6	1	1	1	1	1	85	1	EQ	40	1	9	6
10	125	T0.5	2	2	2	2	1	90	0.587	EQ	58	1	6	24
10	125	T12	2	1	2	1	1	80	0.814	EQ	58	1	6	24
10	125	T18	1	1	1	1	1	80	1	EQ	58	1	6	24
10	125	T3	2	2	2	2	1	70	0.587	EQ	58	1	6	24
10	125	T6	2	2	2	2	1	70	0.587	EQ	58	1	6	24
10	123	T0.5	1	1	3	1	2	60	0.485	EQ	39	0	5	29
10	123	T12	1	1	1	1	2	80	0.848	EQ	39	0	5	29
10	123	T18	1	1	1	1	1	90	1	EQ	39	0	5	29
10	123	T3	2	1	2	2	1	70	0.691	EQ	39	0	5	29
10	123	T6	1	1	2	2	2	75	0.689	EQ	39	0	5	29
10	122	T0.5	1	2	2	1	1	85	0.779	EQ	32	1	8	8
10	122	T12	1	1	1	1	1	98	1	EQ	32	1	8	8
10	122	T18	1	1	1	1	1	95	1	EQ	32	1	8	8
10	122	T3	1	1	1	1	1	95	1	EQ	32	1	8	8
10	122	T6	1	1	1	1	1	98	1	EQ	32	1	8	8
10	121	T0.5	3	3	3	2	2	70	-0.166	EQ	54	0	15	24
10	121	T12	1	1	1	1	1	75	1	EQ	54	0	15	24
10	121	T18	1	1	1	1	1	75	1	EQ	54	0	15	24
10	121	T3	1	2	1	1	1	80	0.815	EQ	54	0	15	24
10	121	T6	1	1	2	2	1	65	0.76	EQ	54	0	15	24
10	120	T0.5	1	1	1	1	1	90	1	EQ	20	1	13	16
10	120	T12	1	1	1	1	1	90	1	EQ	20	1	13	16
10	120	T18	1	1	1	1	1	100	1	EQ	20	1	13	16
10	120	T3	1	1	1	1	1	100	1	EQ	20	1	13	16
10	120	T6	1	1	1	1	1	95	1	EQ	20	1	13	16
10	119	T0.5	1	1	1	1	1	70	1	EQ	54	1	7	11
10	119	T12	1	1	1	1	1	98	1	EQ	54	1	7	11
10	119	T18	1	1	1	1	1	100	1	EQ	54	1	7	11
10	119	T3	1	1	1	1	1	100	1	EQ	54	1	7	11
10	119	T6	1	1	1	1	1	95	1	EQ	54	1	7	11
10	118	T0.5	2	1	2	2	1	80	0.691	EQ	46	1	18	21
10	118	T12	1	1	1	1	1	80	1	EQ	46	1	18	21

10	118	T18	1	1	1	2	1	80	0.796	EQ	46	1	18	21
10	118	T3	1	1	1	1	2	82	0.848	EQ	46	1	18	21
10	118	T6	1	1	1	1	1	80	1	EQ	46	1	18	21
10	117	T0.5	1	1	2	2	1	85	0.76	EQ	60	0	6	13
10	117	T12	1	1	1	1	1	85	1	EQ	60	0	6	13
10	117	T18	1	1	1	1	1	95	1	EQ	60	0	6	13
10	117	T3	1	1	2	2	1	80	0.76	EQ	60	0	6	13
10	117	T6	1	1	1	1	1	84	1	EQ	60	0	6	13
10	116	T0.5	1	1	1	1	1	60	1	EQ	54	1	7	11
10	116	T3	1	1	2	2	2	60	0.689	EQ	54	1	7	11
10	116	T6	2	2	3	3	3	40	-0.239	EQ	54	1	7	11
10	115	T0.5	1	1	1	1	1	90	1	EQ	18	1	8	5
10	115	T3	1	1	1	1	1	100	1	EQ	18	1	8	5
10	114	T0.5	2	2	2	3	2	40	-0.016	EQ	36	0	3	10
10	113	T0.5	1	1	2	2	1	80	0.76	EQ	52	1	3	6
10	113	T12	1	1	1	1	1	85	1	EQ	52	1	3	6
10	113	T18	1	1	1	1	1	90	1	EQ	52	1	3	6
10	113	T3	1	1	1	1	1	75	1	EQ	52	1	3	6
10	113	T6	1	1	1	1	1	80	1	EQ	52	1	3	6
10	112	T0.5	3	2	3	2	1	40	0.015	EQ	44	1	5	21
10	112	T12	1	1	1	1	1	88	1	EQ	44	1	5	21
10	112	T18	1	1	1	1	1	92	1	EQ	44	1	5	21
10	112	T3	1	1	1	1	1	95	1	EQ	44	1	5	21
10	112	T6	1	1	1	1	1	90	1	EQ	44	1	5	21
10	111	T0.5	1	1	2	1	1	70	0.883	EQ	22	1	2	2
10	109	T0.5	1	1	1	1	1	85	1	EQ	35	0	3	5
10	109	T12	1	1	1	2	1	65	0.796	EQ	35	0	3	5
10	109	T18	1	1	1	1	1	90	1	EQ	35	0	3	5
10	109	T3	2	1	1	2	1	28	0.727	EQ	35	0	3	5
10	109	T6	1	1	1	2	1	75	0.796	EQ	35	0	3	5
10	108	T0.5	1	1	1	1	1	90	1	EQ	32	1	2.5	2
10	108	T12	1	1	1	1	1	65	1	EQ	32	1	2.5	2
10	108	T18	1	1	1	1	1	80	1	EQ	32	1	2.5	2
10	108	T3	1	1	1	1	1	80	1	EQ	32	1	2.5	2
10	108	T6	1	1	1	1	1	90	1	EQ	32	1	2.5	2
10	107	T0.5	1	1	2	1	1	80	0.883	EQ	47	1	11	13
10	107	T12	1	1	1	2	1	85	0.796	EQ	47	1	11	13
10	107	T18	1	1	1	1	1	100	1	EQ	47	1	11	13
10	107	T3	1	1	2	2	1	80	0.76	EQ	47	1	11	13
10	107	T6	1	1	2	2	1	75	0.76	EQ	47	1	11	13
10	106	T0.5	1	2	2	1	2	85	0.708	EQ	55	1	7	11
10	106	T12	1	1	1	1	1	70	1	EQ	55	1	7	11
10	106	T18	1	1	1	1	1	70	1	EQ	55	1	7	11
10	106	T3	1	1	2	2	2	70	0.689	EQ	55	1	7	11
10	105	T0.5	1	1	1	1	2	50	0.848	EQ	45	0	7	10
10	105	T12	1	1	1	1	2	#N/B	0.848	EQ	45	0	7	10
10	105	T18	1	1	1	1	2	50	0.848	EQ	45	0	7	10
10	105	T3	1	1	1	1	1	50	1	EQ	45	0	7	10
10	105	T6	1	1	1	1	2	#N/B	0.848	EQ	45	0	7	10
10	102	T0.5	1	1	1	1	1	100	1	EQ	42	1	5	12
10	102	T12	1	1	1	1	1	100	1	EQ	42	1	5	12

10	102	T18	1	1	1	1	1	100	1	EQ	42	1	5	12
10	102	T3	1	1	1	1	2	100	0.848	EQ	42	1	5	12
10	102	T6	1	1	1	1	1	100	1	EQ	42	1	5	12
10	101	T0.5	2	1	2	1	1	95	0.814	EQ	48	1	8	7
10	101	T12	1	1	1	1	1	100	1	EQ	48	1	8	7
10	101	T18	1	1	1	1	1	100	1	EQ	48	1	8	7
10	101	T3	1	1	1	1	1	100	1	EQ	48	1	8	7
10	101	T6	1	1	1	1	1	100	1	EQ	48	1	8	7
10	100	T0.5	2	3	3	2	2	58	0.079	EQ	49	1	30	76
10	100	T12	1	1	2	1	1	70	0.883	EQ	49	1	30	76
10	100	T18	1	1	2	1	1	85	0.883	EQ	49	1	30	76
10	100	T3	2	2	1	1	1	70	0.746	EQ	49	1	30	76
10	100	T6	1	1	2	1	1	85	0.883	EQ	49	1	30	76
10	99	T0.5	2	1	2	2	1	75	0.691	EQ	41	1	5	11
10	99	T3	1	1	1	1	1	95	1	EQ	41	1	5	11
10	98	T0.5	1	1	1	2	1	80	0.796	EQ	28	0	2	8
10	98	T12	1	1	1	1	1	100	1	EQ	28	0	2	8
10	98	T18	1	1	1	1	1	100	1	EQ	28	0	2	8
10	98	T3	1	1	1	1	1	97	1	EQ	28	0	2	8
10	98	T6	1	1	1	1	1	95	1	EQ	28	0	2	8
10	97	T0.5	1	1	1	1	1	95	1	EQ	24	1	8	6
10	97	T12	1	1	1	1	1	98	1	EQ	24	1	8	6
10	97	T18	1	1	1	1	1	100	1	EQ	24	1	8	6
10	97	T3	1	1	1	1	1	92	1	EQ	24	1	8	6
10	96	T0.5	1	1	2	2	1	85	0.76	EQ	44	0	7	7
10	96	T12	1	1	1	1	1	95	1	EQ	44	0	7	7
10	96	T18	1	1	1	1	1	95	1	EQ	44	0	7	7
10	96	T3	1	1	2	2	1	90	0.76	EQ	44	0	7	7
10	96	T6	1	1	1	1	1	88	1	EQ	44	0	7	7
10	95	T0.5	1	2	2	2	1	50	0.656	EQ	45	1	6	11
10	95	T12	1	1	1	2	2	60	0.725	EQ	45	1	6	11
10	95	T18	1	1	1	2	2	60	0.725	EQ	45	1	6	11
10	95	T3	1	1	1	2	1	90	0.796	EQ	45	1	6	11
10	95	T6	1	1	1	1	1	80	1	EQ	45	1	6	11
10	94	T0.5	2	2	3	2	1	60	0.26	EQ	35	1	10	13
10	94	T12	1	1	1	1	1	100	1	EQ	35	1	10	13
10	94	T18	1	1	1	1	1	90	1	EQ	35	1	10	13
10	94	T3	1	1	1	1	2	70	0.848	EQ	35	1	10	13
10	94	T6	1	1	2	2	1	70	0.76	EQ	35	1	10	13
10	93	T0.5	2	2	3	2	1	60	0.26	EQ	25	1	16	18
10	93	T12	1	1	1	2	1	80	0.796	EQ	25	1	16	18
10	93	T18	1	1	2	1	1	80	0.883	EQ	25	1	16	18
10	93	T3	1	1	2	2	1	60	0.76	EQ	25	1	16	18
10	93	T6	1	1	1	2	1	60	0.796	EQ	25	1	16	18
10	92	T0.5	2	2	3	2	3	40	0.024	EQ	30	1	3	9
10	92	T12	2	2	2	2	3	40	0.082	EQ	30	1	3	9
10	92	T18	2	2	3	3	2	68	-0.074	EQ	30	1	3	9
10	92	T3	1	2	3	3	2	70	-0.005	EQ	30	1	3	9
10	92	T6	2	2	2	2	3	55	0.082	EQ	30	1	3	9
10	91	T0.5	3	3	3	2	1	40	-0.095	EQ	56	1	8	56
10	91	T12	1	1	1	1	1	90	1	EQ	56	1	8	56

10	91	T18	1	1	1	1	1	90	1	EQ	56	1	8	56
10	91	T3	2	1	3	2	1	70	0.364	EQ	56	1	8	56
10	91	T6	2	1	2	2	1	70	0.691	EQ	56	1	8	56
10	90	T0.5	1	1	1	1	1	80	1	EQ	56	1	21	23
10	90	T12	1	1	2	1	1	80	0.883	EQ	56	1	21	23
10	90	T18	1	1	1	1	1	80	1	EQ	56	1	21	23
10	90	T3	1	1	3	1	2	50	0.485	EQ	56	1	21	23
10	90	T6	1	1	2	1	2	70	0.812	EQ	56	1	21	23
10	89	T0.5	1	1	2	3	2	40	0.157	EQ	65	0	8	33
10	89	T12	1	1	1	1	1	80	1	EQ	65	0	8	33
10	89	T18	1	1	2	1	1	80	0.883	EQ	65	0	8	33
10	89	T3	1	1	2	2	2	70	0.689	EQ	65	0	8	33
10	89	T6	1	1	2	2	1	70	0.76	EQ	65	0	8	33
10	88	T0.5	1	1	1	1	1	40	1	EQ	60	0	8	14
10	88	T12	1	1	1	1	1	90	1	EQ	60	0	8	14
10	88	T18	1	1	1	1	1	90	1	EQ	60	0	8	14
10	88	T3	1	1	1	1	1	80	1	EQ	60	0	8	14
10	88	T6	1	1	1	1	1	90	1	EQ	60	0	8	14
10	87	T0.5	3	3	3	2	2	60	-0.166	EQ	34	0	6	20
10	87	T12	2	1	2	3	3	30	-0.077	EQ	34	0	6	20
10	87	T18	2	1	2	3	3	50	-0.077	EQ	34	0	6	20
10	87	T3	1	1	2	2	1	20	0.76	EQ	34	0	6	20
10	87	T6	2	1	1	3	3	35	-0.041	EQ	34	0	6	20
10	85	T0.5	2	2	3	2	2	65	0.189	EQ	51	1	3	19
10	85	T12	1	1	1	1	2	50	0.848	EQ	51	1	3	19
10	85	T18	1	1	1	1	3	20	0.414	EQ	51	1	3	19
10	85	T3	2	1	2	2	2	30	0.62	EQ	51	1	3	19
10	85	T6	1	1	1	1	2	72	0.848	EQ	51	1	3	19
10	84	T0.5	3	2	3	1	1	100	0.138	EQ	34	1	1.5	12
10	84	T12	1	1	1	1	1	100	1	EQ	34	1	1.5	12
10	84	T3	1	1	1	1	1	98	1	EQ	34	1	1.5	12
10	84	T6	1	1	1	1	1	100	1	EQ	34	1	1.5	12
10	83	T0.5	1	1	1	1	1	80	1	EQ	18	0	1.5	5
10	83	T3	1	1	1	1	1	85	1	EQ	18	0	1.5	5
10	82	T0.5	3	2	2	2	2	84	0.002	EQ	23	1	7.7	26
10	82	T6	1	1	1	1	1	75	1	EQ	23	1	7.7	26
10	81	T0.5	1	1	1	1	1	100	1	EQ	35	1	3.6	16
10	81	T12	1	1	1	1	1	100	1	EQ	35	1	3.6	16
10	81	T18	1	1	1	1	1	100	1	EQ	35	1	3.6	16
10	81	T3	1	1	1	1	1	100	1	EQ	35	1	3.6	16
10	81	T6	1	1	1	1	1	100	1	EQ	35	1	3.6	16
10	80	T0.5	2	2	3	2	2	40	0.189	EQ	62	0	3.4	26
10	80	T12	1	1	1	1	1	100	1	EQ	62	0	3.4	26
10	80	T18	1	1	1	1	1	99	1	EQ	62	0	3.4	26
10	80	T3	1	1	1	2	1	98	0.796	EQ	62	0	3.4	26
10	80	T6	1	1	1	1	1	100	1	EQ	62	0	3.4	26
10	79	T0.5	3	2	3	2	1	99	0.015	EQ	48	0	1.6	14
10	79	T12	2	1	2	2	1	95	0.691	EQ	48	0	1.6	14
10	79	T18	2	1	2	1	1	94	0.814	EQ	48	0	1.6	14
10	79	T3	2	1	2	1	1	90	0.814	EQ	48	0	1.6	14
10	79	T6	2	1	2	2	1	95	0.691	EQ	48	0	1.6	14

10	78	T0.5	1	1	1	2	1	85	0.796	EQ	21	1	10	9
10	78	T12	1	1	1	2	2	85	0.725	EQ	21	1	10	9
10	78	T3	1	1	2	2	1	90	0.76	EQ	21	1	10	9
10	78	T6	1	1	1	1	1	80	1	EQ	21	1	10	9
10	77	T0.5	1	1	1	2	1	80	0.796	EQ	18	1	7.3	24
10	77	T18	1	1	2	1	1	95	0.883	EQ	18	1	7.3	24
10	77	T3	1	1	2	1	2	80	0.812	EQ	18	1	7.3	24
10	77	T6	1	1	1	1	1	90	1	EQ	18	1	7.3	24
10	76	T0.5	2	1	2	2	1	80	0.691	EQ	57	1	1.5	11
10	76	T12	1	1	1	3	1	79	0.264	EQ	57	1	1.5	11
10	76	T18	1	1	1	2	1	70	0.796	EQ	57	1	1.5	11
10	76	T6	2	1	2	2	1	75	0.691	EQ	57	1	1.5	11
10	75	T0.5	1	1	1	1	1	80	1	EQ	49	0	4.2	27
10	75	T12	1	1	1	1	1	100	1	EQ	49	0	4.2	27
10	75	T18	1	1	1	1	1	85	1	EQ	49	0	4.2	27
10	75	T3	1	1	1	1	1	90	1	EQ	49	0	4.2	27
10	75	T6	1	1	1	1	1	90	1	EQ	49	0	4.2	27
10	74	T0.5	3	2	2	2	2	73	0.002	EQ	46	1	1	18
10	74	T12	2	1	1	1	1	99	0.85	EQ	46	1	1	18
10	74	T3	1	1	2	1	2	94	0.812	EQ	46	1	1	18
10	72	T0.5	1	1	1	1	1	99	1	EQ	36	1	17.5	37
10	72	T12	1	1	1	1	1	100	1	EQ	36	1	17.5	37
10	72	T18	1	1	1	1	1	95	1	EQ	36	1	17.5	37
10	72	T3	1	1	1	1	1	80	1	EQ	36	1	17.5	37
10	72	T6	1	1	1	1	1	90	1	EQ	36	1	17.5	37
10	71	T0.5	1	1	2	2	2	70	0.689	EQ	20	1	1	4
10	71	T12	1	1	1	1	1	63	1	EQ	20	1	1	4
10	71	T3	1	1	1	2	1	65	0.796	EQ	20	1	1	4
10	71	T6	1	1	1	2	1	70	0.796	EQ	20	1	1	4
10	70	T0.5	3	2	3	2	1	81	0.015	EQ	24	1	5.6	28
10	70	T12	1	1	1	2	1	100	0.796	EQ	24	1	5.6	28
10	70	T18	1	1	1	1	1	98	1	EQ	24	1	5.6	28
10	70	T3	2	1	1	2	1	90	0.727	EQ	24	1	5.6	28
10	70	T6	1	1	1	2	1	94	0.796	EQ	24	1	5.6	28
10	69	T0.5	1	1	1	1	1	100	1	EQ	30	1	6.5	7
10	69	T12	1	1	1	2	2	71	0.725	EQ	30	1	6.5	7
10	69	T18	1	1	1	1	1	90	1	EQ	30	1	6.5	7
10	69	T3	1	1	1	2	2	90	0.725	EQ	30	1	6.5	7
10	69	T6	1	1	1	2	2	90	0.725	EQ	30	1	6.5	7
10	68	T0.5	2	1	3	2	1	51	0.364	EQ	31	1	8.7	13
10	68	T18	1	1	1	2	1	94	0.796	EQ	31	1	8.7	13
10	68	T3	1	2	2	2	1	93	0.656	EQ	31	1	8.7	13
10	67	T0.5	1	1	2	2	1	65	0.76	EQ	58	0	2.3	25
10	67	T12	1	1	1	1	1	90	1	EQ	58	0	2.3	25
10	67	T18	1	1	1	1	1	80	1	EQ	58	0	2.3	25
10	67	T3	1	1	1	1	1	70	1	EQ	58	0	2.3	25
10	67	T6	1	1	1	1	1	70	1	EQ	58	0	2.3	25
10	66	T0.5	3	1	3	2	2	69	0.048	EQ	18	0	4.5	18
10	66	T12	1	1	1	1	1	100	1	EQ	18	0	4.5	18
10	66	T18	1	1	1	2	1	96	0.796	EQ	18	0	4.5	18
10	66	T6	2	1	1	2	1	95	0.727	EQ	18	0	4.5	18

10	65	T0.5	2	2	3	2	1	78	0.26	EQ	20	1	6.5	14
10	65	T18	1	1	1	1	1	80	1	EQ	20	1	6.5	14
10	65	T3	1	1	2	1	1	75	0.883	EQ	20	1	6.5	14
10	65	T6	1	1	1	1	1	80	1	EQ	20	1	6.5	14
10	64	T0.5	1	1	2	2	1	68	0.76	EQ	27	0	8.6	8
10	64	T12	1	1	1	1	1	85	1	EQ	27	0	8.6	8
10	64	T18	1	1	1	1	1	100	1	EQ	27	0	8.6	8
10	64	T3	1	1	1	1	1	75	1	EQ	27	0	8.6	8
10	64	T6	1	1	1	1	1	82	1	EQ	27	0	8.6	8
10	63	T0.5	2	2	1	2	2	42	0.552	EQ	20	0	9.7	23
10	63	T12	1	1	2	2	2	65	0.689	EQ	20	0	9.7	23
10	63	T18	1	1	1	2	2	65	0.725	EQ	20	0	9.7	23
10	63	T3	1	1	2	2	2	70	0.689	EQ	20	0	9.7	23
10	63	T6	1	1	2	2	1	70	0.76	EQ	20	0	9.7	23
10	62	T0.5	1	1	3	2	1	72	0.433	EQ	25	1	5.5	10
10	62	T12	1	1	2	1	1	97	0.883	EQ	25	1	5.5	10
10	62	T18	1	1	1	2	1	97	0.796	EQ	25	1	5.5	10
10	62	T3	1	1	2	2	1	88	0.76	EQ	25	1	5.5	10
10	62	T6	1	1	1	1	1	96	1	EQ	25	1	5.5	10
10	61	T0.5	2	3	3	2	1	67	0.15	EQ	30	1	19	28
10	61	T12	1	1	1	1	1	100	1	EQ	30	1	19	28
10	61	T18	1	1	1	1	1	97	1	EQ	30	1	19	28
10	61	T6	1	1	1	1	1	96	1	EQ	30	1	19	28
10	60	T0.5	1	1	2	2	1	40	0.76	EQ	19	0	4.6	24
10	59	T0.5	1	2	3	2	1	67	0.329	EQ	25	0	6.8	24
10	59	T12	1	1	1	2	2	78	0.725	EQ	25	0	6.8	24
10	59	T18	1	1	3	2	3	53	0.197	EQ	25	0	6.8	24
10	59	T3	1	1	1	2	2	50	0.725	EQ	25	0	6.8	24
10	59	T6	1	1	1	2	3	55	0.291	EQ	25	0	6.8	24
10	58	T0.5	1	1	3	2	2	90	0.362	EQ	58	1	38.5	33
10	58	T12	1	1	1	2	1	70	0.796	EQ	58	1	38.5	33
10	58	T18	1	1	1	1	1	90	1	EQ	58	1	38.5	33
10	58	T3	2	1	2	2	1	70	0.691	EQ	58	1	38.5	33
10	58	T6	1	1	1	2	1	90	0.796	EQ	58	1	38.5	33
10	57	T0.5	2	2	2	2	2	67	0.516	EQ	43	1	2	20
10	56	T0.5	3	2	3	1	1	71	0.138	EQ	43	0	7.2	24
10	56	T12	1	1	1	1	1	75	1	EQ	43	0	7.2	24
10	56	T18	1	1	1	1	1	77	1	EQ	43	0	7.2	24
10	56	T3	1	1	1	2	3	63	0.291	EQ	43	0	7.2	24
10	56	T6	1	1	1	2	2	70	0.725	EQ	43	0	7.2	24
10	55	T0.5	1	1	1	2	1	90	0.796	EQ	40	0	9	24
10	54	T0.5	3	2	3	2	2	61	-0.056	EQ	24	0	6.1	19
10	53	T0.5	2	1	2	2	1	80	0.691	EQ	42	1	7.5	16
10	52	T0.5	2	2	3	2	2	75	0.189	EQ	26	0	3.9	10
10	52	T12	1	1	2	3	3	38	-0.008	EQ	26	0	3.9	10
10	52	T18	1	1	1	2	3	70	0.291	EQ	26	0	3.9	10
10	52	T3	1	1	3	2	2	70	0.362	EQ	26	0	3.9	10
10	52	T6	1	1	2	2	2	75	0.689	EQ	26	0	3.9	10
10	50	T0.5	3	1	3	1	1	65	0.242	EQ	55	1	3	22
10	50	T12	1	1	1	1	1	100	1	EQ	55	1	3	22
10	50	T18	2	1	2	2	1	90	0.691	EQ	55	1	3	22

10	50	T3	2	1	2	2	1	75	0.691	EQ	55	1	3	22
10	50	T6	1	1	1	1	1	100	1	EQ	55	1	3	22
10	49	T0.5	3	1	3	2	2	60	0.048	EQ	50	1	7	20
10	49	T12	1	1	1	1	1	84	1	EQ	50	1	7	20
10	49	T18	2	1	1	1	1	90	0.85	EQ	50	1	7	20
10	49	T3	2	1	2	1	1	65	0.814	EQ	50	1	7	20
10	49	T6	2	1	2	2	1	68	0.691	EQ	50	1	7	20
10	48	T0.5	1	1	2	2	1	88	0.76	EQ	55	1	9.6	23
10	48	T12	1	1	1	2	1	95	0.796	EQ	55	1	9.6	23
10	48	T18	1	1	1	1	2	94	0.848	EQ	55	1	9.6	23
10	48	T3	1	1	1	2	1	95	0.796	EQ	55	1	9.6	23
10	48	T6	1	1	1	2	1	95	0.796	EQ	55	1	9.6	23
10	46	T0.5	2	2	2	3	1	30	0.055	EQ	25	0	6.5	22
10	46	T12	1	1	1	1	1	100	1	EQ	25	0	6.5	22
10	46	T18	1	1	1	1	1	64	1	EQ	25	0	6.5	22
10	46	T3	1	1	1	1	1	90	1	EQ	25	0	6.5	22
10	46	T6	1	1	1	1	1	95	1	EQ	25	0	6.5	22
10	45	T0.5	2	1	2	2	1	92	0.691	EQ	61	1	7.5	13
10	45	T12	1	1	1	2	2	70	0.725	EQ	61	1	7.5	13
10	45	T6	2	1	2	1	1	75	0.814	EQ	61	1	7.5	13
10	44	T0.5	1	1	1	2	1	99	0.796	EQ	64	1	6	9
10	44	T12	1	1	1	1	1	100	1	EQ	64	1	6	9
10	44	T18	1	1	1	1	1	100	1	EQ	64	1	6	9
10	44	T3	1	1	1	1	1	100	1	EQ	64	1	6	9
10	44	T6	1	1	1	1	1	100	1	EQ	64	1	6	9
10	43	T0.5	3	2	3	2	3	61	-0.221	EQ	31	1	19.2	21
10	43	T12	1	1	1	2	3	54	0.291	EQ	31	1	19.2	21
10	43	T18	1	1	1	2	2	85	0.725	EQ	31	1	19.2	21
10	43	T3	1	1	1	2	1	90	0.796	EQ	31	1	19.2	21
10	43	T6	1	1	1	2	2	90	0.725	EQ	31	1	19.2	21
10	42	T0.5	3	2	3	2	2	58	-0.056	EQ	49	0	1.8	27
10	42	T12	1	1	2	2	2	60	0.689	EQ	49	0	1.8	27
10	42	T18	1	1	1	2	2	70	0.725	EQ	49	0	1.8	27
10	42	T3	1	1	1	2	1	70	0.796	EQ	49	0	1.8	27
10	42	T6	1	1	1	2	2	70	0.725	EQ	49	0	1.8	27
10	41	T0.5	1	2	3	2	1	90	0.329	EQ	67	1	19	39
10	41	T12	1	1	1	1	1	35	1	EQ	67	1	19	39
10	41	T3	1	2	2	2	1	90	0.656	EQ	67	1	19	39
10	41	T6	1	1	1	1	1	40	1	EQ	67	1	19	39
10	40	T0.5	1	1	1	1	1	95	1	EQ	23	1	6.5	17
10	39	T0.5	1	1	2	2	2	32	0.689	EQ	51	1	3.1	21
10	39	T3	2	1	1	2	2	70	0.656	EQ	51	1	3.1	21
10	38	T0.5	2	2	2	2	1	60	0.587	EQ	20	0	5	14
10	38	T3	1	1	1	2	2	80	0.725	EQ	20	0	5	14
10	38	T6	1	1	1	2	2	80	0.725	EQ	20	0	5	14
10	37	T0.5	2	2	3	2	1	58	0.26	EQ	69	1	20	28
10	37	T12	1	2	2	2	1	85	0.656	EQ	69	1	20	28
10	37	T3	1	2	2	2	1	80	0.656	EQ	69	1	20	28
10	37	T6	1	2	2	2	1	80	0.656	EQ	69	1	20	28
10	36	T0.5	2	1	2	2	2	80	0.62	EQ	66	0	7	20
10	36	T12	2	1	1	1	2	80	0.779	EQ	66	0	7	20

10	36	T3	1	1	1	2	2	80	0.725	EQ	66	0	7	20
10	36	T6	1	1	1	1	2	70	0.848	EQ	66	0	7	20
10	35	T0.5	2	2	2	2	1	98	0.587	EQ	38	1	4	1
10	35	T3	1	1	1	2	1	95	0.796	EQ	38	1	4	1
10	35	T6	1	1	1	2	1	100	0.796	EQ	38	1	4	1
10	34	T0.5	2	2	2	1	1	75	0.71	EQ	90	1	35	13
10	34	T12	1	1	1	1	1	80	1	EQ	90	1	35	13
10	34	T3	1	1	1	1	1	80	1	EQ	90	1	35	13
10	34	T6	2	1	2	2	1	65	0.691	EQ	90	1	35	13
10	33	T0.5	2	3	3	2	1	50	0.15	EQ	62	0	39	80
10	33	T12	1	1	2	1	1	80	0.883	EQ	62	0	39	80
10	33	T18	1	1	1	1	1	70	1	EQ	62	0	39	80
10	33	T3	1	2	2	1	1	50	0.779	EQ	62	0	39	80
10	33	T6	1	2	3	2	1	50	0.329	EQ	62	0	39	80
10	32	T0.5	1	1	2	2	1	70	0.76	EQ	53	1	23	10
10	31	T0.5	3	2	3	2	1	60	0.015	EQ	53	1	4	13
10	31	T12	1	1	2	2	1	90	0.76	EQ	53	1	4	13
10	31	T18	1	1	1	1	1	100	1	EQ	53	1	4	13
10	31	T3	2	1	2	2	1	70	0.691	EQ	53	1	4	13
10	31	T6	1	1	1	1	1	90	1	EQ	53	1	4	13
10	30	T0.5	1	1	3	1	1	100	0.556	EQ	21	1	2.5	3
10	29	T0.5	2	2	2	2	1	79	0.587	EQ	49	1	5.5	2
10	29	T12	1	1	1	1	1	98	1	EQ	49	1	5.5	2
10	29	T18	1	1	1	1	1	96	1	EQ	49	1	5.5	2
10	29	T6	1	1	1	1	1	85	1	EQ	49	1	5.5	2
10	28	T0.5	3	3	3	2	1	40	-0.095	EQ	67	0	14	28
10	28	T12	1	1	1	2	1	60	0.796	EQ	67	0	14	28
10	28	T18	1	1	1	2	1	70	0.796	EQ	67	0	14	28
10	28	T3	1	1	2	2	1	60	0.76	EQ	67	0	14	28
10	28	T6	1	1	1	2	1	50	0.796	EQ	67	0	14	28
10	25	T0.5	1	1	1	1	1	100	1	EQ	38	0	4	4
10	24	T0.5	1	1	2	2	1	70	0.76	EQ	52	1	10	6
10	24	T12	1	1	1	1	1	95	1	EQ	52	1	10	6
10	24	T18	1	1	1	1	1	80	1	EQ	52	1	10	6
10	24	T3	1	1	1	1	1	80	1	EQ	52	1	10	6
10	24	T6	1	1	1	2	1	70	0.796	EQ	52	1	10	6
10	23	T0.5	1	1	1	1	1	100	1	EQ	47	0	4	13
10	23	T12	1	1	1	1	1	100	1	EQ	47	0	4	13
10	23	T3	1	1	1	1	1	100	1	EQ	47	0	4	13
10	23	T6	1	1	1	1	1	100	1	EQ	47	0	4	13
10	22	T0.5	2	2	3	2	1	#N/B	0.26	EQ	48	1	9	24
10	22	T12	1	1	1	1	1	80	1	EQ	48	1	9	24
10	21	T0.5	1	1	1	1	2	50	0.848	EQ	36	1	10	5
10	21	T12	1	1	1	1	2	70	0.848	EQ	36	1	10	5
10	21	T18	1	1	1	2	2	65	0.725	EQ	36	1	10	5
10	21	T3	1	1	1	2	1	60	0.796	EQ	36	1	10	5
10	21	T6	1	1	1	1	1	70	1	EQ	36	1	10	5
10	20	T0.5	2	2	2	3	2	32	-0.016	EQ	42	0	5	16
10	20	T12	2	1	1	2	1	85	0.727	EQ	42	0	5	16
10	20	T3	2	1	2	2	2	25	0.62	EQ	42	0	5	16
10	20	T6	2	1	2	2	1	40	0.691	EQ	42	0	5	16

10	19	T12	1	1	1	2	1	80	0.796	EQ	41	0	8	24
10	19	T18	1	1	1	1	1	80	1	EQ	41	0	8	24
10	19	T3	2	1	1	2	1	80	0.727	EQ	41	0	8	24
10	19	T6	1	1	1	2	1	75	0.796	EQ	41	0	8	24
10	18	T0.5	1	2	2	2	1	80	0.656	EQ	26	1	35	43
10	18	T12	1	1	2	2	2	75	0.689	EQ	26	1	35	43
10	18	T18	1	1	2	2	1	82	0.76	EQ	26	1	35	43
10	18	T3	1	2	2	2	1	55	0.656	EQ	26	1	35	43
10	18	T6	1	1	2	2	2	75	0.689	EQ	26	1	35	43
10	17	T0.5	3	2	3	2	1	60	0.015	EQ	32	1	23	36
10	17	T12	1	1	1	1	1	85	1	EQ	32	1	23	36
10	17	T18	1	1	1	1	1	95	1	EQ	32	1	23	36
10	17	T3	2	1	2	2	1	75	0.691	EQ	32	1	23	36
10	17	T6	2	1	2	1	1	80	0.814	EQ	32	1	23	36
10	16	T0.5	3	2	3	1	1	80	0.138	EQ	35	0	2.5	27
10	16	T12	2	1	2	1	2	80	0.743	EQ	35	0	2.5	27
10	16	T18	1	1	1	1	2	80	0.848	EQ	35	0	2.5	27
10	16	T3	2	1	2	1	2	85	0.743	EQ	35	0	2.5	27
10	16	T6	2	1	2	1	2	80	0.743	EQ	35	0	2.5	27
10	14	T0.5	2	1	2	2	1	70	0.691	EQ	56	0	9	5
10	14	T12	1	1	1	1	1	85	1	EQ	56	0	9	5
10	14	T18	1	1	1	1	1	90	1	EQ	56	0	9	5
10	14	T3	1	1	1	1	1	#N/B	1	EQ	56	0	9	5
10	14	T6	1	1	1	1	1	85	1	EQ	56	0	9	5
10	13	T0.5	1	1	1	1	1	90	1	EQ	66	0	4.5	25
10	13	T12	1	1	2	1	1	95	0.883	EQ	66	0	4.5	25
10	13	T18	2	1	2	1	2	90	0.743	EQ	66	0	4.5	25
10	13	T3	1	1	1	1	2	90	0.848	EQ	66	0	4.5	25
10	13	T6	1	1	2	1	1	90	0.883	EQ	66	0	4.5	25
10	12	T0.5	2	1	2	1	1	85	0.814	EQ	33	1	36.5	29
10	12	T3	1	1	1	1	2	90	0.848	EQ	33	1	36.5	29
10	11	T0.5	2	2	2	2	1	70	0.587	EQ	32	0	32	32
10	11	T12	1	3	3	3	1	60	-0.044	EQ	32	0	32	32
10	11	T18	1	1	1	2	2	70	0.725	EQ	32	0	32	32
10	11	T3	1	2	3	2	1	75	0.329	EQ	32	0	32	32
10	11	T6	1	2	2	2	1	70	0.656	EQ	32	0	32	32
10	10	T12	1	1	1	1	1	80	1	EQ	24	0	13	15
10	10	T3	1	1	1	1	1	99	1	EQ	24	0	13	15
10	10	T6	1	1	1	1	1	90	1	EQ	24	0	13	15
10	9	T0.5	1	1	1	1	1	90	1	EQ	41	1	1	27
10	9	T3	2	1	1	1	1	100	0.85	EQ	41	1	1	27
10	9	T6	1	1	1	1	1	100	1	EQ	41	1	1	20
10	8	T0.5	2	3	3	2	2	75	0.079	EQ	63	1	5	48
10	8	T18	1	1	1	1	2	85	0.848	EQ	63	1	5	48
10	8	T3	1	2	2	2	1	80	0.656	EQ	63	1	5	48
10	8	T6	1	1	2	1	2	75	0.812	EQ	63	1	5	48
10	7	T0.5	2	2	3	2	1	80	0.26	EQ	23	1	8	18
10	7	T12	1	1	1	2	1	89	0.796	EQ	23	1	8	18
10	7	T18	1	1	1	2	1	90	0.796	EQ	23	1	8	18
10	7	T3	2	1	2	2	1	85	0.691	EQ	23	1	8	18
10	7	T6	1	1	1	2	1	85	0.796	EQ	23	1	8	18

10	6	T0.5	1	1	1	1	2	65	0.848	EQ	60	0	10	27
10	6	T12	1	1	2	1	1	50	0.883	EQ	60	0	10	27
10	6	T18	1	1	1	1	1	80	1	EQ	60	0	10	27
10	6	T3	1	1	3	2	1	60	0.433	EQ	60	0	10	27
10	5	T0.5	1	3	3	2	2	70	0.148	EQ	44	0	2	2
10	5	T3	1	2	2	2	3	50	0.151	EQ	44	0	2	2
10	4	T0.5	1	1	1	1	1	75	1	EQ	58	1	11	12
10	4	T12	1	1	1	1	1	90	1	EQ	58	1	11	12
10	4	T3	1	1	1	1	1	80	1	EQ	58	1	11	12
10	4	T6	1	1	1	1	1	95	1	EQ	58	1	11	12
10	3	T12	1	1	1	1	1	100	1	EQ	62	1	7	4
10	3	T18	1	1	1	1	1	100	1	EQ	62	1	7	4
10	3	T3	1	1	2	1	1	100	0.883	EQ	62	1	7	4
10	3	T6	1	1	1	1	1	100	1	EQ	62	1	7	4
10	2	T0.5	3	2	3	3	2	35	-0.319	EQ	23	1	8.5	19
10	2	T3	1	1	1	3	1	80	0.264	EQ	23	1	8.5	19
10	2	T6	1	1	1	1	1	90	1	EQ	23	1	8.5	19
10	1	T0.5	3	2	1	1	1	#N/B	0.232	EQ	20	1	9.5	2
10	1	T12	1	1	1	1	1	95	1	EQ	20	1	9.5	2
10	1	T18	1	1	1	1	1	94	1	EQ	20	1	9.5	2
10	1	T3	1	2	2	2	2	84	0.585	EQ	20	1	9.5	2
10	1	T6	1	1	1	2	1	85	0.796	EQ	20	1	9.5	2
