# Supplementary material for: Recovery of health-related quality of life after burn injuries: An individual participant data meta-analysis
Source: PLoS One. 2020 Jan 10;15(1):e0226653. doi: 10.1371/journal.pone.0226653 (PMC6953837; doi:10.1371/journal.pone.0226653)
Supplement: S2 Appendix — (PDF) [file pone.0226653.s002.pdf]

**S2 Appendix. The 24-month recovery of health-related quality of life (EQ-5D utility) model**

| Random effects | Variance | SD |
|---|---|---|
| Patient:study | 0.022 | 0.147 |
| Study | 0.007 | 0.081 |
| Residual | 0.036 | 0.190 |

| Fixed effects | Estimate | SE | P value |
|---|---|---|---|
| Intercept | 0.876 | 0.042 | <0.001 |
| Timepoint 0 months | -0.665 | 0.070 | <0.001 |
| Timepoint 0.5 months | -0.217 | 0.051 | <0.001 |
| Timepoint 0.75 months | -0.176 | 0.045 | <0.001 |
| Timepoint 3 months | -0.0884 | 0.031 | 0.005 |
| Timepoint 6 months | -0.0872 | 0.034 | 0.010 |
| Timepoint 9 months | 0.038 | 0.047 | 0.418 |
| Timepoint 12 months | -0.020 | 0.029 | 0.494 |
| Timepoint 18 months | -0.033 | 0.037 | 0.371 |
| Gender | 0.003 | 0.027 | 0.925 |
| Length of hospital stay (LOS) | -0.013 | 0.004 | <0.001 |
| %TBSA burned | 0.007 | 0.005 | 0.173 |
| Age | -0.013 | 0.004 | <0.001 |
| Gender*Timepoint 0 months | 0.324 | 0.068 | <0.001 |
| Gender*Timepoint 0.5 months | 0.020 | 0.048 | 0.682 |
| Gender*Timepoint 0.75 months | 0.075 | 0.042 | 0.079 |
| Gender*Timepoint 3 months | 0.077 | 0.031 | 0.013 |
| Gender*Timepoint 6 months | 0.081 | 0.034 | 0.018 |
| Gender*Timepoint 9 months | 0.027 | 0.045 | 0.541 |
| Gender*Timepoint 12 months | 0.014 | 0.030 | 0.643 |
| Gender*Timepoint 18 months | 0.057 | 0.036 | 0.114 |
| LOS*Timepoint 0 months | 0.024 | 0.008 | 0.003 |
| LOS*Timepoint 0.5 months | -0.085 | 0.012 | <0.001 |
| LOS*Timepoint 0.75 months | -0.015 | 0.007 | 0.029 |
| LOS*Timepoint 3 months | -0.008 | 0.005 | 0.070 |
| LOS*Timepoint 6 months | 0.006 | 0.005 | 0.207 |
| LOS*Timepoint 9 months | -0.018 | 0.008 | 0.022 |
| LOS*Timepoint 12 months | 0.003 | 0.004 | 0.456 |
| LOS*Timepoint 18 months | -0.001 | 0.006 | 0.914 |
| TBSA*Timepoint 0 months | -0.047 | 0.010 | <0.001 |
| TBSA*Timepoint 0.5 months | 0.038 | 0.014 | 0.009 |
| TBSA*Timepoint 0.75 months | -0.041 | 0.010 | <0.001 |
| TBSA*Timepoint 3 months | -0.019 | 0.006 | 0.002 |
| TBSA*Timepoint 6 months | -0.016 | 0.006 | 0.013 |
| TBSA*Timepoint 9 months | -0.016 | 0.011 | 0.147 |
| TBSA*Timepoint 12 months | -0.012 | 0.005 | 0.040 |
| TBSA*Timepoint 18 months | -0.006 | 0.008 | 0.424 |
